# Incorporation of β‐Alanine in Cu(II) ATCUN Peptide Complexes Increases ROS Levels, DNA Cleavage and Antiproliferative Activity**

**DOI:** 10.1002/chem.202102601

**Published:** 2021-12-04

**Authors:** Julian Heinrich, Karolina Bossak‐Ahmad, Mie Riisom, Haleh H. Haeri, Tasha R. Steel, Vinja Hergl, Alexander Langhans, Corinna Schattschneider, Jannis Barrera, Stephen M. F. Jamieson, Matthias Stein, Dariush Hinderberger, Christian G. Hartinger, Wojciech Bal, Nora Kulak

**Affiliations:** ^1^ Institute of Chemistry and Biochemistry Freie Universität Berlin Fabeckstr. 34/36 14195 Berlin Germany; ^2^ Institute of Chemistry Otto-von-Guericke-Universität Magdeburg Universitätsplatz 2 39106 Magdeburg Germany; ^3^ Institute of Biochemistry and Biophysics Polish Academy of Science Pawińskiego 5a 02-106 Warsaw Poland; ^4^ School of Chemical Sciences University of Auckland Private Bag 92019 Auckland 1142 New Zealand; ^5^ Institute of Chemistry Martin-Luther-Universität Halle-Wittenberg Von-Danckelmann-Platz 4 06120 Halle Germany; ^6^ Department of Chemistry Humboldt-Universität zu Berlin Brook-Taylor-Strasse 2 12489 Berlin Germany; ^7^ Auckland Cancer Society Research Centre University of Auckland Private Bag 92019 Auckland 1142 New Zealand; ^8^ Max Planck Institute for Dynamics of Complex Technical Systems Sandtorstrasse 1 39106 Magdeburg Germany

**Keywords:** ATCUN peptides, copper, cytotoxicity, DNA cleavage, reactive oxygen species

## Abstract

Redox‐active Cu(II) complexes are able to form reactive oxygen species (ROS) in the presence of oxygen and reducing agents. Recently, Faller et al. reported that ROS generation by Cu(II) ATCUN complexes is not as high as assumed for decades. High complex stability results in silencing of the Cu(II)/Cu(I) redox cycle and therefore leads to low ROS generation. In this work, we demonstrate that an exchange of the α‐amino acid Gly with the β‐amino acid β‐Ala at position 2 (Gly2→β‐Ala2) of the ATCUN motif reinstates ROS production (^•^OH and H_2_O_2_). Potentiometry, cyclic voltammetry, EPR spectroscopy and DFT simulations were utilized to explain the increased ROS generation of these β‐Ala2‐containing ATCUN complexes. We also observed enhanced oxidative cleavage activity towards plasmid DNA for β‐Ala2 compared to the Gly2 complexes. Modifications with positively charged Lys residues increased the DNA affinity through electrostatic interactions as determined by UV/VIS, fluorescence, and CD spectroscopy, and consequently led to a further increase in nuclease activity. A similar trend was observed regarding the cytotoxic activity of the complexes against several human cancer cell lines where β‐Ala2 peptide complexes had lower IC_50_ values compared to Gly2. The higher cytotoxicity could be attributed to an increased cellular uptake as determined by ICP‐MS measurements.

## Graphical abstract

**Generation of reactive oxygen species** was achieved with a redox‐silent Cu(II) ATCUN complex by replacing the α‐amino acid glycine with β‐alanine. Increased ROS production led to higher oxidative DNA cleavage activity. The reasons for this higher activity were elucidated via potentiometry, cyclic voltammetry, EPR, UV/VIS spectroscopy and DFT. In cytotoxicity assays, β‐alanine peptide complexes showed lower IC_50_ values in several cancer cell lines compared with the glycine analogs, which could be correlated with the amounts of Cu taken up by cells.

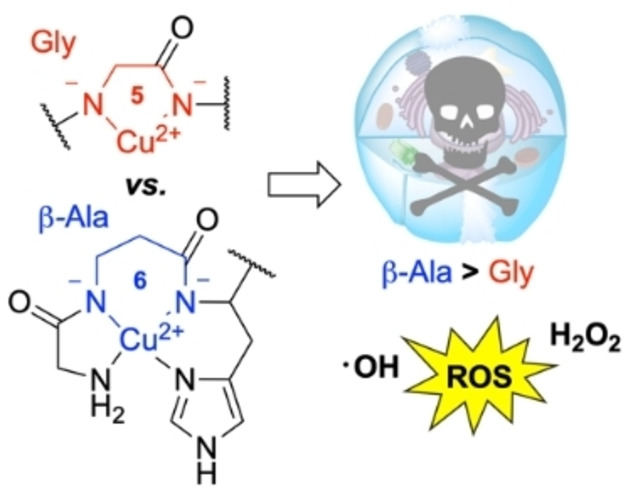

## Introduction

In all living cellular organisms, the blueprint of life is stored in DNA.^[1]^ This genetic information is essential for protein biosynthesis following the central dogma of molecular biology.^[2]^ The importance of DNA for cell proliferation makes it a promising target for the treatment of cancer.^[3]^ Cisplatin is one of the most commonly used chemotherapeutic agents, and targets DNA where it causes alterations through crosslinking and subsequently induces apoptosis. Disadvantages of its clinical application are severe side effects, such as nephro‐ and neurotoxicity.^[4]^ To overcome such side effects current research is focused on anticancer agents based on endogenous metals, such as Cu, potentially leading to lower systemic toxicity.^[5]^

For decades, Cu(II) complexes with *N*‐donor ligands have been studied for their efficient DNA cleavage properties.^[6]^ In most cases, the nuclease activity is accomplished either in a hydrolytic or an oxidative manner.^[7]^ Oxidative metallonucleases induce irreversible double‐strand breaks in DNA through formation of reactive oxygen species (ROS).^[8]^ This process leads not only to an alteration of the DNA double strand, as with cisplatin, but rather degradation of the biomolecule. As a result, such Cu(II) complexes are promising tools for the development of novel and efficient chemotherapeutics.^[9]^

**Figure 1:**
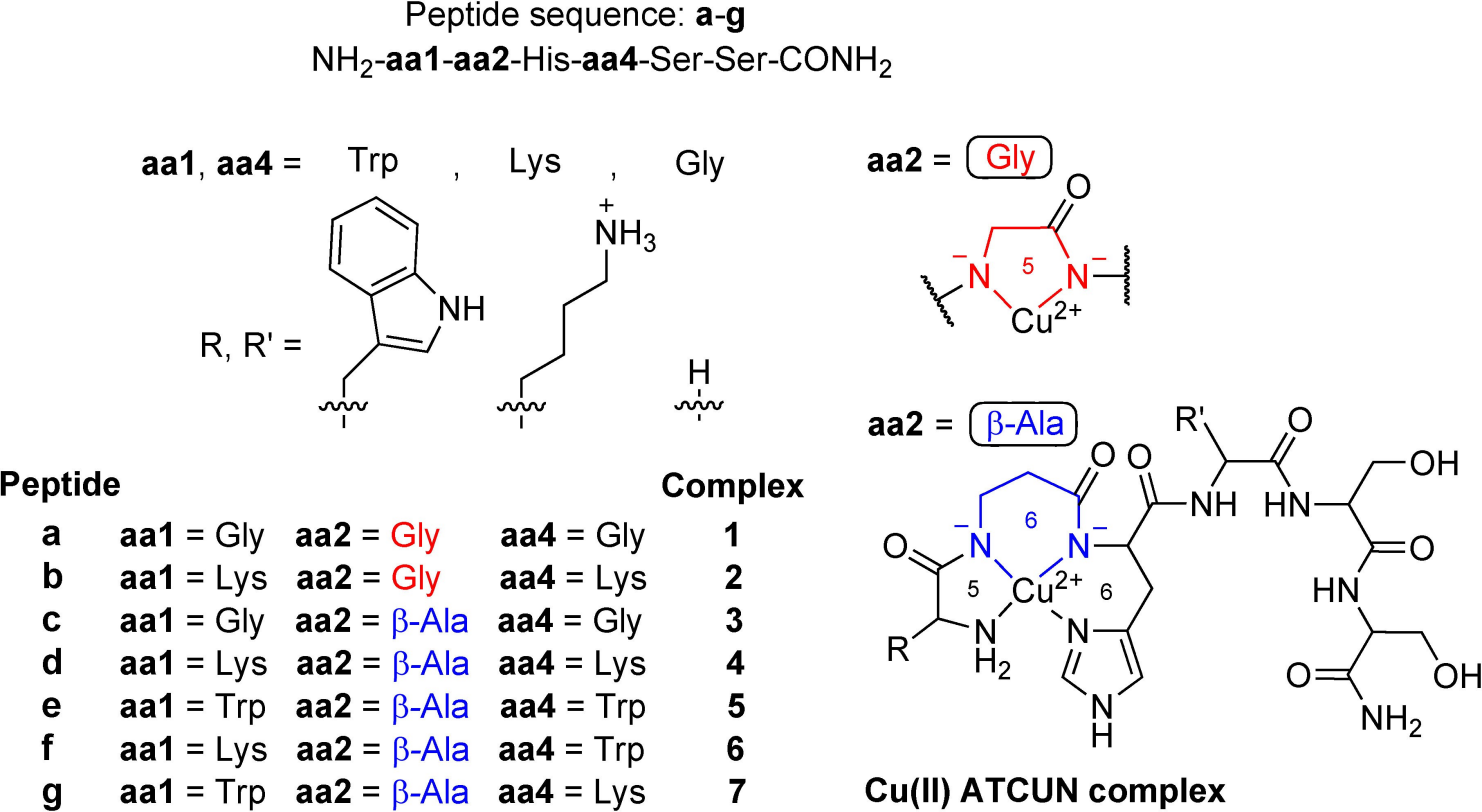
Cu(II) complexes **1**–**7** of ATCUN peptides **a**–**g**. Amino acid **aa2** is either Gly (α‐amino acid, 5‐membered chelate) or β‐Ala (β‐amino acid, 6‐membered chelate), whereas **aa1** and **aa4** are either Gly, Lys or Trp.

The ATCUN motif (amino terminal Cu(II) and Ni(II) binding) occurs naturally at the *N*‐terminus of albumins,^[10]^ the Cu transport protein Ctr1,^[11]^ neuromedin C^[12]^ and several other (neuro)hormones and immune system‐related peptides.^[13]^ Under physiological conditions it binds Cu(II) in a square planar fashion (4N chelating ligand) through the amine of the *N*‐terminal amino acid, two deprotonated amide bonds and the δ‐N atom of the imidazole moiety of His (N_im_) in position 3 (Figure 1).[^10^, ^11^, ^12^, ^13^, ^14^]

In 1983, Pauling et al. showed that the Cu(II) complex with the simplest ATCUN motif, the tripeptide NH_2_−Gly−Gly−His−COOH (Cu−GGH), killed Ehrlich ascites tumor cells in vivo, and cleaved DNA in the presence of the reducing agent ascorbate (ascH^−^) and O_2_.^[15]^ Cowan et al. reported Cu(II) ATCUN complexes with anticancer, ‐viral, and ‐microbial activity as well as for DNA cleavage and enzyme inhibition.[^3^, ^16^, ^17^]

Regarding the DNA cleavage induced by Cu(II) ATCUN complexes, the redox activity of the metal is responsible for ROS generation. Thereby, current literature reveals conflicting statements whether the redox couple Cu(II)/Cu(I) (reduction) or Cu(II)/Cu(III) (oxidation) initiates ROS formation at physiological pH. The pathway through Cu(I) is generally favored though in the literature.^[14]^

At pH 7.4, the stability constant of Cu(II) complexes with ATCUN peptides consisting of α‐amino acids like Gly−Gly−His, is very high (log *K*_7.4_=12.4, competitivity index (CI) method).^[14]^ The square planar coordination geometry results in the formation of stable chelates with a different number of ring atoms (5,5,6) (Figure 1).^[18]^ The switch to a tetrahedral Cu(I) coordination is unfavorable due to the non‐flexible chelate ring arrangement. Indeed, such complexes do not show electrochemical Cu(II) reduction below −1.0 V vs. NHE.[^14^, ^19^] Thus, Cu(II) is not efficiently reduced by ascH^−^.[^14^, ^19^, ^20^] Nevertheless, the Bal group recently identified a long‐lived (100 ms) Cu(II) 2N intermediate species (terminal NH_2_+N_im_) during the formation of Cu(II) 4N‐ATCUN complexes, which is able to maintain the Cu(II)/Cu(I) redox pair at pH 6. This fact corroborates the ROS generation pathway via Cu(I) at pH 7.4.^[21]^

For most Cu(III) complexes a square planar coordination geometry has been observed.^[22]^ Thus, ROS generation by Cu(II) ATCUN complexes through oxidation of Cu(II) to Cu(III) by O_2_ ^[14]^ conserving the square planar geometry should be more convenient. However, the Cu(III)/Cu(II) redox couple for ATCUN complexes (+0.87 to +1.07 V vs. NHE, depending on the α‐amino acid sequence)^[14]^ is electrochemically not accessible for the ascH^−^/asc^.−^ redox couple (or: ascH^−^/dehydroascorbate, 2 e^−^/2 H^+^ process).^[20]^

Although Cu(II) ATCUN complexes with α‐amino acids indeed can act as nucleases through ROS generation,[^3^, ^14^, ^16^] Faller et al. recently demonstrated that the catalytic ROS generation is not as efficient as previously estimated.^[23]^ The low catalytic activity is due to high Cu(II) complex stability, which results in redox silencing of Cu(II).[^18^, ^23^]

A very important and so far unanswered question in the light of the current literature is, whether the catalytic ROS generation by Cu(II) ATCUN complexes can be increased through structural changes in the ATCUN peptide. Here, we give an insight into how the incorporation of β‐Ala in position 2 of Cu(II) ATCUN peptides in comparison to Gly (5,6,6 vs. 5,5,6 chelates) influences complex stability, redox and coordination chemistry and therefore ROS generation. Additionally, amino acids with DNA‐affine moieties, Lys and Trp, were incorporated leading to Cu(II) ACTUN peptides **1**–**7** (Figure 1). These complexes were thoroughly characterized and evaluated regarding their biological activity including sophisticated computational chemistry methods.

## Results and Discussion

### Synthesis of ATCUN peptides and Cu(II) complexes

The novel ATCUN peptides **a**–**g** were synthesized from L‐amino acids via manual solid‐phase peptide synthesis using the Fmoc strategy,^[24]^ purified by RP‐HPLC, and characterized by ESI‐MS and analytical RP‐HPLC (S‐2). Peptide yields were determined by UV/VIS spectroscopy (S‐3). The amino acids at positions 1 and 4 are either Gly, Lys and Trp, respectively, for facilitating DNA interaction. A hydrophilic Ser‐Ser tail at the amidated C‐terminus ensures water solubility. Additionally, position 2 contains either the α‐amino acid Gly (Gly2) or the β‐amino acid β‐Ala (β‐Ala2) to provide either a 5‐membered or a 6‐membered chelate ring within the Cu(II) coordination sphere.

The corresponding Cu(II) ATCUN complexes **1**–**7** (Figure 1) were prepared in situ and characterized by ESI‐MS and UV/VIS spectroscopy (S‐3–S‐4). While a Cu(II) ATCUN peptide with Gly2 and Lys in positions 1 and 4 (**2**) has been investigated before,[^16^, ^25^, ^26^] it did not feature a Ser‐Ser tail.

### Stability constants of Cu(II) complexes at pH 7.4

**Figure 2:**
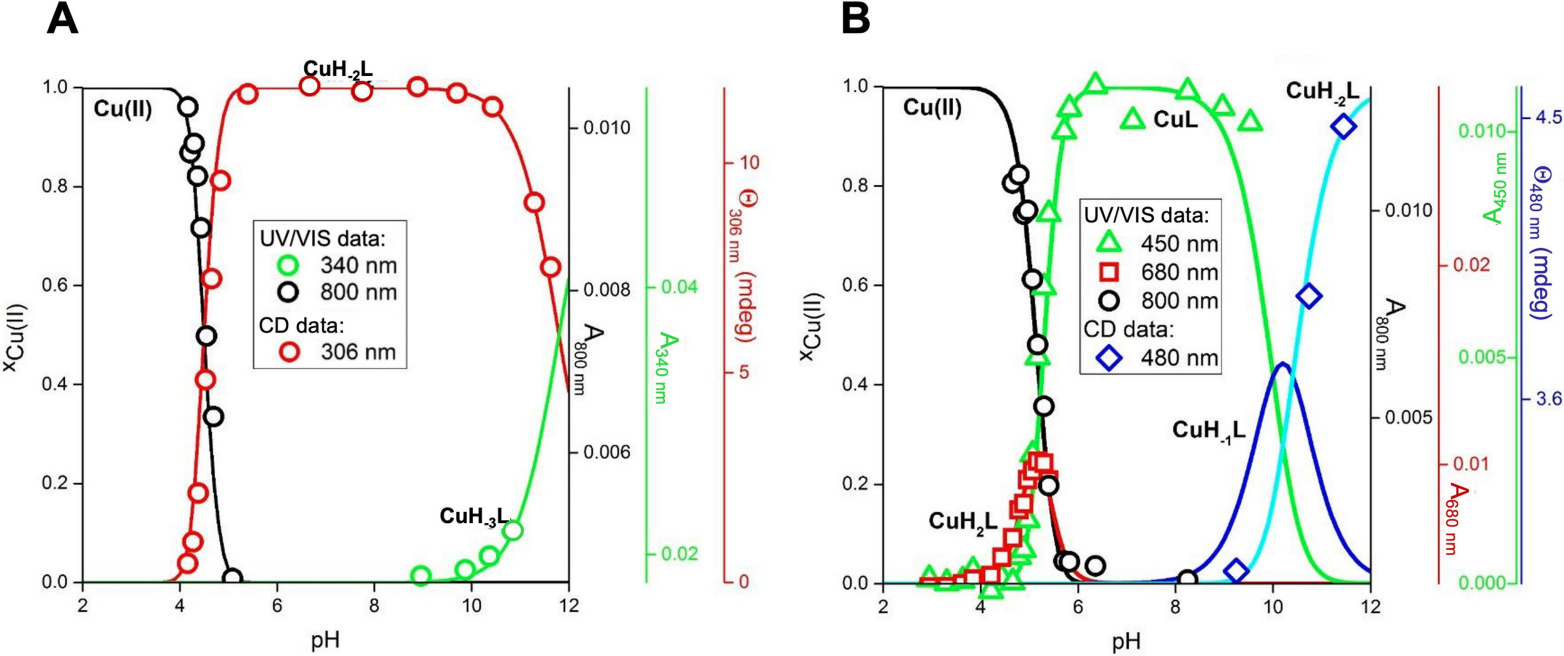
Species distribution at 25 °C for **A**: Cu(II)L (L=**a**) and **B**: Cu(II)H_2_L (H_2_L=**d**), calculated for concentrations used in UV/VIS and CD titrations (1.0 mM peptide, 0.8 mM CuCl_2_) based on stability constants (Table S5). The y axis on the left indicates molar fractions x of Cu(II) complexes, which are color‐coded as follows: [Cu(H_2_O)_6_]^2+^, black; **A**: Cu(II) 4N complexes, red and green, **B**: Cu(II) 2N complex, red; Cu(II) 4N complexes, green and blue. The y axes on the right provide values of absorbance (UV/VIS) and ellipticity (CD).

Key factors for the investigation of the biological activity of metal complexes are the complex stability and the detection of the active species at a physiological pH value of 7.4.^[27]^ Thus, potentiometric titration experiments of peptides **a**–**g** (**b** ^[26]^) alone and in the presence of CuCl_2_ (logarithmic protonation (log *β*) and protonation‐corrected Cu(II) binding constants (log *K)) and pH‐metric UV/VIS and CD spectroscopic measurements of **1**–**7** (**2** ^[26]^) were carried out (S‐5). Exemplarily, the obtained pH‐dependent species distribution diagrams of the Cu(II) complexes with peptides **a** (Gly1/2/4; 5,5,6) and **d** (β‐Ala2; Lys1/4; 5,6,6) are displayed in Figure 2. The calculated complex stabilities at pH 7.4 (competitivity index, CI_7.4_) for **1**–**7** are listed in Table 1.

**Table 1:** CI values calculated at pH 7.4 for Cu(II) complexes **1**–**7** of peptides **a**–**g** (1 mM Cu(II), peptides L and virtual competitive ligand Z) based on respective stability constants. CI is log *K*_CuZ_ fulfilling the condition Σ_ijk_([Cu_i_H_j_L_k_]=[CuZ] (S‐5.1, Table S5).^[28]^

**aa1**	**aa2**	**aa4**	Complex	CI_7.4_ [M^−1^]
Gly	Gly	Gly	**1**	13.61
Lys		Lys	**2**	12.91^based on 26^
Gly	β‐Ala	Gly	**3**	9.92
Lys		Lys	**4**	10.31
Trp		Trp	**5**	11.28
Lys		Trp	**6**	10.52
Trp		Lys	**7**	11.29

Based on potentiometric titrations and pH‐metric UV/VIS and CD spectroscopic data, the following conclusions regarding the biological activity of **1**–**7** at pH 7.4 can be drawn:

(i) Only one Cu(II) species is present at pH 7.4 (Figure 2, Figures S37–S40). These are CuH_‐2_L for **a**, **c**, and **e** (**1, 3, 5**), CuH_‐1_L for **f** and **g** (**6**, **7**) and CuL for **d** (**4**). For complex **2** at pH 7.4 the same Cu(II) species as for complex **4** (CuL) is assumed, though with Gly2.^[26]^

(ii) All Cu(II) species at pH 7.4 exhibit a 4N coordination mode (terminal NH_2_, 2x N^−^, N_im_) according to the structures shown in Figure 1 (**1**–**7**).

(iii) The varying content of Lys residues in **1**–**7** leads to different amounts of protonation sites at pH 7.4 and consequently to a different degree of charge in the Cu(II) ATCUN complexes: **1**, **3** and **5** are neutral; **2** and **4** (2x Lys) are double, **6** and **7** (1x Lys) are single positively charged.

(iv) Substantial amounts of Cu(II) species with a 2N coordination mode (terminal NH_2_ and N_im_) exist for all β‐Ala2 peptides **c**‐**g** in the pH range of 3–6, which is not the case for Gly2 peptides **a** and **b** (Figure 2, S37‐S40).

(v) Calculated CI at pH 7.4 for Cu(II) species with Gly2 peptides **a** and **b** are in the range of known protonation‐corrected stability constants of Cu(II) ATCUN complexes,^[18]^ whereas those of β‐Ala2 peptides **c**–**g** are roughly 100 to 1000 times lower.

The evidence of Cu(II) 4N−ATCUN complexes **1**–**7** being the dominant species at pH 7.4 is indispensable for further evaluation of their biological activity, in this case the DNA cleavage/binding activity, ROS generation and cytotoxicity.

### DNA cleavage activity

**Figure 3:**
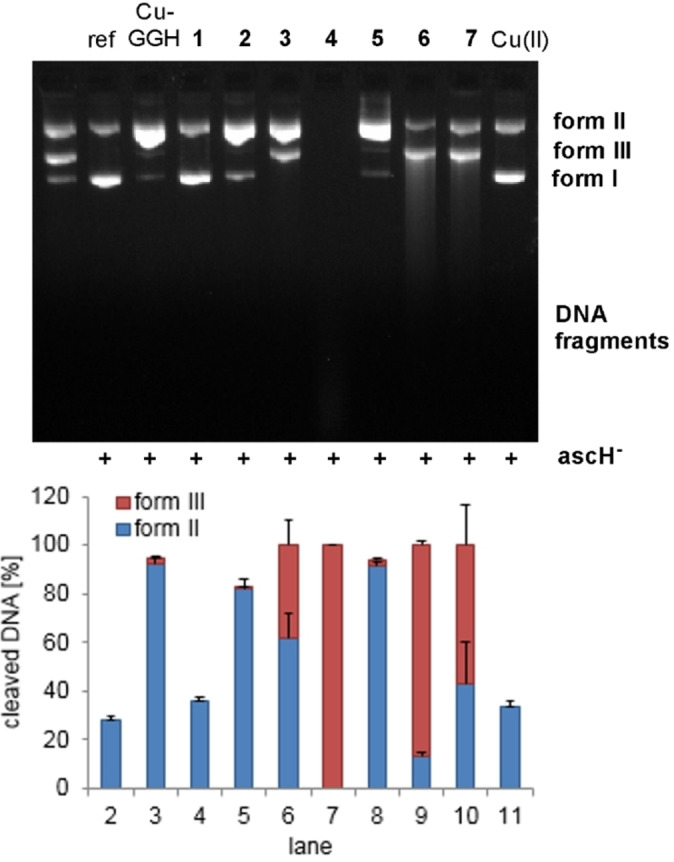
(Top) Nuclease activity towards plasmid DNA pBR322 (0.2 μg) of Cu(II) ATCUN complexes Cu−GGH, **1**–**7** (50 μM) and CuCl_2_ in MOPS buffer (50 mM, pH 7.4) in the presence of ascH^−^ (1 mM) after incubation for 1 h at 37 °C. Lane 1: DNA ladder (form I, II and III), lane 2: DNA reference, lanes 3–10 as indicated, lane 11: CuCl_2_. (Bottom) Visualization of the extent of DNA cleavage in percent. Error bars represent the standard deviation from at least three experiments.

The influence of Gly2 and β‐Ala2 in the ATCUN motif of complexes **1**–**7** on the oxidative plasmid DNA cleavage activity was tested by means of gel electrophoresis. The highly bioactive Cu(II) complex with the simplest ATCUN motif, Cu−GGH,^[15]^ was used for comparison. In Figure 3 the nuclease activity of in situ prepared **1**–**7**, Cu−GGH and CuCl_2_ in the presence of ascH^−^ as reducing agent is shown (pH 7.4, 37 °C).

The peptides **a**–**g** did not exhibit DNA cleavage activity (Figure S49), which shows that the presence of Cu(II) is essential for the nuclease activity. Indeed, Cu(II) ATCUN complexes **1**–**7** and Cu−GGH (50 μM) cleaved DNA, albeit to a different extent. The variation in nuclease activity is caused by modifications in the peptide backbone (β‐Ala2, Lys, Trp) in comparison to the parent compound Cu−GGH (Gly2) (∼90 % form II). The extension of the GGH motif with a Ser‐Ser tail (complex **1**), which is needed for complexes **5**–**7** with Trp moieties to increase the water solubility, causes an almost complete loss of activity of the corresponding Cu(II) complex (Figure 3, lanes 3 and 4). This is probably due to the different C termini, −COOH for GGH and −CONH_2_ for **a**–**g**, in which the first itself is redox‐active^[29]^ and the latter one leads to a stabilization of the 4N species.

Remarkably, the exchange of Gly2 (**1**) with β‐Ala2 (**3**) in the ATCUN motif (lanes 4 and 6) caused a significant enhancement in nuclease activity, 40 % of the plasmid DNA was found cleaved into form III. Recent research by Faller, Bal et al. showed that Gly2−ATCUN peptides lead to electrochemical redox silencing of the Cu(II)/Cu(I) couple, resulting in the complete loss of the ability to generate ROS.[^14^, ^23^] The high stability of Cu−GGH (CI_7.4_=12.4)^[14]^ and the novel Cu(II) Gly2−ATCUN complexes **1** and **2** (CI_7.4_ 13.61 and 12.91 M^−1^, Table 1) by formation of 5,5,6‐chelates is assumed to be responsible for the low propensity of catalytic ROS generation, since the Cu(II) center is “fixed” in a square planar geometry.[^14^, ^18^, ^23^] In contrast, incorporation of β‐Ala2, leading to a 5,6,6‐chelate, renders the complex less stable (CI_7.4_ 9.92–11.29 M^−1^, Table 1). The additional CH_2_ group in position 2 in the 4N‐chelating ligand provides more flexibility for the rearrangement between a square planar (Cu(II)) and a tetrahedral geometry (Cu(I)) during the catalytic redox cycle involved in the formation of ROS. The importance of the geometric flexibility of Cu(II) complexes for their oxidative nuclease activity has been previously reported.[^30^, ^31^]

The different nuclease activity of the β‐Ala2 complexes results from the DNA‐affine amino acid residues, namely the positively charged amine of Lys^[16]^ and indole of Trp.^[32]^ Indeed, the Lys1/4 modification in complex **4** drastically enhanced DNA cleavage, leading exclusively to small DNA fragments (lane 7). This activity increase due to Lys moieties has been reported before for Cu(II) Gly2−ATCUN complexes (corresponds to **1** vs. **2** in this work).[^16^, ^17^, ^25^] The Trp1/4 modification in **5** had a negative effect on the DNA cleavage activity (lane 8: 95 % form II). The β‐Ala2 complexes **6** and **7** (Lys1/Trp4 and Trp1/Lys4) exhibited DNA cleavage properties to a similar extent (lanes 9 and 10: 85/60 % form III), with their activity placed between the less active complex **5** (Trp1/4) and complex **4** (Lys1/4). The nature of the amino acid in position 1 had a slightly stronger influence on the DNA cleavage compared to modification in position 4, probably due to closer proximity to the Cu(II) center.

### DNA interaction studies

Metal complexes can stabilize the DNA double helix via various binding modes, i. e. intercalation between the nucleobases through π‐stacking, electrostatic interactions with the negatively charged phosphate backbone and groove binding.^[33]^

The DNA cleavage activity of complexes **3**–**7** varies due to the different amino acids in positions 1 and 4 (Gly/Lys/Trp). To identify the respective binding mode(s), CT‐DNA (DNA from calf thymus) and **1**–**7** were subjected to DNA melting temperature analyses (*T*_m_) by UV/VIS spectroscopy,^[34]^ ethidium bromide (EtBr) displacement by fluorescence spectroscopy,^[35]^ and circular dichroism (CD) spectroscopy.^[36]^ The results hint to weak electrostatic interactions based on Δ*T*_m_≤1.5 °C^[37]^ in DNA melting experiments (S‐7.1), *K*_app_ values <10^6^ M^−1[35,37]^ in EtBr displacement studies (S‐7.2) and only moderate changes in CD spectra (S‐7.3).[^38^, ^39^] In the CD spectra of Trp‐containing complexes **5**–**7** changes in helicity suggest groove binding as a second binding mode. Furthermore, the electrostatic DNA binding strength correlates directly with an increase in positive charge of the complexes: Lys1/4 in **2** and **4** leads to two‐fold higher positively charged complexes in comparison to the respective Gly1/4 complexes **1** and **3**. Consequently, the stronger DNA interaction results in higher nuclease activity (see above). Overall, different DNA binding affinities in the series of β‐Ala2 complexes **3**–**7** correlate well with their nuclease activity under assumption of an equal ROS production (see following section).

### Reactive oxygen species (ROS)

**Figure 4:**
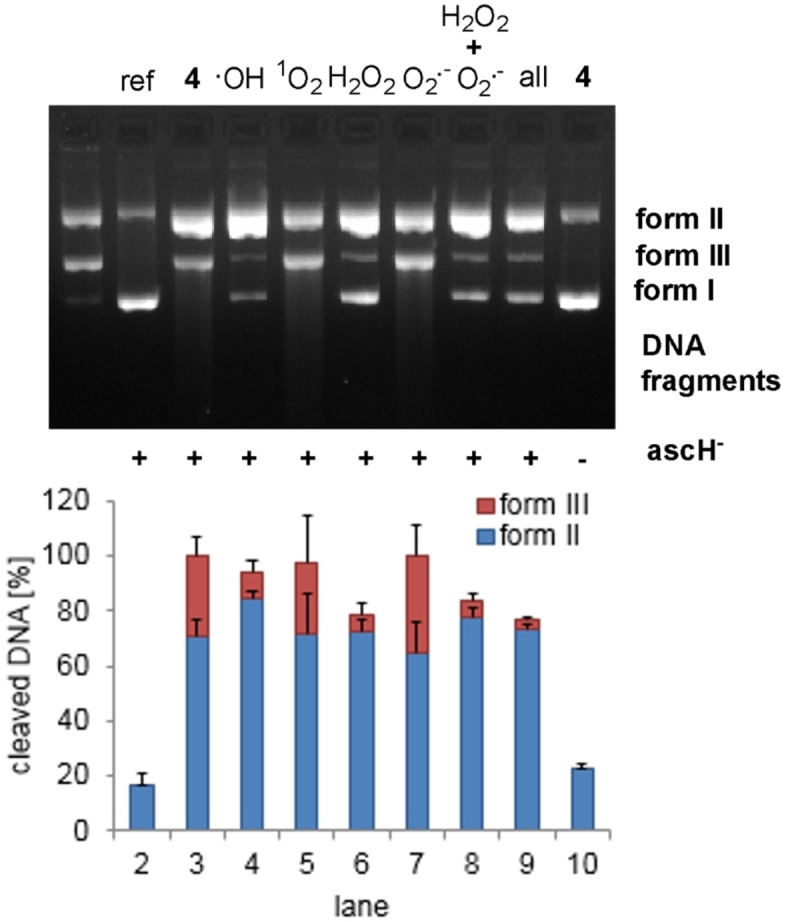
(Top) Cleavage of plasmid DNA pBR322 (0.2 μg) by complex **4** (35 μM) in MOPS buffer (50 mM, pH 7.4) in the presence of ascH^−^ (1 mM). Incubation for 1 h at 37 °C in the absence and presence of corresponding ROS scavengers. Lane 1: DNA ladder (form I, II and III), lane 2: DNA reference, lane 3: **4**, lanes 4–9: **4** and scavengers for the indicated ROS (DMSO (400 mM), NaN_3_ (10 mM), pyruvic acid (2.5 mM), SOD (625 U/mL), pyruvic acid (2.5 mM)+SOD (625 U/mL), all scavengers), lane 10: **4** without ascH^−^. (Bottom) Visualization of the extent of DNA cleavage in percent. Error bars represent the standard deviation from at least three experiments.

In order to prove an oxidative DNA cleavage mechanism of complexes **1**–**7**, a commonly used ROS quenching assay by means of gel electrophoresis^[40]^ was carried out exemplarily for the most potent DNA cleaving agent, **4**. A suitable concentration at which DNA forms II and III are visible, was determined to be 35 μM through a concentration‐dependent DNA cleavage experiment (S‐6.2, 0–50 μM). In Figure 4 the quenching of ROS in the presence of different scavengers (DMSO for ^•^OH,^[41]^ NaN_3_ for ^1^O_2_,^[40]^ pyruvate for H_2_O_2_ ^[42]^ and SOD for O_2_^.−[40]^) is shown.

Complex **4** cleaved plasmid DNA in the presence of ascH^−^ to 70 % into form II and 30 % into form III (Figure 4, lane 3). No change in the cleavage activity was observed by addition of the ROS scavengers NaN_3_ and SOD (lanes 5 and 7). Thus, ^1^O_2_ and O_2_^.−^ are presumably not involved in the DNA cleavage. In contrast, DMSO and pyruvic acid significantly quenched the nuclease activity (lanes 4 and 6), thus hydroxyl radicals and hydrogen peroxide are suggested to be responsible for DNA damage by complex **4**. An oxidative DNA cleavage mechanism is also supported by the fact that hydrolytic cleavage can be excluded as complex **4** does not show any DNA cleavage in the absence of ascH^−^ (lane 10).[^6^, ^7^]

**Scheme 1:**
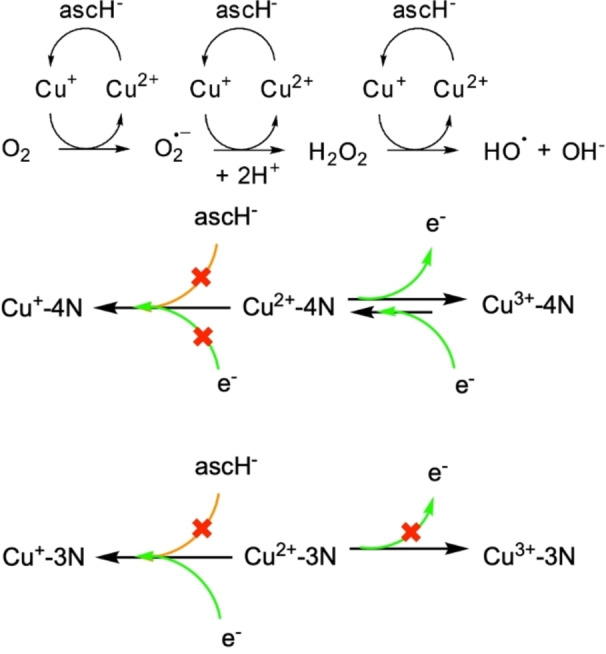
(Top) Commonly accepted ROS generation cycle for Cu(II) complexes.^[43]^ (Middle) Limited chemical (orange lines) and electrochemical (green lines) accessibility of the reduction and oxidation pathways for Cu(II) with a 4N chelating ligand (NH_2_‐aa1‐aa2‐His: ATCUN 5,5,6 chelates), and (bottom) with an analogous 3N chelating system (NH_2_‐aa1‐His: 5,6 chelates). aa1 and aa2 are any α‐amino acid except for Pro.^[14]^

As mentioned above, current research has shown, that it is not clear whether the Cu(II)/Cu(I) or the Cu(II)/Cu(III) redox couple is responsible for inducing ROS generation. Probably the most favorable pathway is achieved through reduction to Cu(I), also because it is doubtful to claim an oxidation to Cu(III) under reducing conditions^[14]^ (except for GGH, where COO^‐^ at the C terminus could play a role as a non‐innocent ligand^[29]^). In Scheme 1 (top), the commonly accepted ROS generation pathway for Cu(II) complexes^[43]^ is shown. Moreover, the limited chemical and electrochemical pathway accessibility for Cu(II) with a chelating 4N ligand (ATCUN motif; Gly2; 5,5,6) and with an analogous 3N chelating system (NH_2_−aa1−His; aa1=any α‐amino acid except for Pro) are depicted (middle and bottom, respectively, of Scheme 1).^[14]^

Recently, Faller et al. demonstrated low catalytic ROS generation for Cu(II) Gly2−ATCUN complexes compared to other DNA cleaving agents, such as [Cu(phen)_2_]^2+^.^[23]^ It can thus be assumed that Cu(II) Gly2−ATCUN does not follow the generally described ROS generation pathway for Cu(II) complexes (Scheme 1, top).^[43]^ This proposal is reaffirmed by the fact that Cu(II) Gly2−ATCUN complexes are electrochemically not reducible down to −1.0 V vs. NHE.[^14^, ^19^] Thus, Cu(II) reduction by ascH^−^ might be biologically inaccessible (Scheme 1, middle and bottom).[^14^, ^19^, ^20^] Additionally, the high complex stability of Cu(II) Gly2−ATCUN does not allow pronounced flexibility in the coordination geometry. Results from electrochemistry were corroborated by Faller et al. with kinetic experiments for the ^•^OH production by Cu(II) Gly2−ATCUN in the presence of ascH^−^: 7‐Hydroxycoumarin‐3‐carboxylic acid (CCA) reacts with ^•^OH to form the fluorescent product HO‐CCA. In the case of Cu(II) Gly2−ATCUN complexes no significant fluorescence signal related to ^•^OH production was observed.^[23]^

**Figure 5:**
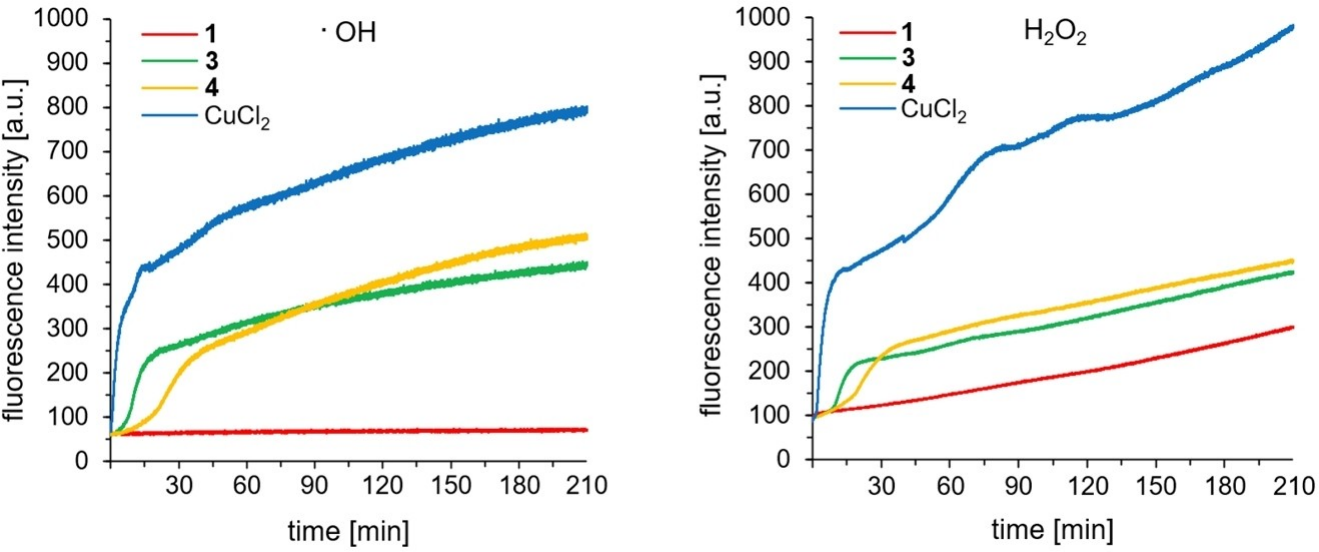
Kinetics of ^•^OH (left) and H_2_O_2_ (right) production for **1**, **3**, **4** and CuCl_2_ (40 μM) in the presence of ascH^−^ (1 mM) in MOPS buffer (50 mM, pH 7.4) monitored by fluorescence evolution of HO‐TPA (*λ*_ex_ 320 nm, *λ*_em_=428 nm) from TPA (0.5 mM), and fluorescein (*λ*_ex_=485 nm, *λ*_em_=513 nm) from PBSF (25 μM).

To similarly confirm increased DNA cleavage as a result of enhanced ROS formation when introducing β‐Ala2 in the ATCUN motif, the ^•^OH and H_2_O_2_ production was monitored. To follow the evolution of these species, the oxidation of terephthalate (TPA → HO‐TPA) by ^•^OH^[40]^ and the perhydrolysis of pentafluorobenzenesulfonyl fluorescein (PBSF) into fluorescein by H_2_O_2_ ^[40]^ were analyzed by fluorescence spectroscopy. In Figure 5, the kinetics of ^•^OH and H_2_O_2_ production by complexes **1** (Gly2), **3** (β‐Ala2), **4** (β‐Ala2; Lys1/4) and CuCl_2_ are depicted.

Indeed, low catalytic ROS generation (no ^•^OH and low H_2_O_2_ evolution) arose from Cu(II) Gly2−ATCUN complex **1** (Figure 5) in accordance with the above mentioned fluorescent HO−CCA experiments by Faller et al.^[23]^ Through an exchange of Gly2 (**1**) with β‐Ala2 (**3** and **4**) in the ATCUN motif the catalytic activity was drastically increased (Figure 5), which explains the higher nuclease activity (see preceding section), probably through an easier access of the conventional Cu(II) cycle for ROS generation (Scheme 1, above). This is corroborated by the fact that β‐Ala2 complexes **3**–**7** are able to form Cu(II)−2N complexes (terminal NH_2_ and N_im_) more easily than Gly2 complexes **1** and **2** (see section “Stability constants of Cu(II) complexes at pH 7.4” and S‐5.2). Although Cu(II)‐2N species are unambiguously only observed at pH values around 4–6, this shows the flexibility of the coordination geometry of β‐Ala2−ATCUN motifs around the metal center, and also explains the ease of access to tetrahedral Cu(I) coordination upon reduction.

In order to prove that ROS formation of β‐Ala2 complexes **3**–**7** is not caused by released Cu(I) upon reduction, the association constant towards Cu(I) of Gly2 peptide **a** and β‐Ala2 peptide **c** were exemplarily determined (*K*_app_=5.50±0.92×10^6^ M^−1^ and 1.78±0.31×10^6^ M^−1^ for reduced **1** and **3**, respectively; see S‐5.1 and S‐5.3). Both *K*_app_ values are in the range of those of Cu(I) complexes of several amyloid‐β peptides.^[44]^ Although Cu(I) affinity of the β‐Ala2 peptide **c** is lower than that of Gly2 peptide **a**, it is still in the same order of magnitude, and thus corroborates the ROS formation pathway through the Cu(II)/Cu(I) redox cycle for β‐Ala2 complexes.

Complex **3** (β‐Ala2) and **4** (β‐Ala2; Lys1/4) exhibited very similar kinetic profiles of ^•^OH and H_2_O_2_ production (Figure 5). Since their DNA cleavage activity is different (Figure 3), deviations in DNA interactions within the series of β‐Ala2 complexes **3**–**7** (Lys and Trp) are suggested to be responsible for the contrasting nuclease activities (see above). We have shown in the past that the combination of redox and DNA binding properties is eventually responsible for the observed DNA cleavage activity.^[30]^

The highest ^•^OH and H_2_O_2_ formation was observed in the case of an aqueous CuCl_2_ solution. In this case, the ROS generation occurs presumably near to TPA and PBSF, while the steric hindrance of peptide ligands prevents the formation of short‐lived ROS^[45]^ in close proximity needed for hydroxylation or perhydrolysis. Additionally, we demonstrated in separate experiments that the fluorescence signals (HO‐TPA and fluorescein) are caused by metallonuclease‐generated ^•^OH and H_2_O_2_ through signal quenching with their corresponding ROS scavengers DMSO and pyruvic acid (Figures S64 and S65).

### Cyclic voltammetry

**Figure 6:**
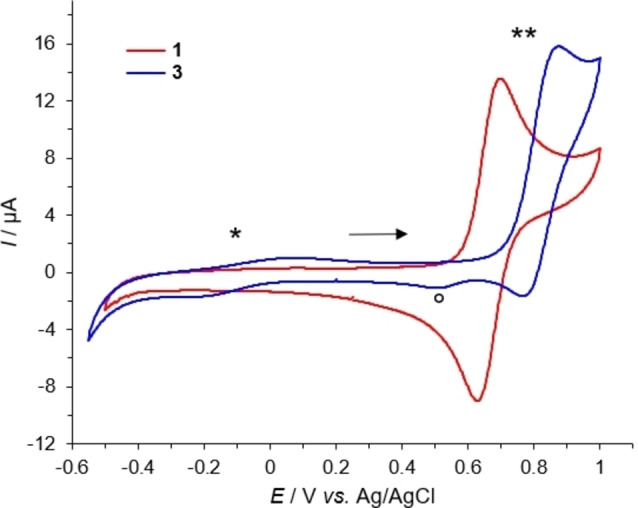
Cyclic voltammograms obtained for 0.5 mM of complex **1** (Gly2; 5,5,6) and **3** (β‐Ala2; 5,6,6) at pH 7.4 recorded in 96 mM KNO_3_/4 mM HNO_3_. * indicates the Cu(II)/Cu(I) reduction process for **3**, ** the quasi‐reversible Cu(II)/Cu(III) oxidation process for both **1** and **3**, and ° an additional reduction for **3** at around *E*_1/2_=+0.51 V related to the arrangement of two adjacent 6‐membered chelates (5,6,6).^[19]^ The arrow indicates the starting point and direction of the potential scanning. The scan rate was 100 mV/s.

To underpin our proposal for a mechanism, we carried out cyclic voltammetric and EPR spectroscopic experiments with the Gly2 complexes Cu−GGH and **1** and the β‐Ala2 complexes **3** and **4** to get a better insight into how the ROS generation is associated with redox and coordination chemistry. Exemplarily, in Figure 6 the cyclic voltammograms of **1** and **3** are shown (Figure S66 for Cu‐GGH and **4**).

For both complexes **1** and **3**, a quasi‐reversible Cu(II)/Cu(III) redox couple was observed at *E*_1/2_=+0.67 V and +0.83 V against Ag/AgCl, respectively. More interestingly, for the β‐Ala2 complex **3** a small peak assignable to the Cu(II)/Cu(I) reduction process is visible at around *E*_1/2_=−0.07 V, which is absent in the cyclic voltammogram of complex **1**. Although the reduction to Cu(I) is only observable if Cu(II) is initially oxidized to Cu(III) (anodic direction first),^[19]^ it shows that it is more easily accessible for a more flexible ligand scaffold, as for Cu−3N complexes (Scheme 1, bottom). This is in accordance with previous results which indicated that an increase in ring size of the chelate results in an anodic shift of *E*_1/2_.[^31^, ^46^]

### EPR spectroscopy and DFT

**Figure 7:**
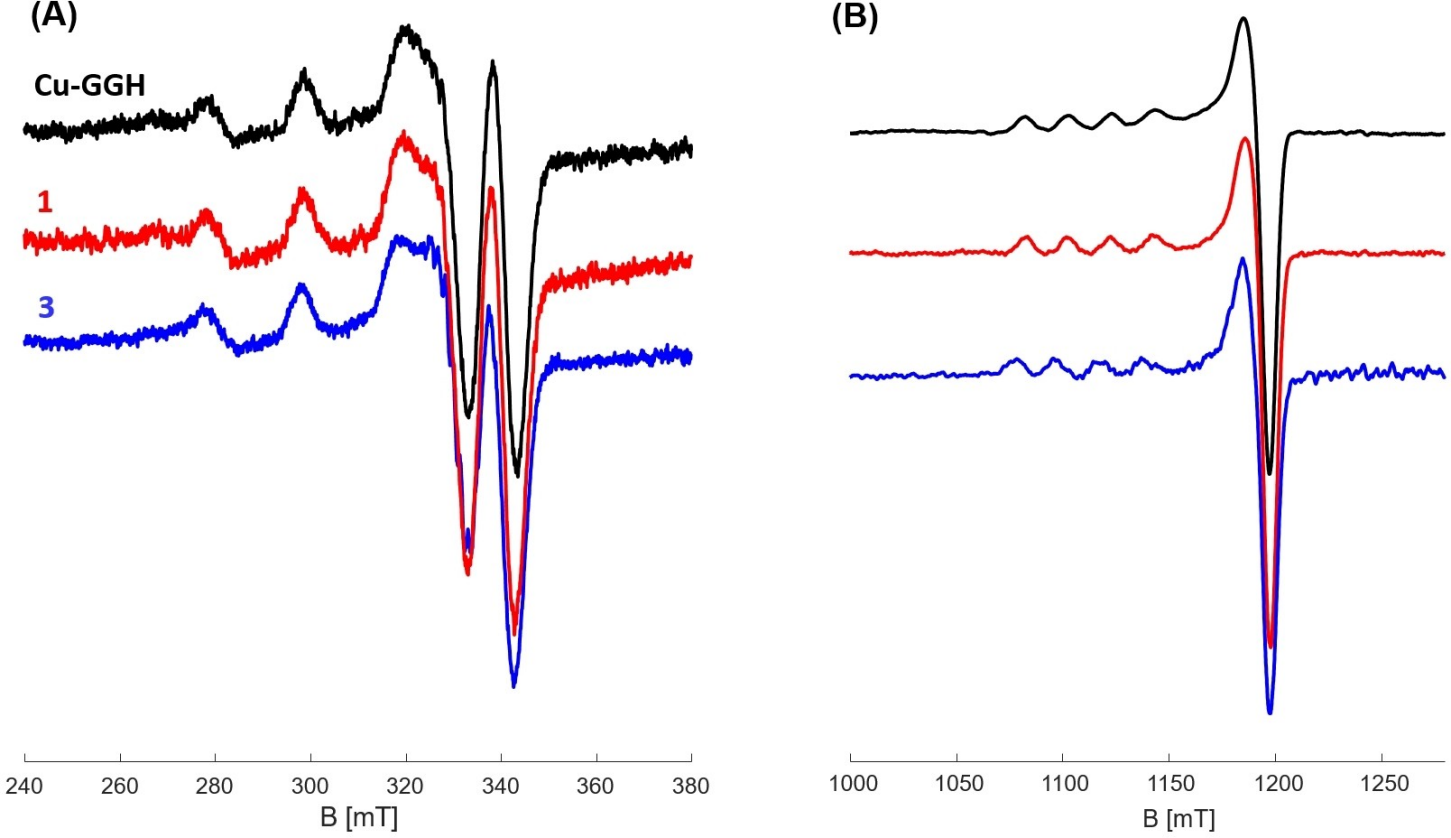
Low‐temperature CW‐EPR spectra for Cu−GGH, **1** and **3** at (A) 9.4 GHz (X‐band), 77 K and (B) 34 GHz (Q‐band), 50 K.

In order to characterize the coordination environment and electronic structure of the Cu(II) complexes in solution, X‐ and Q‐band EPR measurements were performed, and independently investigated by DFT studies. In Figure 7 low‐temperature CW‐EPR spectra of Gly2 complexes Cu−GGH and **1**, and β‐Ala2 complex **3** at 9.4 GHz (X‐band) and 34 GHz (Q‐band) are shown (see S‐10 for experimental details and spectral simulations). The corresponding g‐ and hyperfine coupling, A, tensor elements are listed in Table 2.

**Table 2:** Spin‐Hamiltonian parameters (principal elements of g and hyperfine coupling, A, tensors) of Gly2 complexes Cu−GGH and **1** and β‐Ala2 complex **3** at 9.4 GHz (X‐band) and 34 GHz (Q‐band) based on spectral simulations (S‐10) and DFT calculations.

Complex	exp. X‐band		exp. Q‐band		calc. (DFT)	
	g_x,_ g_y_, g_z_	A_x_, A_y_, A_z_ [MHz]	g_x_, g_y,_ g_z_	A_x_, A_y_, A_z_ [MHz]	g_x_, g_y_, g_z_	a_iso_ (A’_dip_) [MHz]
Cu−GGH	2.048, 2.048, 2.200	30, 100, 580	2.048, 2.048, 2.200	70, 70, 560	2.05, 2.07, 2.20	156 (−407, +176, +230)
**1**	2.048, 2.048, 2.200	30, 60, 580	2.048, 2.048, 2.199	70, 70, 560	2.06, 2.06, 2.20	151 (+221, +228, −449)
**3**	2.048, 2.048, 2.200	30, 100, 580	2.048, 2.048, 2.211	70, 70, 560	2.06, 2.07, 2.22	174 (+205, +243, −448)

At Q‐band frequency, the EPR spectra of all complexes clearly display a typical axial symmetry for both g‐ and A‐tensors, indicating the unpaired electron resides in the 3d _x2‐y2_ singly occupied molecular orbital (SOMO). These spectral features thus suggest an octahedral, elongated octahedral or square pyramidal coordination for the CuN_4_ system. In contrast to axial symmetry (g and A tensors) at Q‐band, at lower X‐band frequency we observe for all complexes an anisotropic (rhombic) A tensor indicating a distorted symmetry of the ATCUN ligand coordinated to Cu(II), confirmed by spectral simulations (S‐10).[^47^, ^48^]

Furthermore, for the β‐Ala2 complex **3** a slightly higher g_z_ value at Q‐band was found (2.211) compared to Cu−GGH and **1** (2.200 and 2.199), which is assigned to different positioning of axial ligands (see DFT results below). It is known, that if g_z_ is increasing and A_z_ is decreasing, the tetrahedral distortion within a CuN_4_ system is enhanced.[^48^, ^49^] Here, A_z_ does not not change, however, a slight increase of g_z_ of β‐Ala2 complex **3** could be an indication of a more pronounced tetrahedral distortion which may facilitate the access to a tetrahedral Cu(I) coordination. This very likely results from the additional CH_2_ group and thus increased structural flexibility in the ligand scaffold.

**Figure 8:**
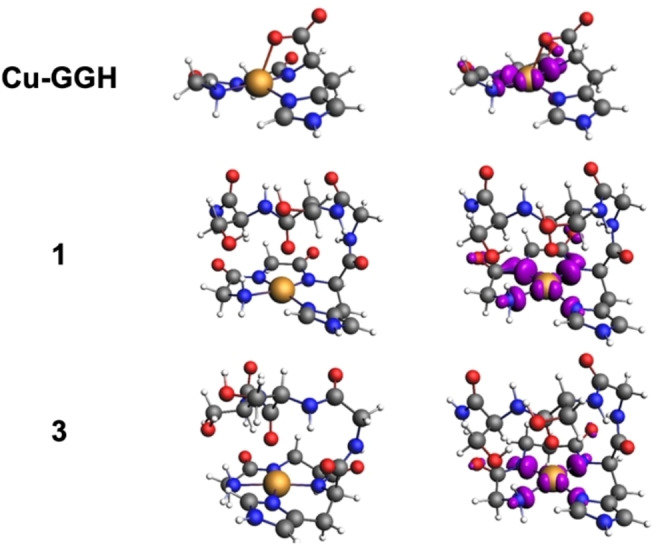
Global minima structures from conformation sampling of Cu(II) ATCUN complexes Cu−GGH, **1** and **3** and unpaired spin density at an isovalue of 0.005 e/a.u.

The plasticity and accessibility of a distorted ligand coordination environment as in the case of peptidic ligands, often make the interpretation of EPR spectra difficult. Their interpretation was thus augmented by quantum chemical calculations using the GFN2‐xTB Hamiltonian and DFT. A larger number of conformers of Cu(II) complexes are accessible at finite temperature. Figure 8 shows the top‐ranked unique entries of the conformer‐rotamer ensemble (CRE) following an exhaustive CREST (conformation‐rotamer ensemble tool) sampling for Cu−GGH, **1** and **3** plus the PBE0 calculated unpaired spin densities (see S‐11 for computational details). For axial Cu complexes, out of eleven tested functionals, in particular hybrid density functionals such as B3LYP and PBE0 were shown to perform best for the g‐tensor principal value g_z_ and the ^63^Cu A_z_ hyperfine interactions.^[50]^ These are most sensitive to changes in the coordination sphere. Table 2 shows the PBE0 calculated g‐tensor principal values and the ^63^Cu hyperfine coupling parameters (a_iso_ and A’_dip_). The calculations allow the separation of the two and resolve the signs of each.

The calculated EPR parameters are in excellent agreement with the experiment and also reproduce the slight increase in g_z_ values for complex **3** (Table 2). This corroborates the structural proposal regarding an easier access to a tetrahedral Cu(I) coordination for the β‐Ala2 complexes like **3**.

For all complexes a penta‐coordinated Cu(II) with a distorted square pyramidal coordination sphere was found with a 3d_x2‐y2_ ground state. The spin density at Cu(II) is almost independent of the nature of ATCUN ligands which explains the similarity of g‐ and A‐tensor values in all complexes.

Additionally, in the CREST searches an increase of thermally accessible unique structures in the CRE was found from 110 for Gly2 complex **1** to 156 in β‐Ala2 complex **3**, which suggests in accordance to the results described above a more flexible chelating ligand scaffold in **3** due to the additional CH_2_ group. The calculated EPR parameters are almost independent of the chosen conformer for each complex due to the conserved 4N coordination environment (more results for representatives of the CRE in Tables S10–S12).

In **1** and **3**, the 4N equatorial coordination is complemented by an additional axial amide oxygen. For Cu‐GGH, three different structures appear possible (Table S10). From the corresponding calculated EPR parameters, the ones of a penta‐coordinated Cu(II) are in best agreement with the experiment. The axial fifth ligand may either be an intramolecular coordination by the terminal carboxylate group (Cu⋅⋅⋅^−^OOC distance of 2.30 Å) or an external water molecule at 2.80 Å.

### Antiproliferative activity in cancer cells

The antiproliferative activity of Cu(II) ATCUN complexes Cu−GGH, **1**, **3** and **4**, and CuCl_2_ towards the human cancer cell lines HCT116 (colorectal carcinoma), NCI‐H460 (non‐small cell lung carcinoma), SiHa (cervical carcinoma) and SW480 (colon adenocarcinoma) were determined by the sulforhodamine B (SRB) assay (Table S13).

The peptides GGH, **a**, **c** and **d** (not shown) and CuCl_2_ itself are non‐toxic against the chosen cancer cell lines within the concentration range used (IC_50_ >70 μM). While both Gly2 complexes Cu−GGH and **1** were not cytotoxic, the β‐Ala2 complexes (**3**, **4**) showed moderate antiproliferative activity against HCT116 and NCI‐H460 cancer cells with IC_50_ values around 60 μM. Although these IC_50_ values are too high to be pharmacologically relevant, it is noteworthy that **3** and **4** showed greater activity as anticancer agents, which reflects their higher activity as ROS‐inducing and DNA cleaving agents. For the observed moderate activity, it should be considered that strong Cu(I) chelators in cells (MTs, GSH), could hamper the ROS generation via a Cu(I) pathway.[^14^, ^23^, ^51^, ^52^]

We aimed to correlate the antiproliferative activity to the accumulation of Cu in HCT116 cells. Therefore, cells were treated for 24 h with complexes Cu−GGH, **1**, **3**, **4** and CuCl_2_ (70 μM), and the Cu content of the samples was determined by ICP‐MS (Table S13). The less cytotoxic complexes Cu−GGH and **1** showed low cellular uptake (∼0.3 μg Cu/4×10^5^ cells). In contrast, treatment with the more cytotoxic β‐Ala2 complexes **3** and **4** resulted in higher accumulated Cu contents (∼0.75 μg/4×10^5^ cells). The higher Cu contents probably lead to enhanced oxidative stress levels in cells, which consequently results in higher antiproliferative activity. Interestingly, the incorporation of positively charged Lys into the ATCUN motif (**4**) did not affect the Cu uptake in the studied cancer cell line.

CuCl_2_ treatment resulted in similar Cu levels in HCT116 cells as found for **3** and **4**. This could imply that in the case of β‐Ala2 complexes, Cu(II) and ATCUN peptides are internalized separately into the cell. The up to 1000‐fold lower complex stability of β‐Ala2 complexes also suggests this pathway. Due to the decreased complex stability intracellular ligands most probably compete with β‐Ala2 peptides for Cu(II) or Cu(I) coordination.[^23^, ^51^, ^53^] Nevertheless, CuCl_2_ and the peptides alone are not cytotoxic, and only Cu(II) in combination with the β‐Ala2 ATCUN peptides are active.

## Conclusion

Since the discovery of the highly potent Cu(II) complex Cu−GGH, the ATCUN motif yielded complexes with rather low cytotoxic activity.[^54^, ^55^] Furthermore, such complexes were attested low catalytic ROS generation.[^14^, ^23^, ^51^] Both aspects have led to neglecting the ATCUN motif as a promising candidate for clinical application in chemotherapy.

In this work, we demonstrated that with the exchange of the α‐amino acid Gly with the β‐amino acid β‐Ala in position 2 of Cu(II) ATCUN complexes, thus by changing the chelate ring sizes (5→6), a significant increase in the DNA cleavage and ROS evolution was observed. These effects were mirrored by moderate antiproliferative activity in cancer cells, while Gly‐containing complexes showed no cytotoxicity. This finding could play a key role in the successful design of novel ATCUN‐based chemotherapeutic agents. Considering that Cu is an endogenous metal fewer side effects than with for example Pt‐based compounds might be expected. Investigations for gaining a deeper insight into the cell death mechanism are underway.

## Conflict of interest

The authors declare no conflict of interest.

## Supporting information

As a service to our authors and readers, this journal provides supporting information supplied by the authors. Such materials are peer reviewed and may be re‐organized for online delivery, but are not copy‐edited or typeset. Technical support issues arising from supporting information (other than missing files) should be addressed to the authors.

Supporting Information
