# Supplementary material for: Incorporation of β‐Alanine in Cu(II) ATCUN Peptide Complexes Increases ROS Levels, DNA Cleavage and Antiproliferative Activity
Source: Chemistry. 2021 Dec 4;27(72):18093–102. doi: 10.1002/chem.202102601 (PMC9299640; doi:10.1002/chem.202102601)
Supplement: Supplementary file 1 — Supporting Information [file CHEM-27-18093-s001.pdf]

Chemistry–A European Journal

Supporting Information

Incorporation of β-Alanine in Cu(II) ATCUN Peptide Complexes Increases ROS Levels, DNA Cleavage and Antiproliferative Activity

Julian Heinrich, Karolina Bossak-Ahmad, Mie Riisom, Haleh H. Haeri, Tasha R. Steel, Vinja Hergl, Alexander Langhans, Corinna Schattschneider, Jannis Barrera, Stephen M. F. Jamieson, Matthias Stein, Dariush Hinderberger, Christian G. Hartinger, Wojciech Bal, and Nora Kulak*

S-1 Materials and general methods
S-2 Peptide synthesis
S-2.1 Solid-phase peptide synthesis (SPPS)
S-2.2 High performance liquid chromatography (HPLC)
S-2.3 Electrospray ionization-mass spectrometry (ESI-MS)
S-3 Determination of peptide yields
S-4 *In situ* Cu(II) complex synthesis
S-5 Protonation and complex stability constants
S-5.1 Potentiometry, UV/VIS and circular dichroism (CD) spectroscopy
S-5.2 pH-dependent species distribution of Cu(II) ATCUN complexes
S-5.3 UV/VIS titration of $[CuFz_2]^{3-}$ with peptides **a** and **c**
S-6 DNA cleavage studies
S-6.1 Nuclease activity of peptides **a–g** in the presence of ascorbate as a reducing agent
S-6.2 Nuclease activity of Cu(II) ATCUN complex **4** at different concentrations in the presence of ascorbate as a reducing agent
S-7 DNA interaction studies
S-7.1 DNA melting curves: UV/VIS spectroscopy
S-7.2 Ethidium bromide (EtBr) displacement assay: fluorescence spectroscopy
S-7.3 CD spectroscopy
S-8 Detection of hydroxyl radicals and hydrogen peroxide
S-9 Cyclic voltammetry
S-10 EPR spectroscopy
S-11 DFT calculations
S-12 Cancer cell studies
S-12.1 Determination of IC_{50} values against HCT116, NCI-H460, SiHa and SW480 tumor cells
S-12.2 Cellular uptake in HCT116 cells

S-1 Materials and general methods

All chemicals and solvents were purchased from *Sigma-Aldrich*, *Merck*, *Fisher Scientific*, *Acros Organics*, *Carl Roth*, *VWR*, *POCH S.A.* and *Carbolution Chemicals*, respectively, and were used without further purification. Copper(II) chloride ($CuCl_2$) was used as the dihydrate. Ascorbate and pyruvate components in the incubation solutions were prepared by dilution of L-ascorbic acid and pyruvic acid stock solutions. Milli-Q® water (18 MΩ·cm) was used as a solvent.

For the purification of the synthesized peptides, a *VWR Hitachi Chromaster 5000* (RP-HPLC) was used. Mass spectrometry was performed on an *Agilent 6210 ESI-TOF mass spectrometer* (flow rate 10 µL/min). MeOH and MeCN were used as solvents for diluting the aqueous stock solutions of the peptides and their Cu(II) complexes, respectively. UV/VIS spectroscopy was carried out on a *Varian Cary 100 Bio UV/VIS spectrophotometer* and circular dichroism spectroscopy on a *Jasco J-810 spectrometer* if not stated otherwise. For fluorescence spectroscopy a *Varian Cary Eclipse spectrofluorimeter* was used.

S-2 Peptide synthesis

The synthesized ATCUN peptides have the general sequence of NH_2-**aa1**-**aa2**-His-**aa4**-Ser-Ser-$CONH_2$ with **aa1**, **aa2** and **aa4** being Gly, β-Ala, Lys and/or Trp (Figure S1).

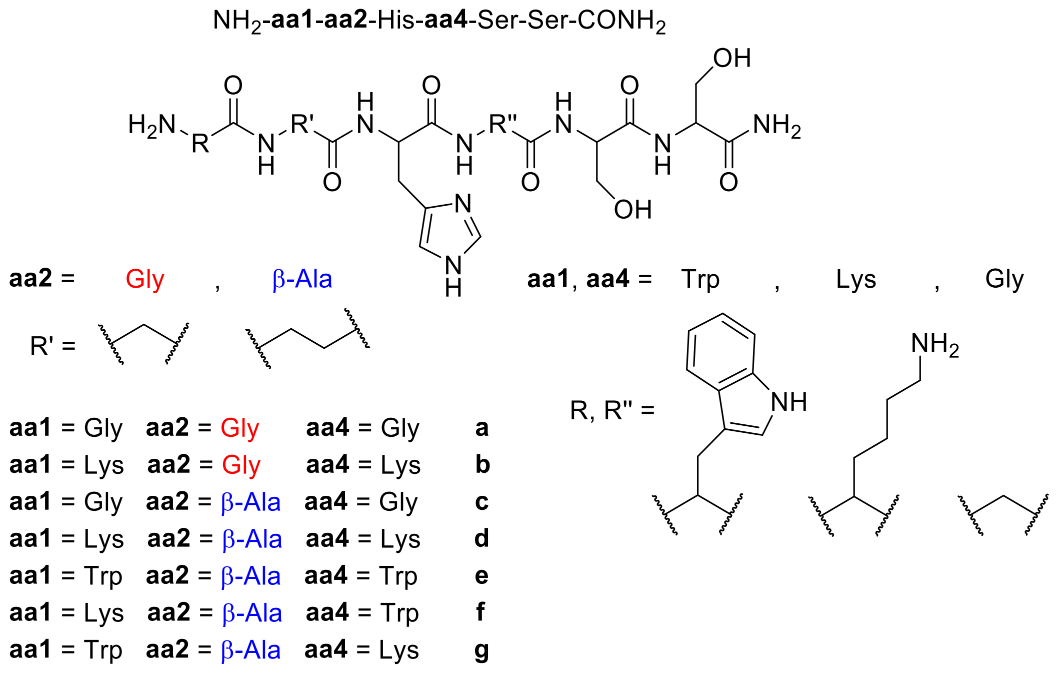

Figure S1. Synthesized ATCUN peptides with the general sequence NH_2-**aa1**-**aa2**-His-**aa4**-Ser-Ser-$CONH_2$ and variation of the first (**aa1**), second (**aa2**) and fourth (**aa4**) amino acid with Gly, β-Ala, Lys and/or Trp. The C-terminus is amidated.

S-2.1 Solid-phase peptide synthesis (SPPS)

All hexapeptides were prepared through the Fmoc strategy by using a rink amide MBHA resin as solid support. *Tert*-butyl (^{t}Bu) for serine, *tert*-butyloxycarbonyl (BOC) for Trp and Lys, and 4-methyltrityl (MTT) for His were chosen as side chain protecting groups of the amino acids. For all peptides, the corresponding L-amino acids were applied in peptide synthesis.

All synthesis steps were done in a syringe reaction vessel in DMF (*GPR Rectapure*). Rink amide MBHA resin (200 mg, 0.118 mmol) was incubated for 1 h in DMF (3 mL) for swelling. Fmoc deprotection was carried out in 20% piperidine in DMF (3 mL) for 20 min. The coupling of the amino acids was performed for 2 h with a mixture of the corresponding Fmoc-protected amino acid (0.472 mmol, 4 eq.), the activating agent PyBOP (246 mg, 0.472 mmol, 4 eq.) and *N*,*N*-diisopropylethylamine (200 µL) in DMF (3 mL). After each step of the procedure (deprotection/coupling) the solid support was washed 5 times with DMF (3 mL). An additional washing step with 5 times

dichloromethane (3 mL) was only carried out directly before cleavage of the synthesized peptides from the solid support. The cleavage of the peptides and simultaneous deprotection of the amino acid side chains was carried out for 3.5 h with a mixture of trifluoroacetic acid (TFA), triisopropylsilane (TIPS) and H_2O (90:5:5; 2.5 mL). The peptides were precipitated and washed two times with ice-cold diethyl ether.

S-2.2 High performance liquid chromatography (HPLC)

Purification of the crude peptides was performed *via* semi-preparative RP-HPLC with a C_{18} column (LiChrospher® 100 RP-18, 10 µm, 10 mm x 250 mm, *Merck*). The eluent consisted of H_2O + 0.1% TFA (solvent A) and MeOH + 0.1% TFA (solvent B).
The gradient was 5% B for 5 min, 5-50% B in 20 min, 50% B for 3 min, 50-95% B in 2 min, 95% B for 7 min, 95-5% B in 3 min and conclusively 5% B for 5 min.
The retention times (rt) of the corresponding peptides under the conditions described above were obtained by the UV/VIS-spectroscopic detection at 240 nm for **a–d** (range of peptide bond absorption band) and 300 nm for **e–g** (range of Trp absorption band). The collected fractions of peptide-trifluoroacetate salts were lyophilized, and the peptide-trifluoroacetate salts weighed for yield determination.
Due to differences in the number of protonation sites of the peptides the amount of trifluoroacetate in the eluted peptide salts differs and therefore determination of the yield just by weighing can be inaccurate. For **a**, **c** and **e**, we expect the presence of 3 trifluoroacetate counterions caused by a protonation of the terminal amine group (N-terminus), imidazole nitrogen and amide group (C-terminus). For **f** and **g** (4 trifluoroacetate anions) and for **b** and **d** (5 trifluoroacetate anions) the peptide-trifluoroacetate ratio of the salt is determined by additional protonated amine groups of the Lys side chains. A summary of the respective retention time, weight mass, molar mass of the peptide-trifluoroacetate salt and peptide yield are given in Table S1. The actual yields were determined by UV/VIS spectroscopy (see S-3).

Table S1. Retention times (rt) of the synthesized peptides during semi-preparative RP-HPLC purification with a C_{18} column, weight after drying under vacuum, molar mass of peptide trifluoroacetate salt (**a**, **c** and **e** = 3 trifluoroacetate anions, **f** and **g** = 4 trifluoroacetate anions, **b** and **d** = 5 trifluoroacetate anions) and yield of the peptides.

compound	a	b	c	d	e	f	g
rt [min]	4.25	4.49	4.45	4.53	24.06	14.63	12.79
m [mg]	58.2	55.1	41.6	89.5	50.8	12.4	25.6
M [g/mol]	841.55	1211.85	855.58	1225.87	1113.91	1169.89	1169.89
yield [%]	59	39	41	62	39	9	19

For evidence of purity, analytical RP-HPLC runs with a C_{18} column (LiChrospher® 100 RP 18, 5 µm, 3 mm x 250 mm, *Merck*) and the same gradient as in the semi-preparative runs were carried out. Due to different column conditions (semi-preparative *vs.* analytical) the rt of the purified peptides **a-g** are slightly shifted. The chromatograms of the analytical runs for each peptide **a-g** are presented in Figures S2-S8.

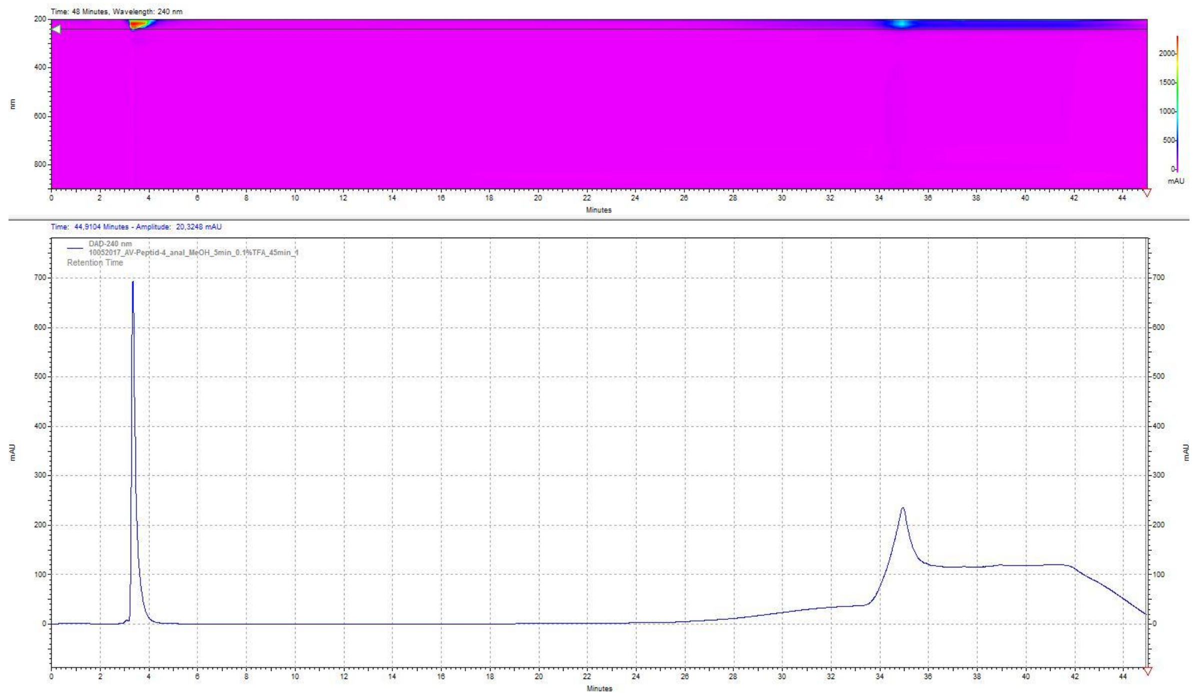

Figure S2. Chromatogram of the analytical RP-HPLC run for **a** (λ = 240 nm; same gradient as in semi-preparative purification).

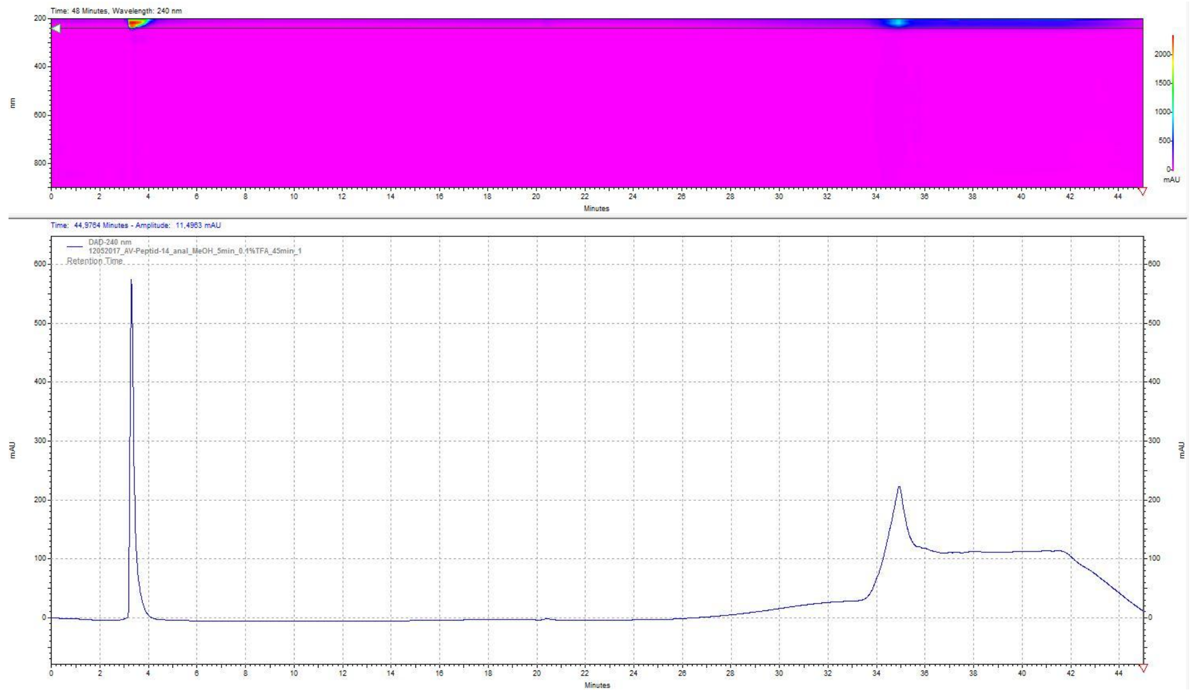

Figure S3. Chromatogram of the analytical RP-HPLC run for **b** (λ = 240 nm; same gradient as in semi-preparative purification).

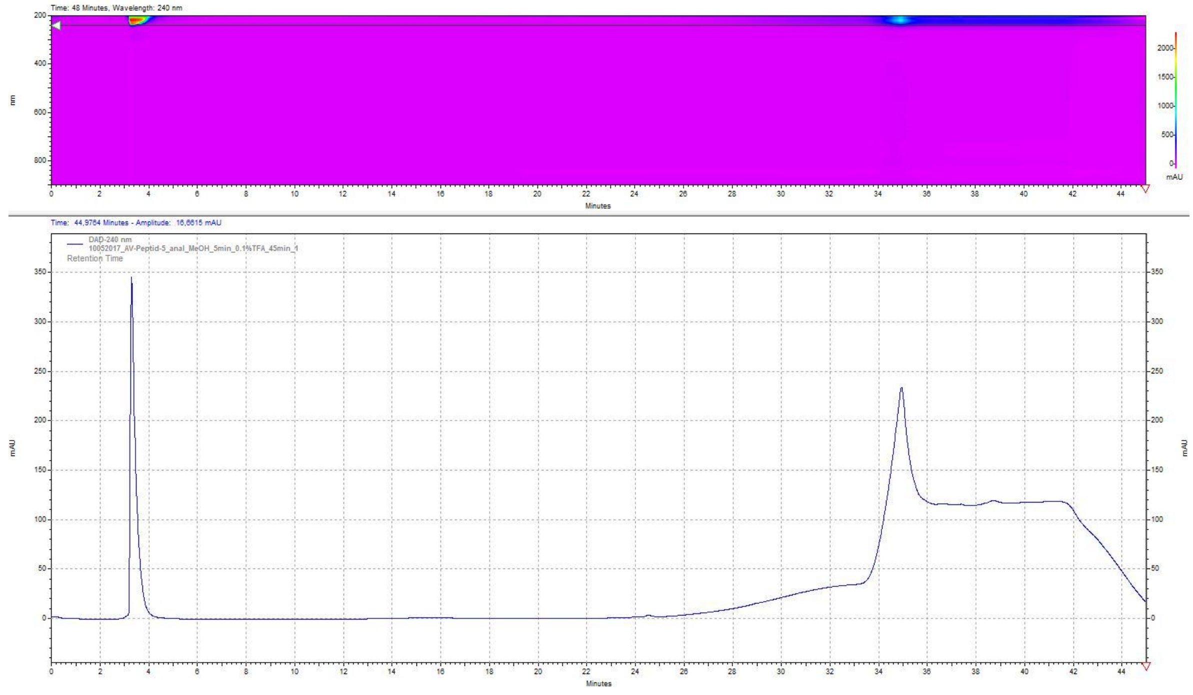

Figure S4. Chromatogram of the analytical RP-HPLC run for **c** (λ = 240 nm; same gradient as in semi-preparative purification).

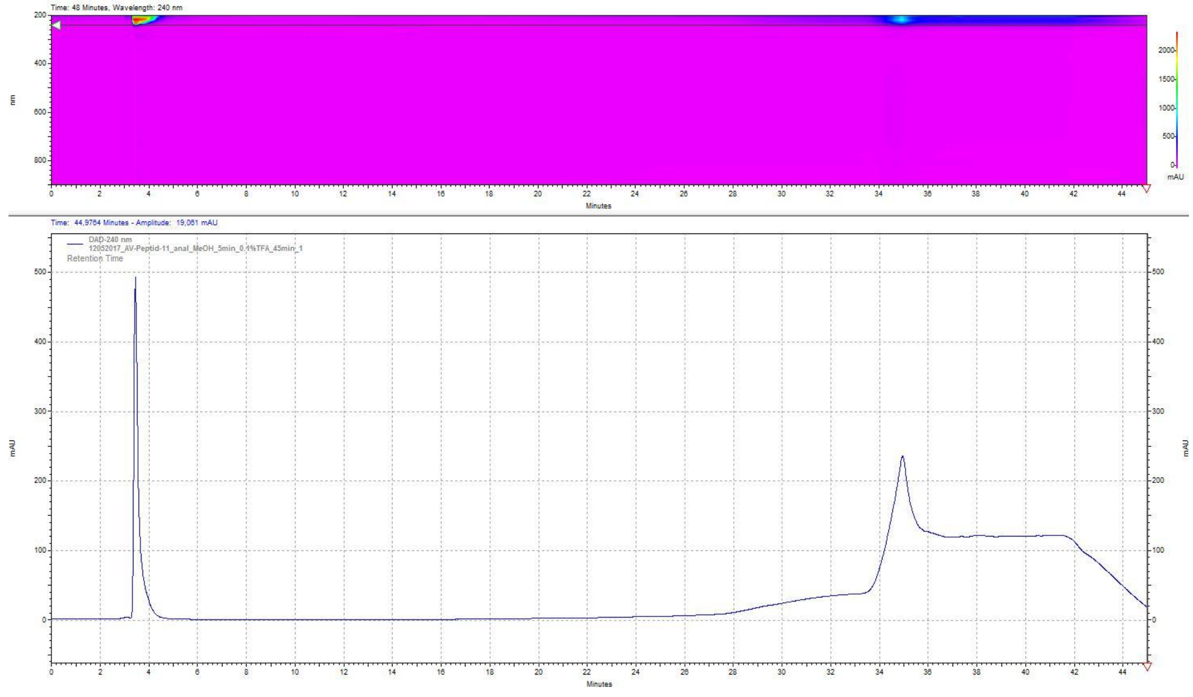

Figure S5. Chromatogram of the analytical run RP-HPLC for **d** (λ = 240 nm; same gradient as in semi-preparative purification).

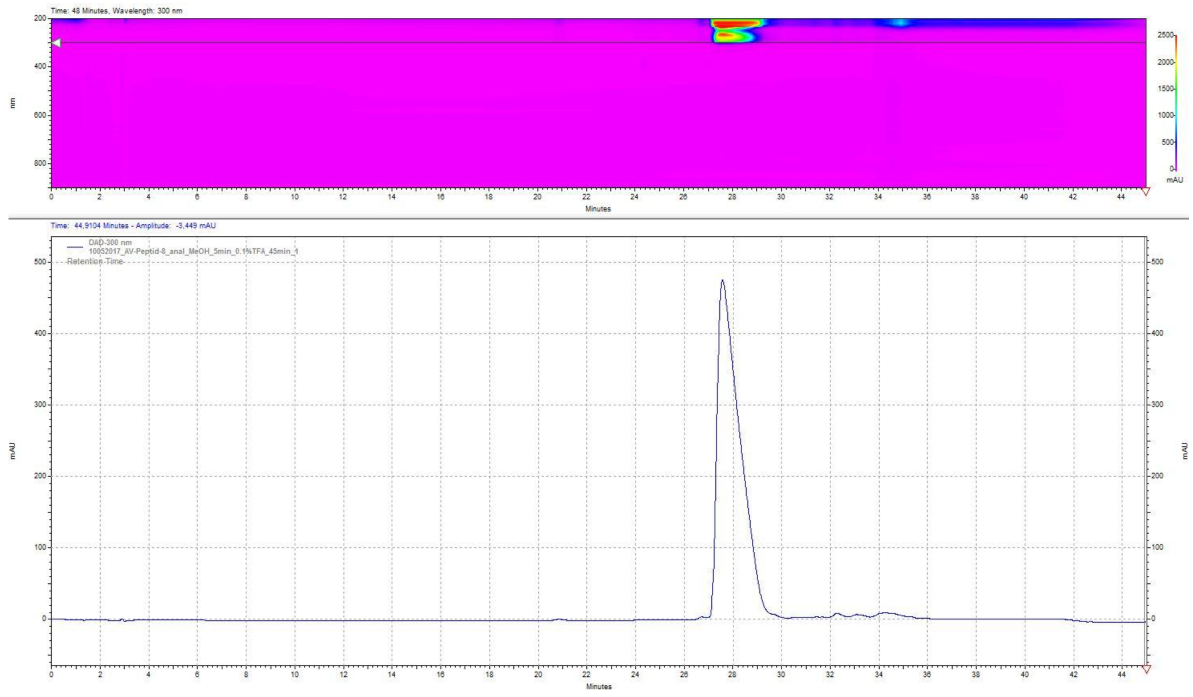

Figure S6. Chromatogram of the analytical RP-HPLC run for **e** (λ = 300 nm; same gradient as in semi-preparative purification).

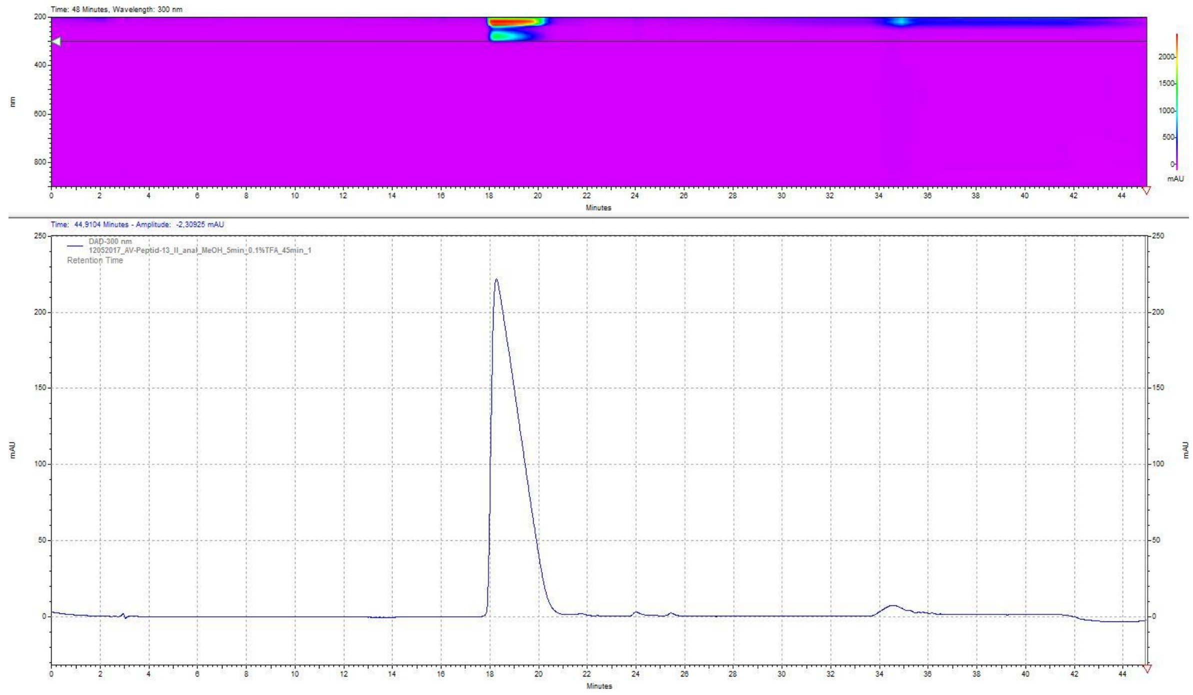

Figure S7. Chromatogram of the analytical RP-HPLC run for **f** (λ = 300 nm; same gradient as in semi-preparative purification).

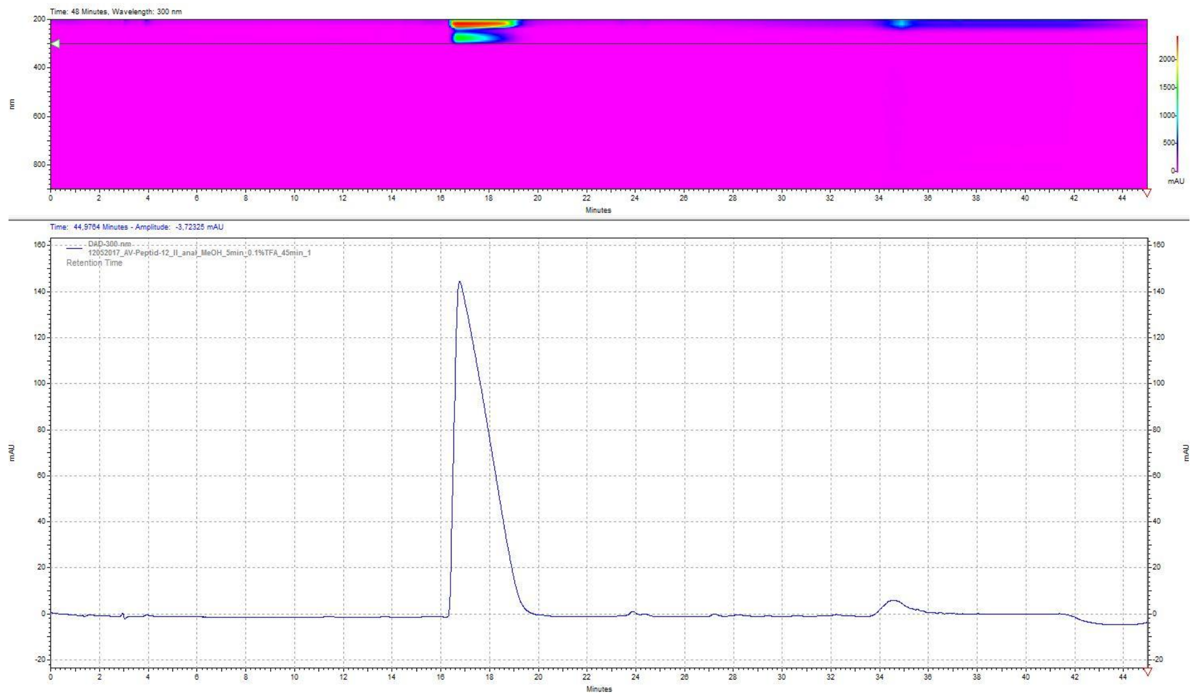

Figure S8. Chromatogram of the analytical RP-HPLC run for **g** (λ = 300 nm; same gradient as in semi-preparative purification).

S-2.3 Electrospray ionization-mass spectrometry (ESI-MS)

High-resolution ESI-MS was applied for characterization and additional purity proof of the purified peptides **a**-**g**. The calculated and found masses are listed in Table S2. The ESI mass spectra are shown in Figures S9–S15.

Table S2. Calculated and found *m/z* values in ESI mass spectra of the synthesized peptides **a**-**g** after semi-preparative RP-HPLC purification.

compound	**a**	**b**	**c**	**d**	**e**	**f**	**g**
calculated:							
$[M+H]^+$	500.2212	642.3682	514.2368	656.3838	772.3525	714.3682	714.3682
$[2M+H]^+$	999.4351	1283.7291	1027.4664	1311.7604	1543.6978	1427.7291	1427.7291
found:							
$[M+H]^+$	500.2263	642.3718	514.2385	656.3892	772.3506	714.3692	714.3653
$[2M+H]^+$	999.4410	1283.7305	1027.4683	1311.7645	1543.6933	1427.7279	1427.7242

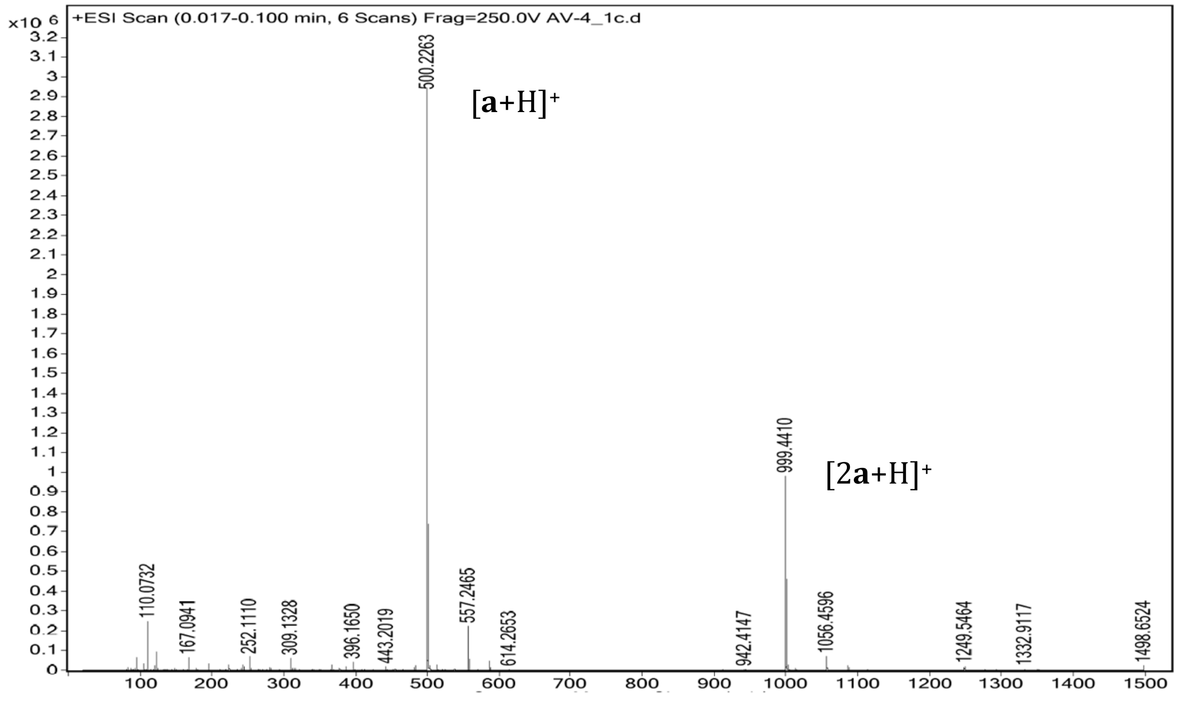

Figure S9. Mass spectrum of **a** after semi-preparative RP-HPLC purification. Dilution with MeOH.

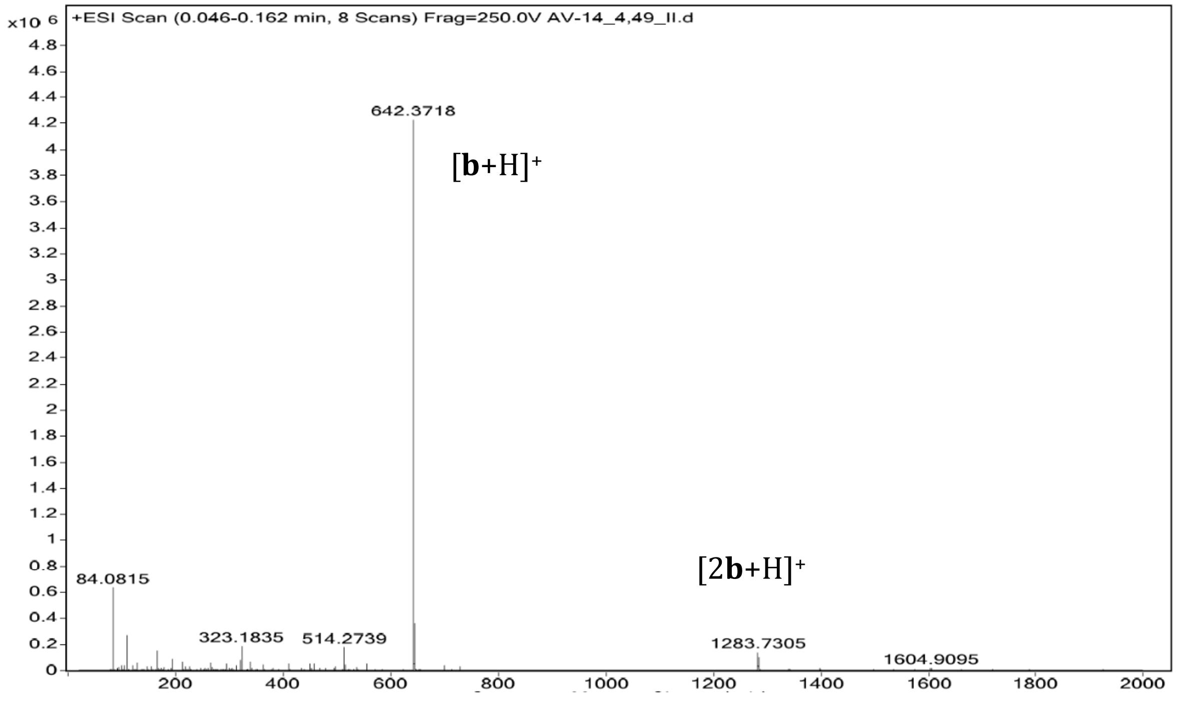

Figure S10. Mass spectrum of **b** after semi-preparative RP-HPLC purification. Dilution with MeOH.

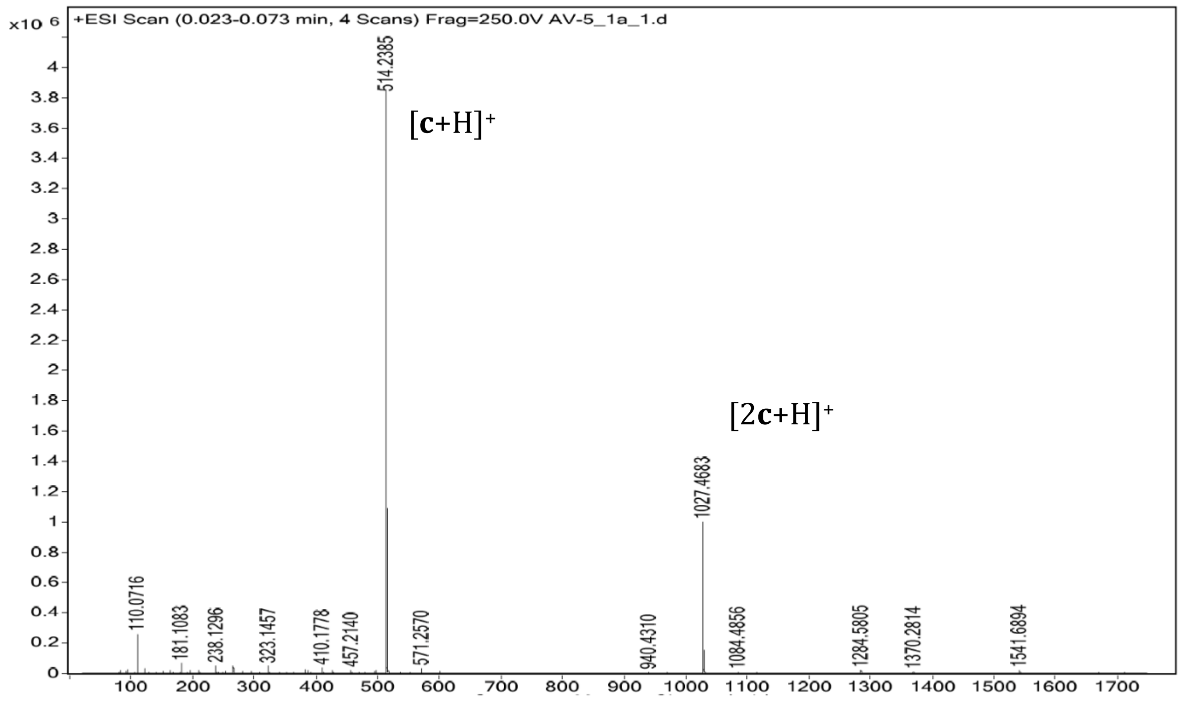

Figure S11. Mass spectrum of **c** after semi-preparative RP-HPLC purification. Dilution with MeOH.

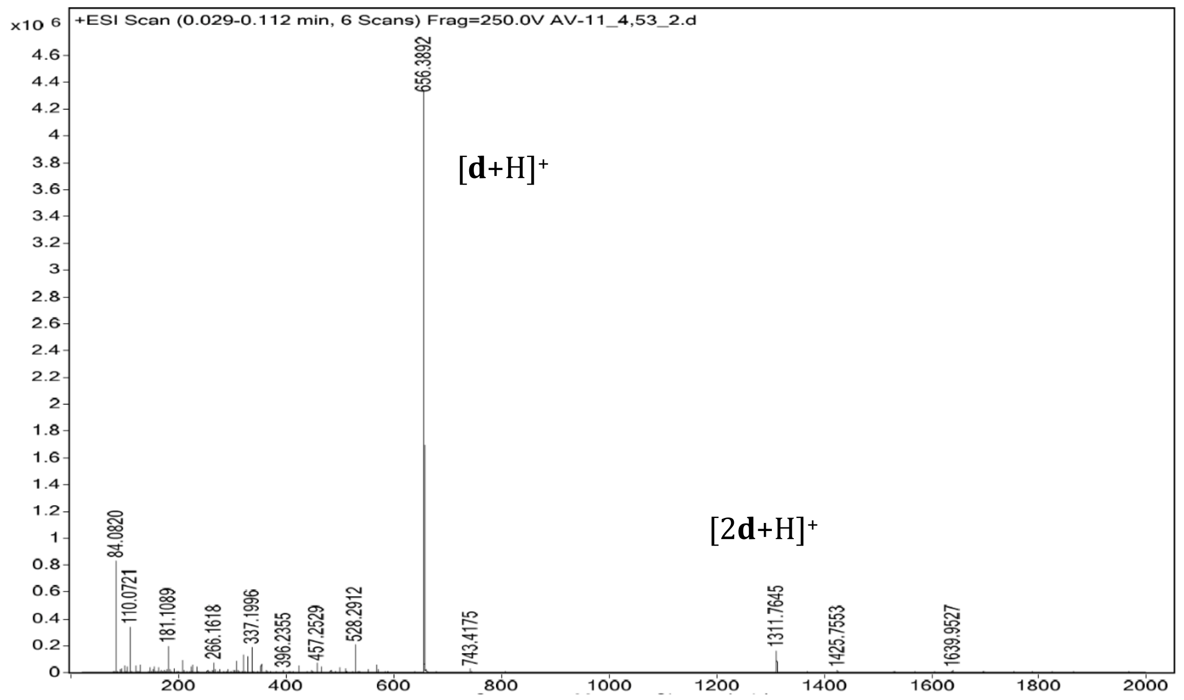

Figure S12. Mass spectrum of **d** after semi-preparative RP-HPLC purification. Dilution with MeOH.

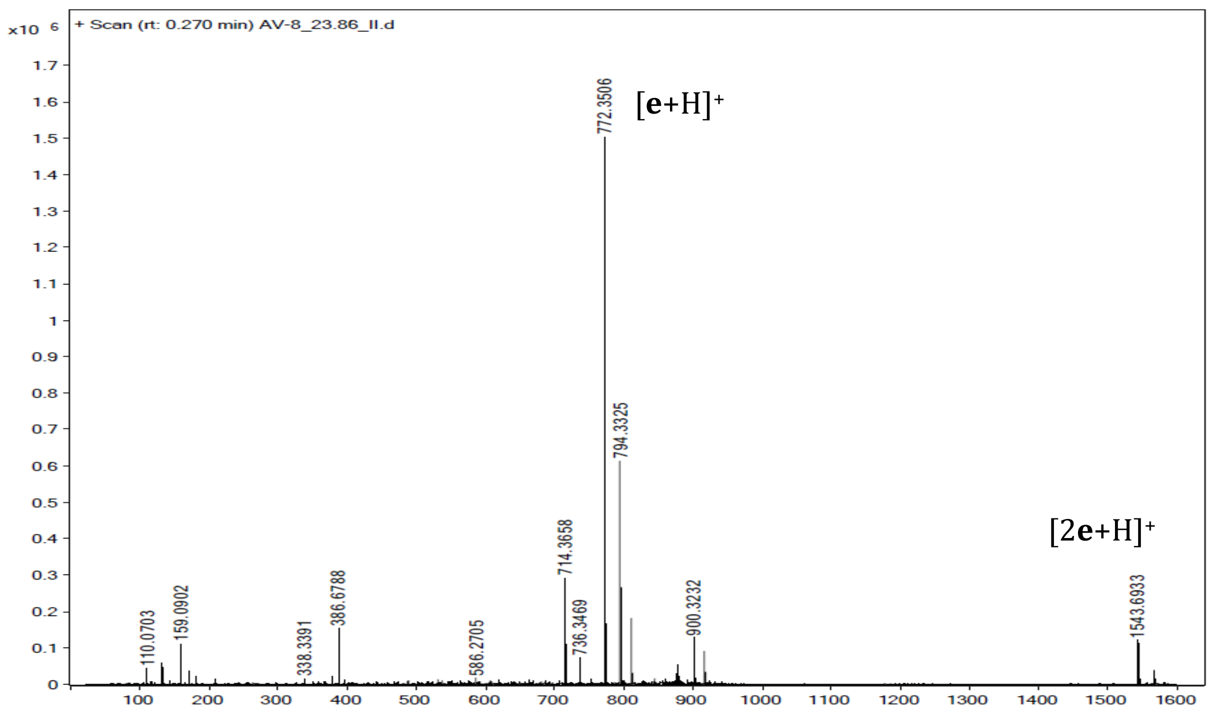

Figure S13. Mass spectrum of **e** after semi-preparative RP-HPLC purification. Dilution with MeOH.

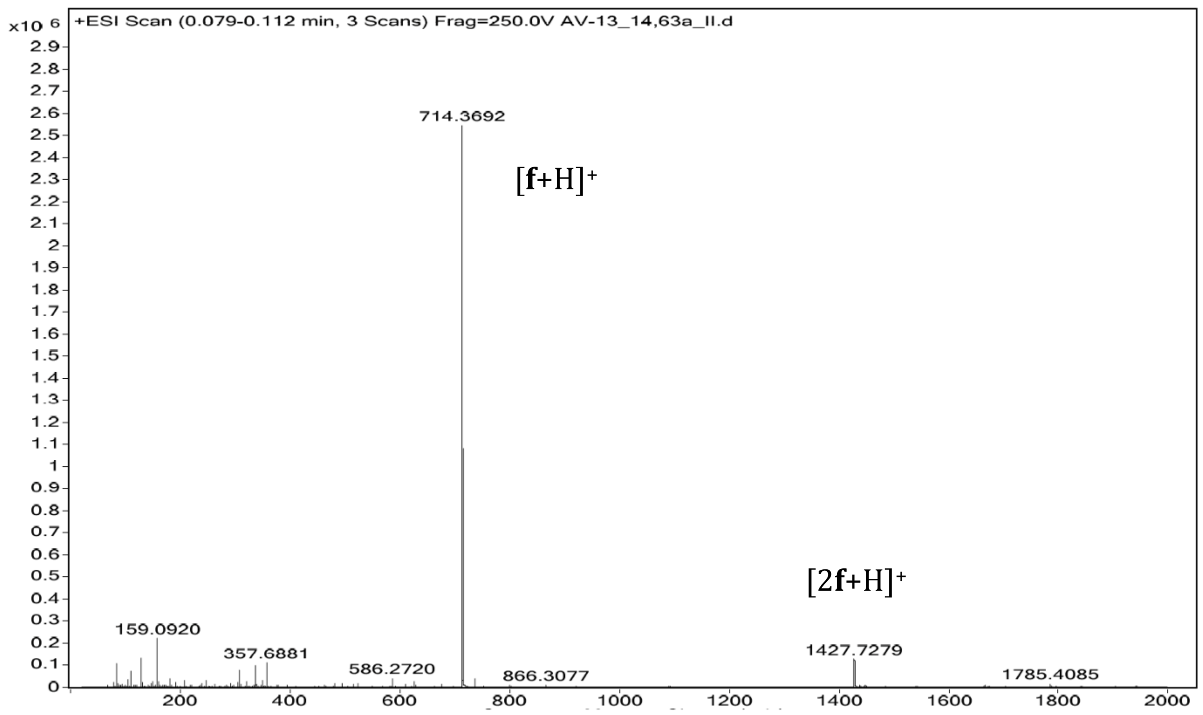

Figure S14. Mass spectrum of **f** after semi-preparative RP-HPLC purification. Dilution with MeOH.

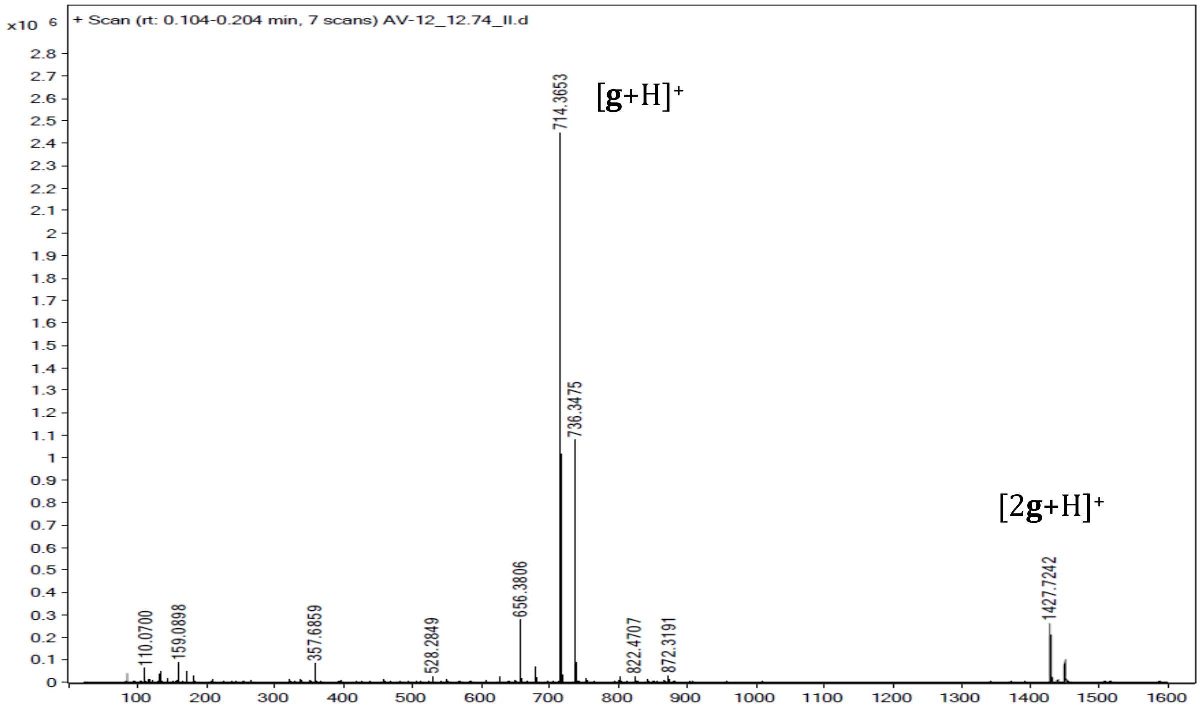

Figure S15. Mass spectrum of **g** after semi-preparative RP-HPLC purification. Dilution with MeOH.

S-3 Determination of peptide yields

The synthesized peptides **a–g** are protonated to an uncertain extent during RP-HPLC purification, resulting in an undefined composition of the peptide trifluoroacetate salt (ratio peptide : trifluoroacetate counterion). Thus, just weighing the peptide for yield determination is inaccurate. UV/VIS spectroscopy was used for adjusting the actual yield based on the known d-d transition band of Cu-GGH at around λ_{max} = 525 nm[1] (λ_{max} values of complexes **1–7** redshifted by up to 20 nm, *vide infra*).

Based on the weighing result, a 2 mM peptide solution of **a–g** (1 eq.) was prepared in 50 mM MOPS buffer (pH 7.4) and treated with $CuCl_2 \cdot 2\ H_2O$ in 0.1 mM steps (0.05 eq.). The d-d transition bands of the corresponding Cu(II) complexes **1–7** were monitored by UV/VIS spectroscopy. The saturation point of the forming d-d transition bands for each Cu(II) ATCUN complex was utilized for recalculating the actual yield. The adjusted yield for the peptides **a–g** and the absorption maxima of the d-d transition for their Cu(II) complexes **1–7** are listed in Table S3. The UV/VIS spectra in the range 400–700 nm of the Cu(II) peptide titrations are shown in Figures S16-S22.

Table S3. Yield by weighing and adjusted/actual yield of the synthesized peptides **a–g**, saturation points in equiv. of the d-d transition band detected upon treatment of the peptides with $CuCl_2 \cdot 2\ H_2O$ in 0.05 eq. steps and λ_{max} of d-d transition of the corresponding Cu(II) ATCUN complexes **1–7**.

**peptide**	**a**	**b**	**c**	**d**	**e**	**f**	**g**
**saturation point [equiv.]**	0.85	0.95	0.95	0.95	0.70	0.85	0.85
**adjusted/actual yield [%]**	50	37	39	59	27	8	16
**yield by weighing [%]**	59	39	41	62	39	9	19
**complex**	**1**	**2**	**3**	**4**	**5**	**6**	**7**
**λ_{max} of d-d transition [nm]**	529	525	545	546	529	537	541

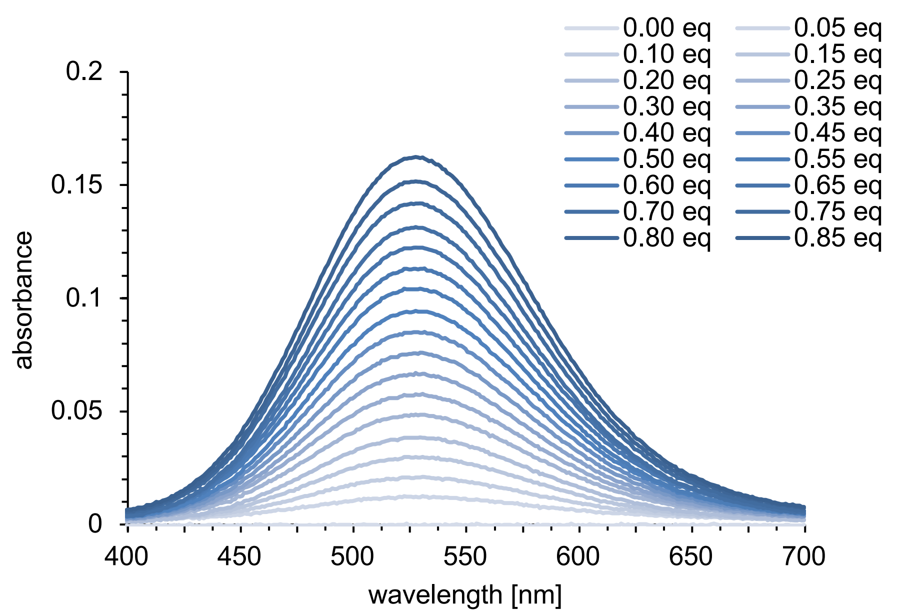

Figure S16. UV/VIS spectra in the range 400–700 nm of 2 mM peptide **a** with increasing concentrations of $CuCl_2$ (0.1 mM, 0.05 eq.) visualizing the d-d transition at λ_{max} = 529 nm of the forming Cu(II) ATCUN complex **1**. The saturation point is 0.85 eq. and was used to adjust the inaccurate peptide yield determined by weighing.

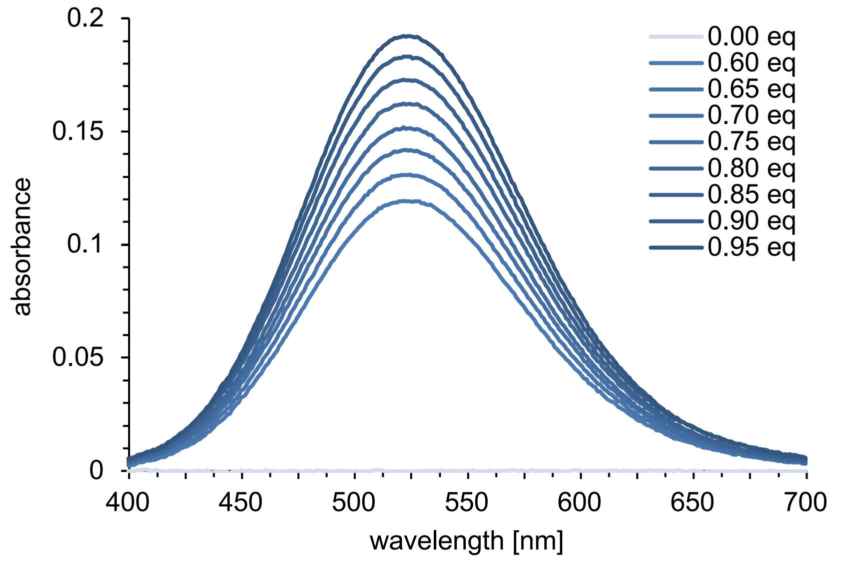

Figure S17. UV/VIS spectra in the range 400–700 nm of 2 mM peptide **b** with increasing concentrations of $CuCl_2$ (0.1 mM, 0.05 eq.) visualizing the d-d transition at λ_{max} = 525 nm of the forming Cu(II) ATCUN complex **2**. The saturation point is 0.95 eq. and was used to adjust the inaccurate peptide yield determined by weighing.

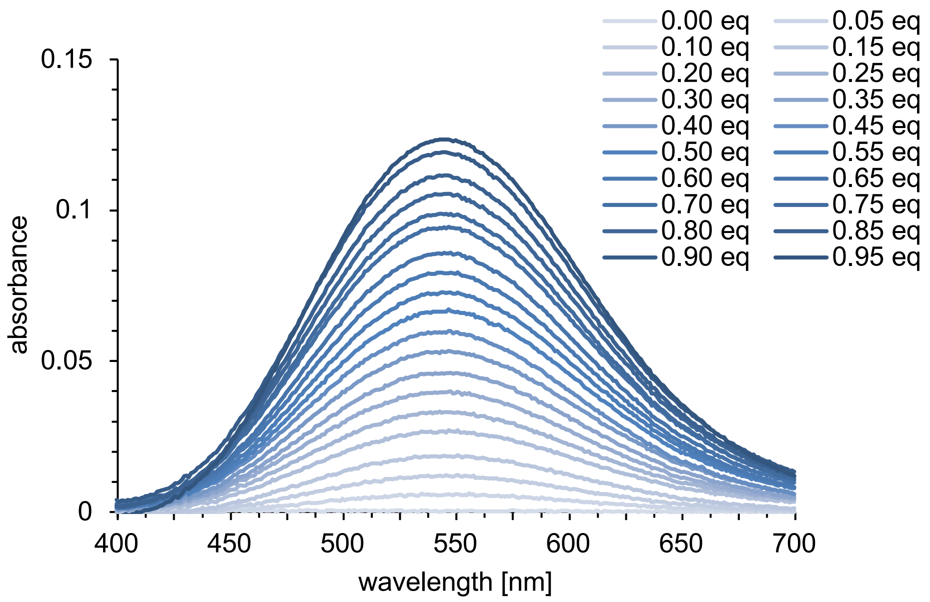

Figure S18. UV/VIS spectra in the range 400–700 nm of 2 mM peptide **c** with increasing concentrations of $CuCl_2$ (0.1 mM, 0.05 eq.) visualizing the d-d transition at λ_{max} = 545 nm of the forming Cu(II) ATCUN complex **3**. The saturation point is 0.95 eq. and was used to adjust the inaccurate peptide yield determined by weighing.

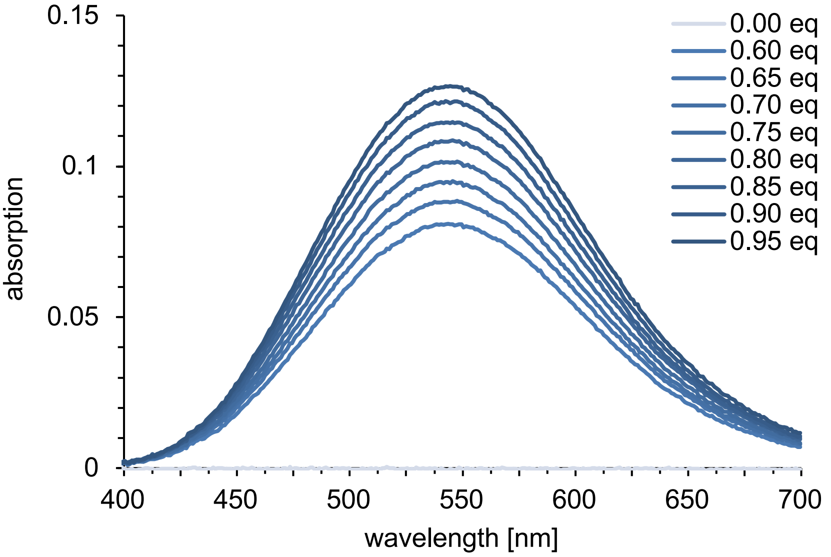

Figure S19. UV/VIS spectra in the range 400-700 nm of 2 mM peptide **d** with increasing concentrations of $CuCl_2$ (0.1 mM, 0.05 equiv.) visualizing the d-d transition at λ_{max} = 546 nm of the forming Cu(II) ATCUN complex **4**. The saturation point is 0.95 equiv. and was used to adjust the inaccurate peptide yield determined by weighing.

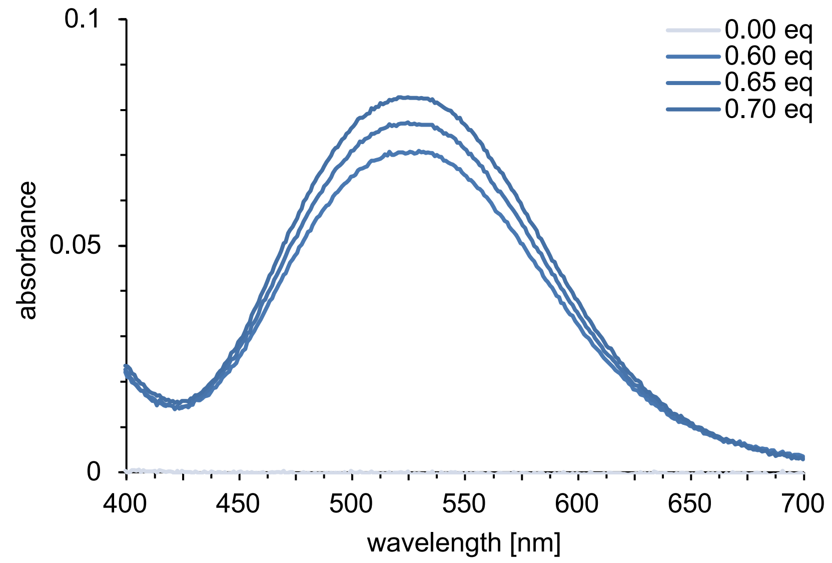

Figure S20. UV/VIS spectra in the range 400–700 nm of 2 mM peptide **e** with increasing concentrations of $CuCl_2$ (0.1 mM, 0.05 eq.) visualizing the d-d transition at λ_{max} = 529 nm of the forming Cu(II) ATCUN complex **5**. The saturation point is 0.70 eq. and was used to adjust the inaccurate peptide yield determined by weighing.

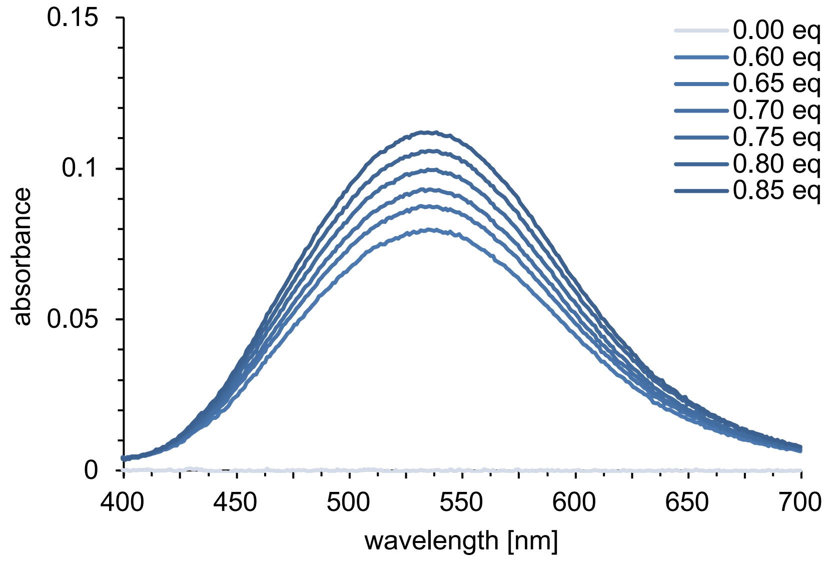

Figure S21. UV/VIS spectra in the range 400–700 nm of 2 mM peptide **f** with increasing concentrations of $CuCl_2$ (0.1 mM, 0.05 eq.) visualizing the d-d transition at λ_{max} = 537 nm of the occurring Cu(II) ATCUN complex **6.** The saturation point is 0.85 eq. and was used to adjust the inaccurate peptide yield determined by weighing.

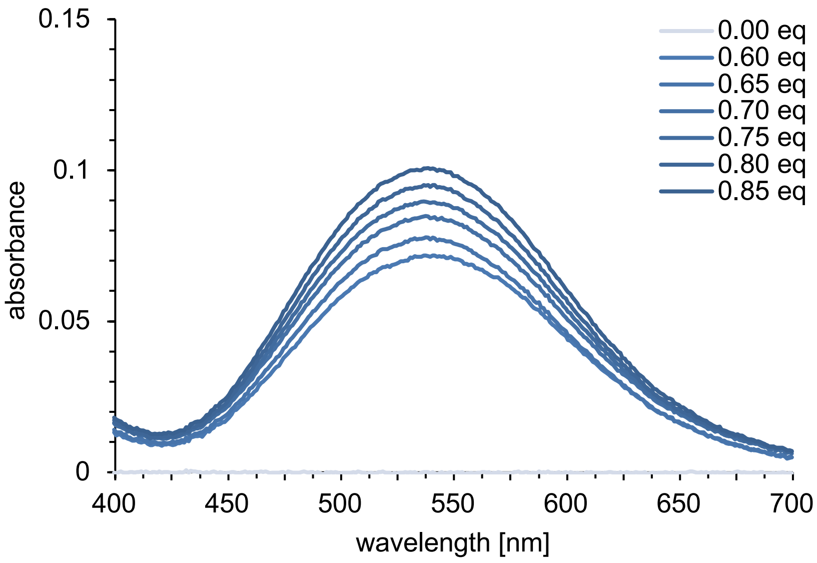

Figure S22. UV/VIS spectra in the range 400–700 nm of 2 mM peptide **g** with increasing concentrations of $CuCl_2$ (0.1 mM, 0.05 eq.), visualizing the d-d transition at λ_{max} = 541 nm of the forming Cu(II) ATCUN complex **7**. The saturation point is 0.85 eq. and was used to adjust the inaccurate peptide yield determined by weighing.

S-4 *In situ* Cu(II) complex synthesis

If not stated otherwise, in DNA cleavage and ROS detection experiments (S-6 and S-8) Cu(II) ATCUN complex solutions (**1–7**) were prepared *in situ* as follows: 500 µL of a 2.5 mM peptide solution (**a–g**) and 500 µL of a 2 mM $CuCl_2$ solution were combined resulting in 1 mL of a 1 mM Cu(II) complex solution (Cu(II):peptide = 1:1.25).
For the cell studies (cytotoxicity and cellular uptake) and all DNA interaction experiments (DNA melting curves, EtBr displacement assay and CD spectroscopy) the final Cu(II)-peptide ratio was 1:1.05 (a slightly lower ratio was used here in order to not additionally disturb the cellular metal homeostasis by applying an excess of chelator).
For DNA cleavage and binding studies as well as ROS detection, the *in situ* prepared Cu(II) ATCUN complex stock solutions were prepared in low concentrated MOPS buffer (5 mM, pH 7.4) to ensure complex formation before the actual experiment. Through dilution of these stock solutions for use in the above-mentioned experiments (50 mM MOPS buffer), the actual buffer concentration negligibly increased (50.25 mM).

<u>ESI-MS</u>

The *in situ* prepared Cu(II) ATCUN complexes **1–7** were characterized by ESI-MS. The calculated and found masses of the complexes are listed in Table S4. The ESI mass spectra are shown in Figures S23–S36.

Table S4. Calculated and found *m/z* values in ESI-MS of *in situ* prepared Cu(II) ATCUN complex solutions **1-7**. "L" stands for the corresponding peptide ligand **a–g**.

	**1** (L=**a**)	**2** (L=**b**)	**3** (L=**c**)	**4** (L=**d**)	**5** (L=**e**)	**6** (L=**f**)	**7** (L=**g**)
**calculated:**							
$[L+Cu-H]^+$	561.1351	703.2821	575.1508	717.2978	833.2664	775.2821	775.2821
$[L+Cu+Na-2H]^+$	583.1171	725.2641	597.1327	739.2797	855.2484	797.2641	797.2641
$[L+Cu]^+$			576.1587				
**found:**							
$[L+Cu-H]^+$	561.1352	703.2848	575.1512	717.2987	833.2661	775.2824	775.2812
$[L+Cu+Na-2H]^+$	583.1171	725.2666	597.1334	739.2808	855.2495	797.2648	797.2629
$[L+Cu]^+$			576.1580				

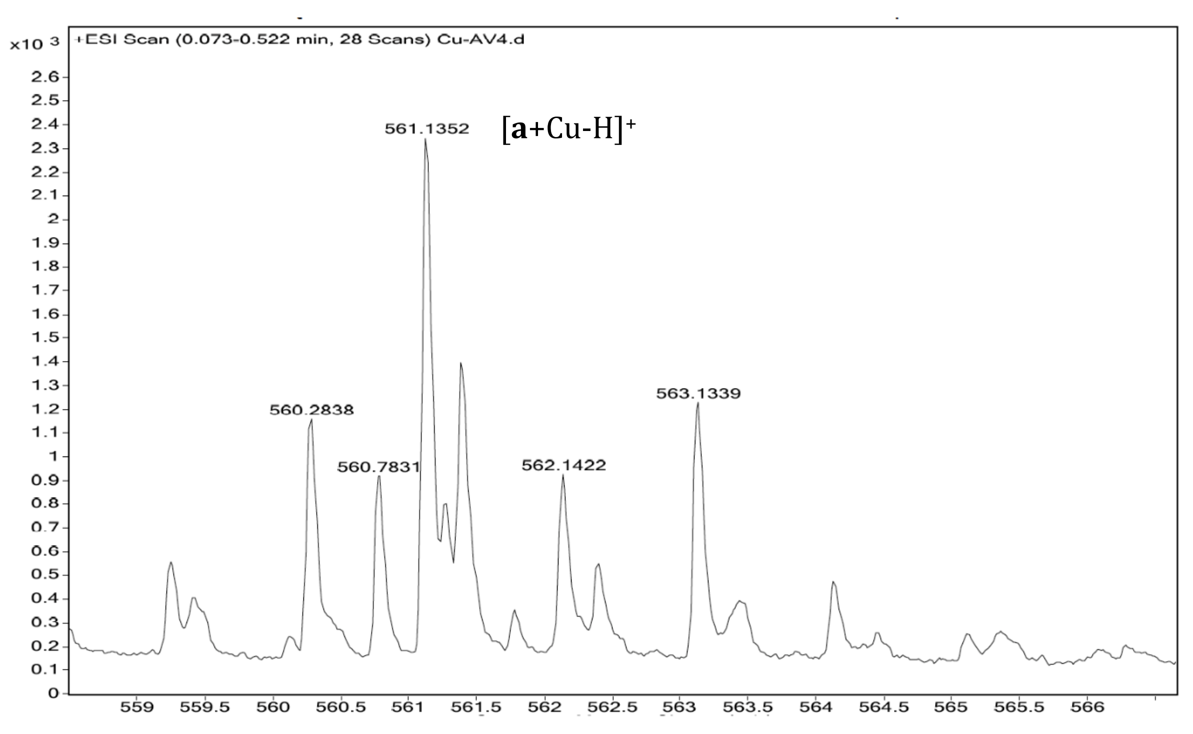

Figure S23. Zoom in (*m/z* 559–566) into the ESI mass spectrum of **1** after *in situ* preparation by combining solutions of **a** and $CuCl_2$. Dilution with MeCN.

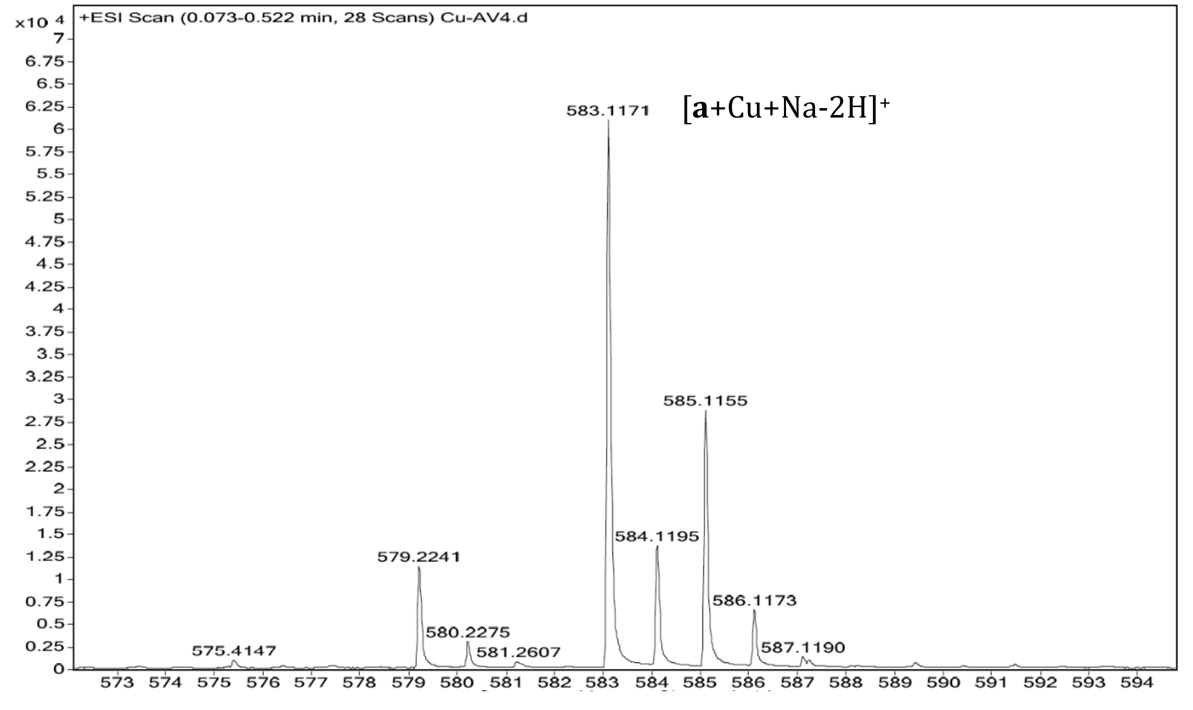

Figure S24. Zoom in (*m/z* 573–594) into the ESI mass spectrum of **1** after *in situ* preparation by combining solutions of **a** and $CuCl_2$. Dilution with MeCN.

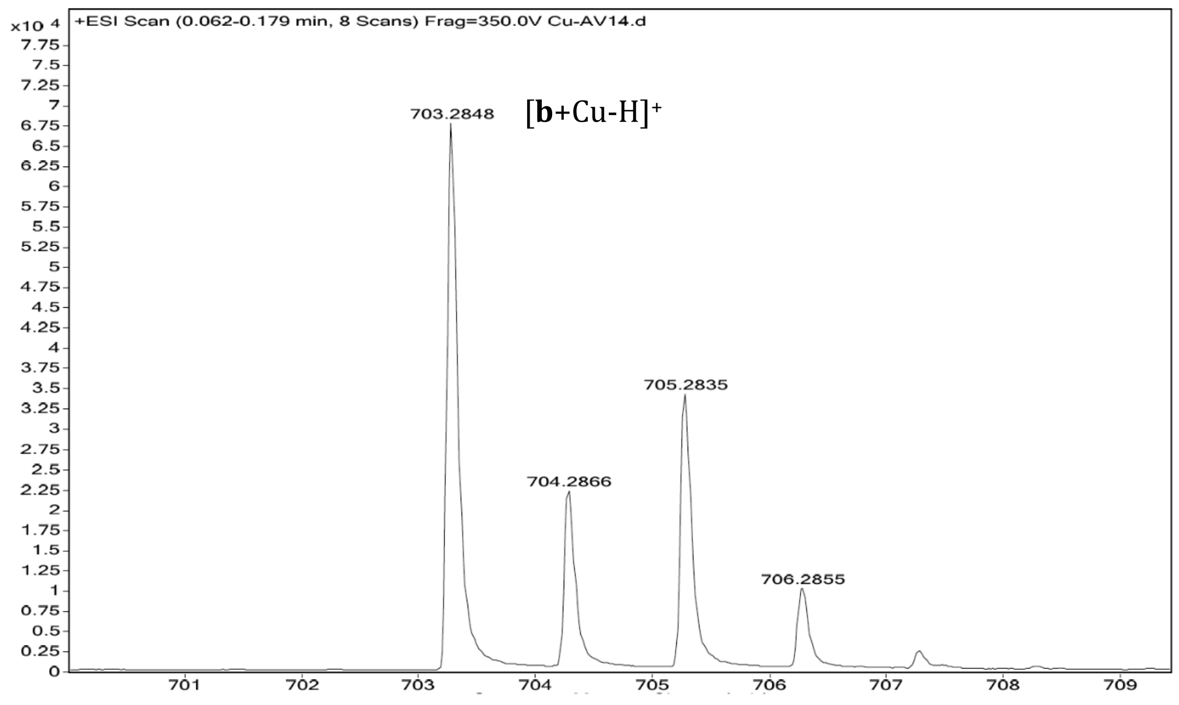

Figure S25. Zoom in (*m/z* 701–709) into the ESI mass spectrum of **2** after *in situ* preparation by combining solutions of **b** and $CuCl_2$. Dilution with MeCN.

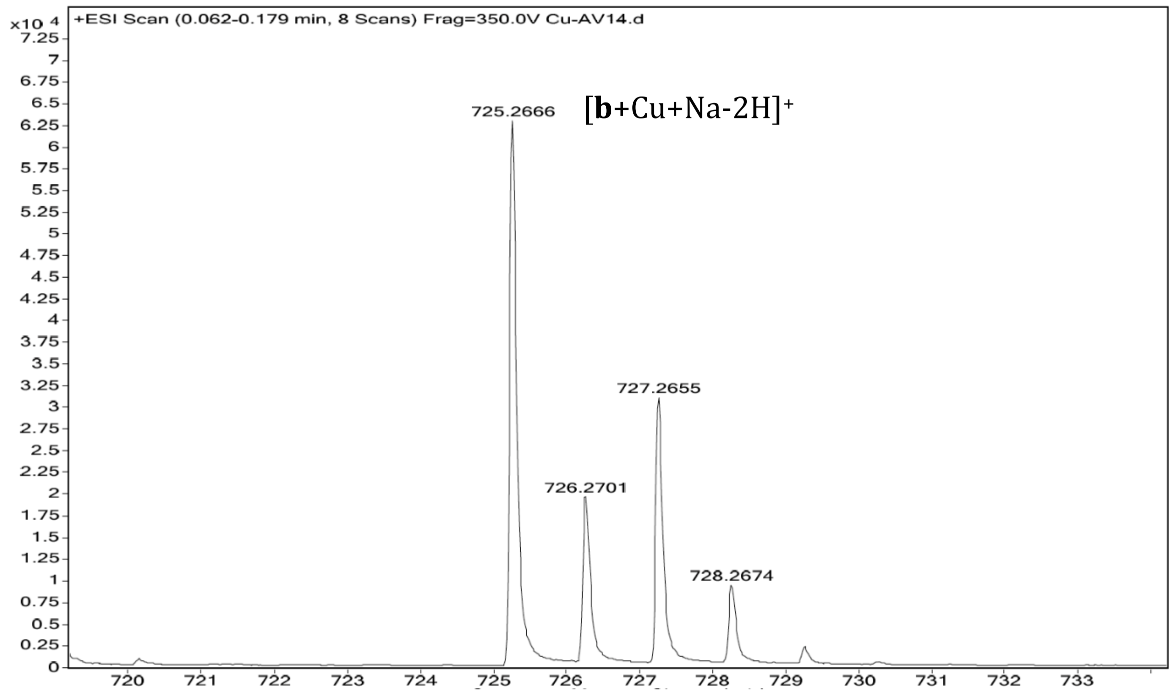

Figure S26. Zoom in (*m/z* 720–733) into the ESI mass spectrum of **2** after *in situ* preparation by combining solutions of **b** and $CuCl_2$. Dilution with MeCN.

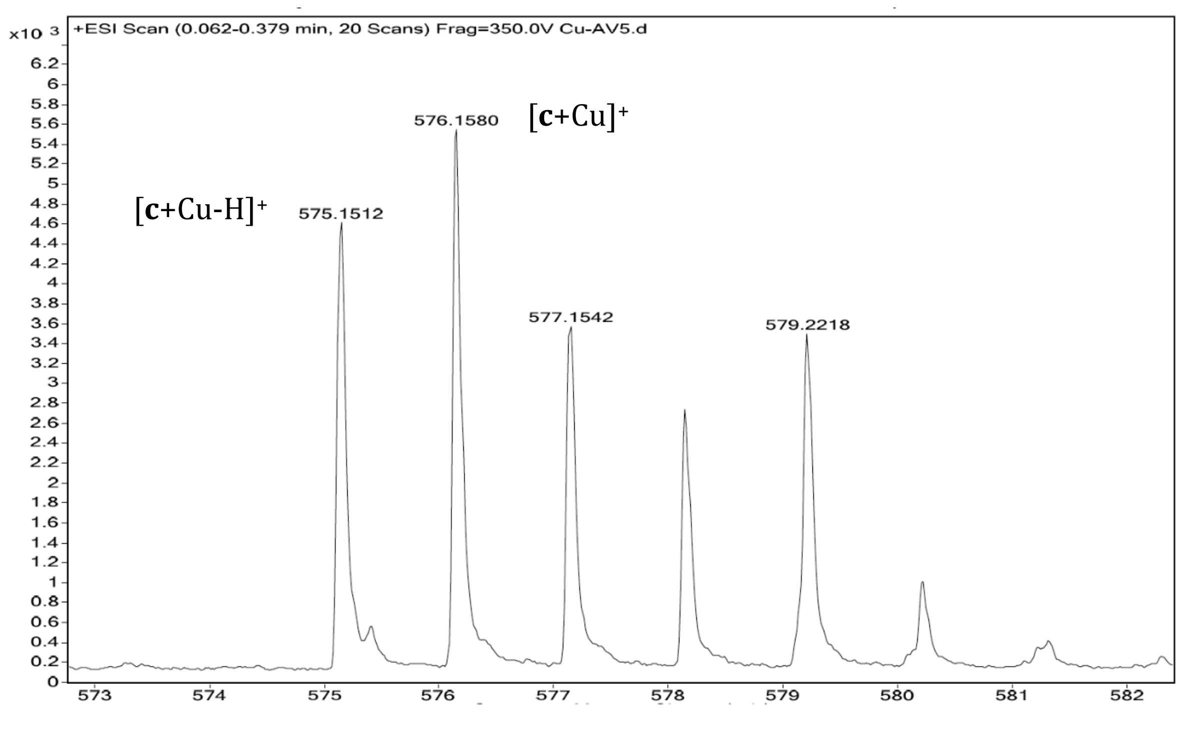

Figure S27. Zoom in (*m/z* 573–582) into ESI mass spectrum of **3** after *in situ* preparation by combining solutions of **c** and $CuCl_2$. Dilution with MeCN.

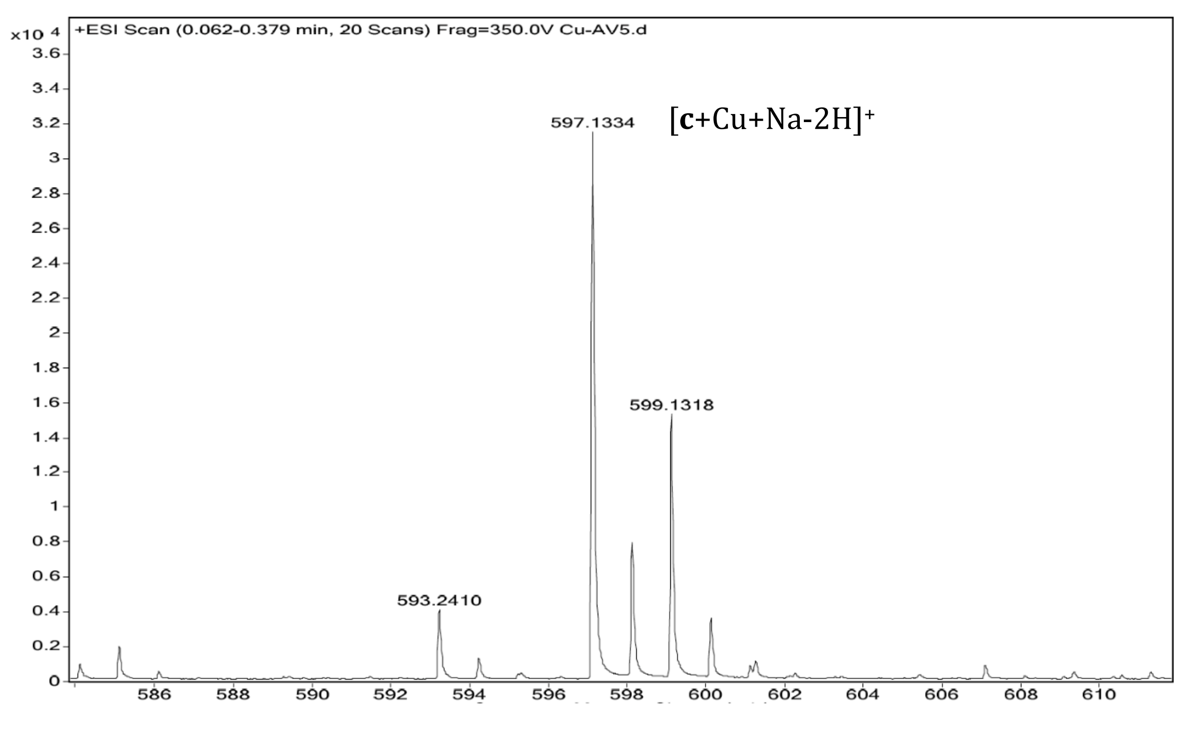

Figure S28. Zoom in (*m/z* 586–610) into the ESI mass spectrum of **3** after *in situ* preparation by combining solutions of **c** and $CuCl_2$. Dilution with MeCN.

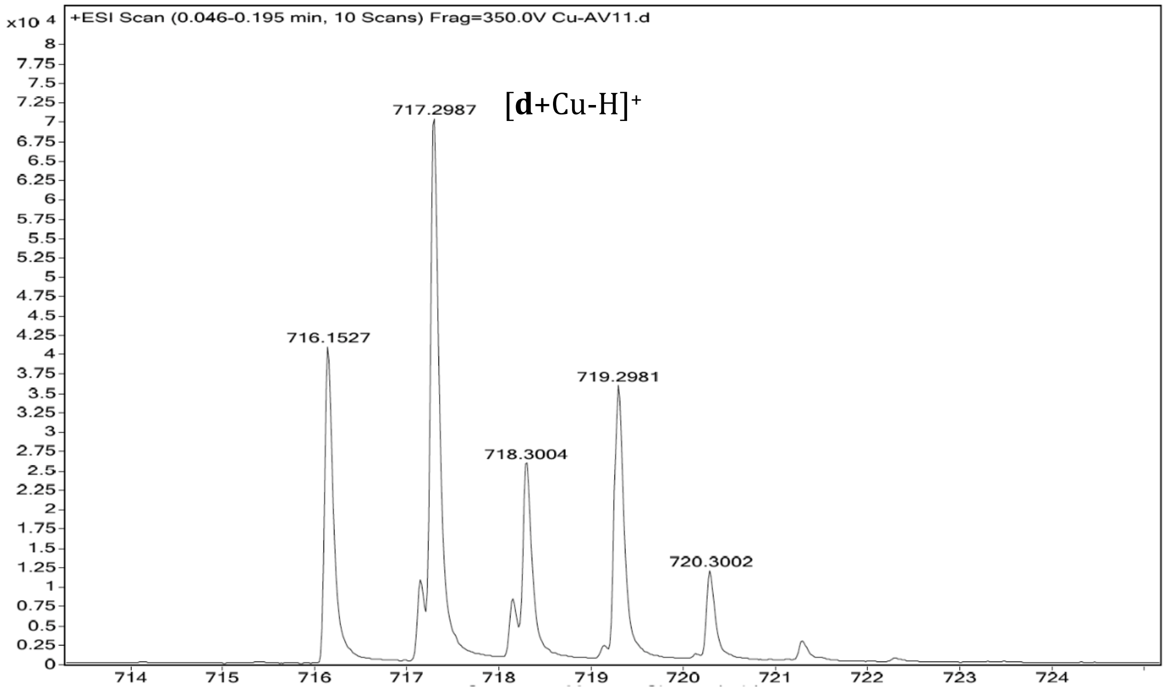

Figure S29. Zoom in (*m/z* 714–724) into the ESI mass spectrum of **4** after *in situ* preparation by combining solutions of **d** and $CuCl_2$. Dilution with MeCN.

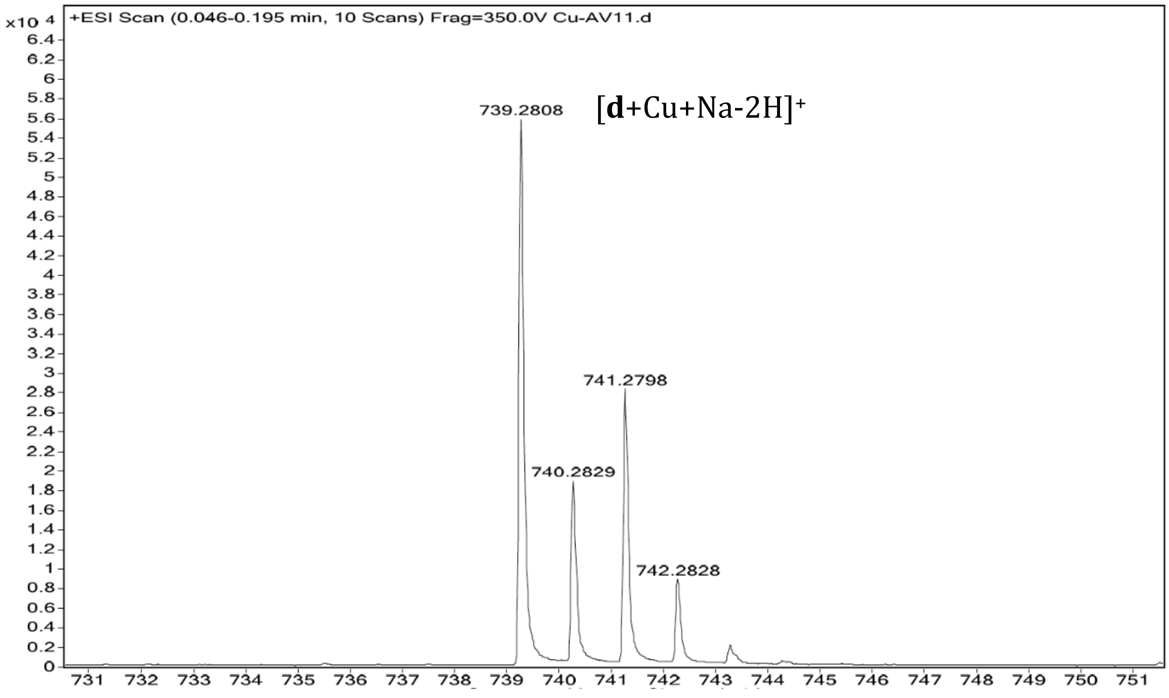

Figure S30. Zoom in (*m/z* 731–751) into the ESI mass spectrum of **4** after *in situ* preparation by combining solutions of **d** and $CuCl_2$. Dilution with MeCN.

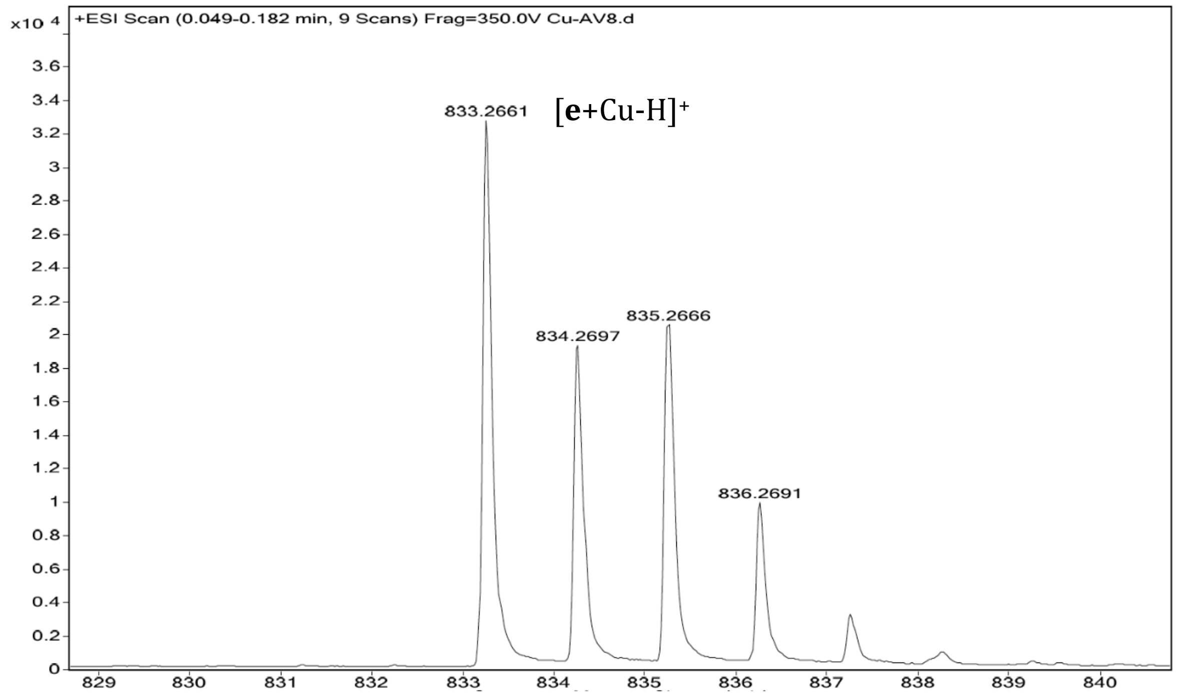

Figure S31. Zoom in (*m/z* 829–840) into the ESI mass spectrum of **5** after *in situ* preparation by combining solutions of **e** and $CuCl_2$. Dilution with MeCN.

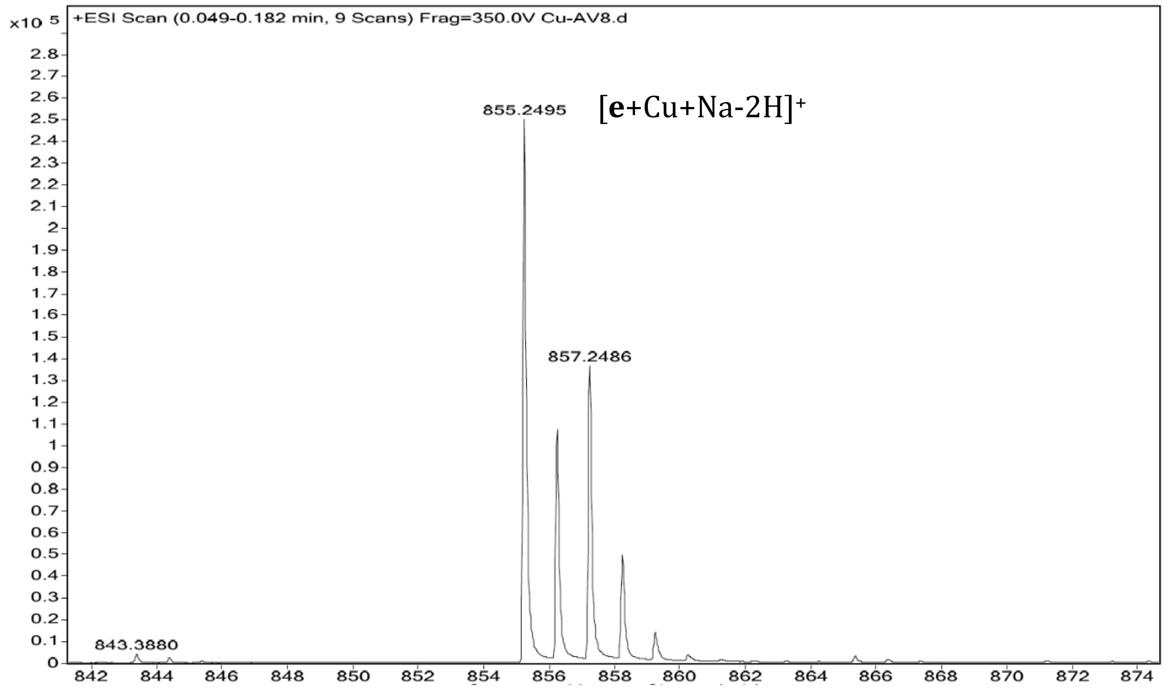

Figure S32. Zoom in (*m/z* 842–874) into the ESI mass spectrum of **5** after *in situ* preparation by combining solutions of **e** and $CuCl_2$. Dilution with MeCN.

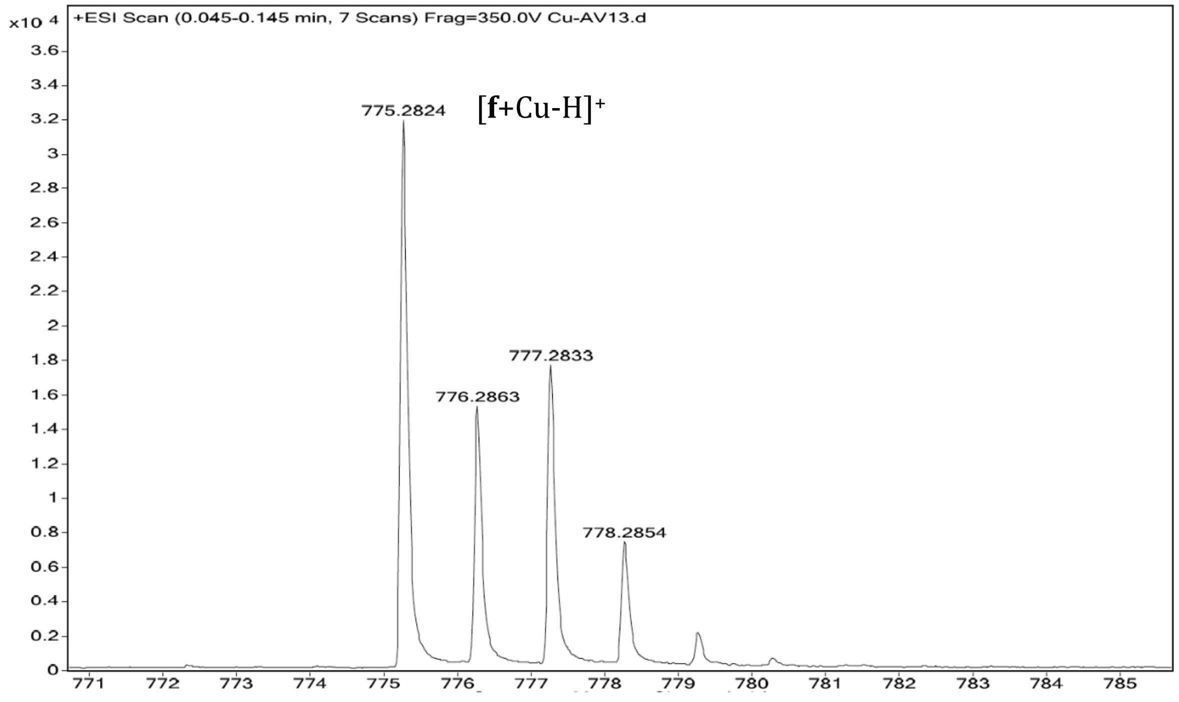

Figure S33. Zoom in (*m/z* 771–785) into the ESI mass spectrum of **6** after *in situ* preparation by combining solutions of **f** and $CuCl_2$. Dilution with MeCN.

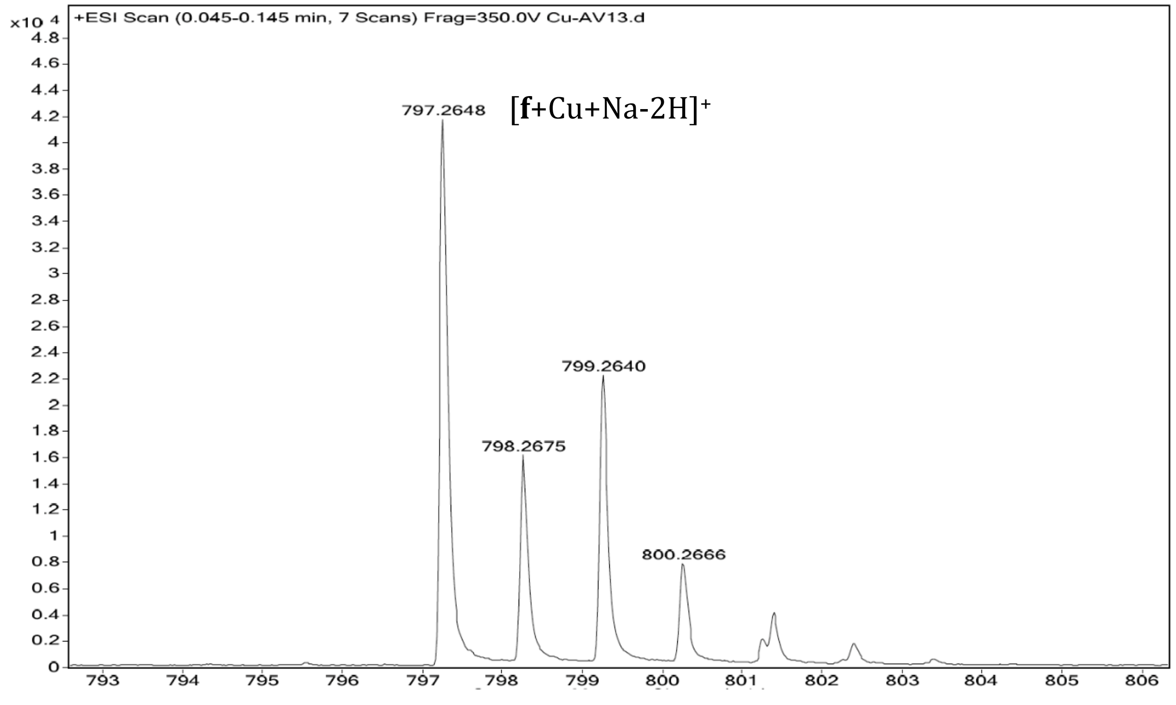

Figure S34. Zoom in (*m/z* 793–806) into the ESI mass spectrum of **6** after *in situ* preparation by combining solutions of **f** and $CuCl_2$. Dilution with MeCN.

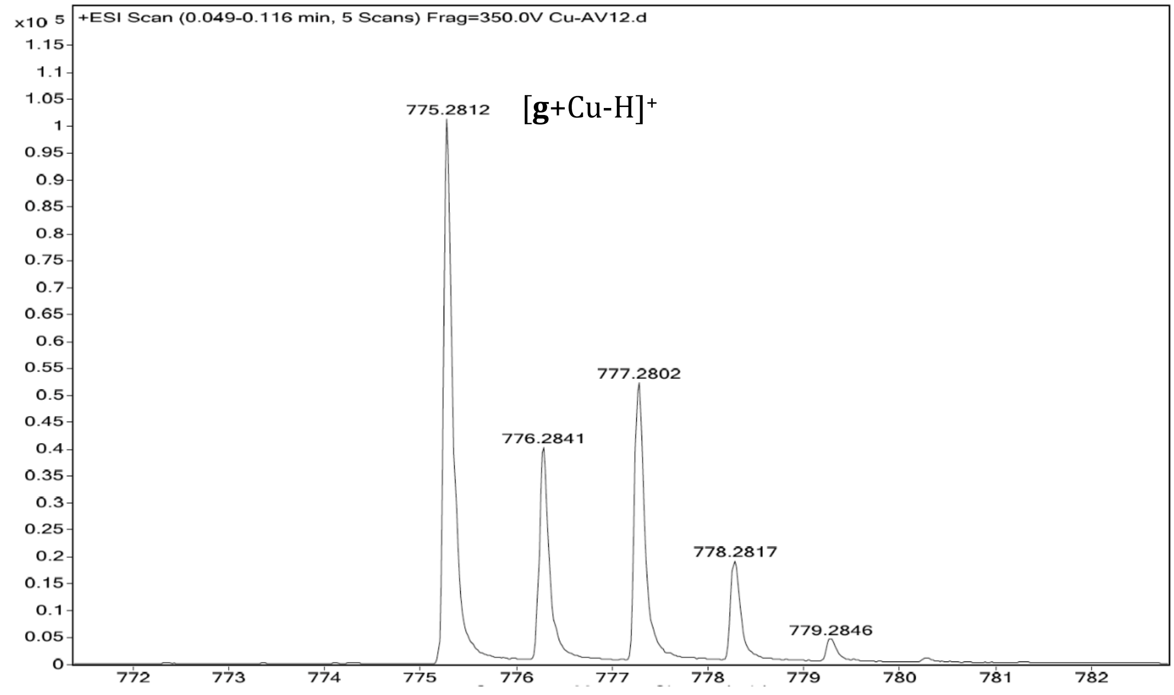

Figure S35. Zoom in (*m/z* 772–782) into the ESI mass spectrum of **7** after *in situ* preparation by combining solutions of **g** and $CuCl_2$. Dilution with MeCN.

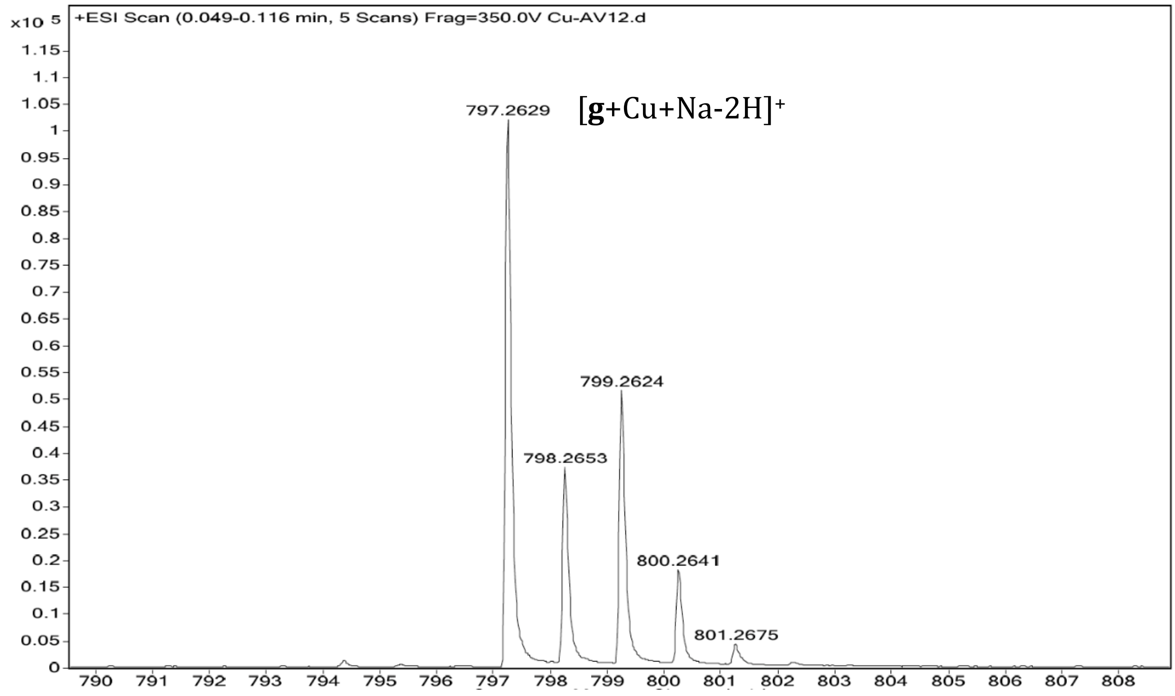

Figure S36. Zoom in (*m/z* 790–808) into the ESI mass spectrum of **7** after *in situ* preparation by combining solutions of **g** and $CuCl_2$. Dilution with MeCN.

S-5 Protonation and complex stability constants

All bioanalytical experiments (DNA cleavage, DNA binding and ROS detection studies) and cell studies (cytotoxicity and cellular uptake) were carried out at pH 7.4. To characterize the speciation of the ATCUN metallopeptides at this pH, potentiometric titrations in combination with UV/VIS and CD spectroscopy were carried out for **1** and **3**-**7**.

S-5.1 Potentiometry, UV/VIS and circular dichroism (CD) spectroscopy

Potentiometry

Potentiometric titrations were performed on a *907 Titrando Automatic Titrator* (*Metrohm*) using a *Biotrode* combined glass electrode (*Metrohm*), calibrated daily by nitric acid titrations with the use of standardized 100 mM NaOH (carbon dioxide-free) as a titrant.

Stock solution preparation:
Stock solutions of peptides **a** and **c–g** for UV/VIS and CD experiments were prepared by dissolving given amounts of peptides in carbon dioxide-free Milli-Q® water.

Ligand titration:
Samples (1.5 mL volume, 1 mM peptide) were prepared in 4 mM HNO_3/96 mM KNO_3 solution. To calculate ligand (peptide) concentrations and their protonation constants, at least three consecutive potentiometric titrations were performed.

Complex titration:
Three samples (1.5 mL) of 1 mM peptide and $CuCl_2$ with Cu : peptide ratios of 1:1, 1:2 and 1:3 were prepared in 4 mM HNO_3/96 mM KNO_3. Each sample was titrated separately using a standardized 100 mM NaOH solution. The pH range for all potentiometric titrations was 2.7-11.6.

All ligand and complex titrations were performed under argon at 25 °C. The data was analyzed using the SUPERQUAD and HYPERQUAD programs.

UV/VIS spectroscopy

For the determination of Cu(II) stability constants, peptides **a** and **c–g** UV/VIS spectra were recorded in a range of 250–900 nm with a scan resolution of 1 nm, and a scan speed of 0.5 s/nm on a *Lambda 950 UV/VIS/NIR spectrophotometer* (*PerkinElmer*). The samples of 1 mM peptide/0.8 mM $CuCl_2$ were prepared in 1 cm path length quartz cuvettes (*Hellma*) and titrated with small amounts of concentrated NaOH solution. Usually the pH-metric titration spanned from pH 2.9 to pH 12.5.

For the determination of Cu(I) stability constants, a 100 mM stock solution of ferrozine (Fz) was prepared in Milli-Q® water. A 200 mM $[Cu(MeCN)_4]BF_4$ stock solution in MeCN was diluted to 10 mM in HEPES (100 mM, pH 7.4) in Milli-Q® water (v/v 10% MeCN). A 2 mM complex solution of $[CuFz_2]^{3-}$ (Cu(I):Fz ratio = 1:2.1) was prepared *in situ* in HEPES

(100 mM, pH 7.4) in Milli-Q® water (v/v 2% MeCN). The solutions of peptides **a** and **c** (10 mM) were prepared with an additional amount of ascorbic acid (1 eq., 10 mM) in Milli-Q® water. The $[CuFz_2]^{3-}$ complex and the peptide solutions were degassed with Ar immediately before use in the titration experiment.
For the UV/VIS titration experiment, 1000 µL of $[CuFz_2]^{3-}$ (50 µM, Cu(I):Fz ratio = 1:2.1) in HEPES (100 mM, pH 7.4) in Milli-Q® water (v/v 0.05% MeCN) in 1 cm path length quartz cuvettes (1.4 mL, *Hellma*) and titrated successively with peptide solutions in 100 µM steps (100–500 µM) at 25 °C. After each addition the solutions were mixed and allowed to equilibrate for 2 min before collecting the UV/VIS spectra.
K_{app} values (Cu(I) complex stability constants) were calculated by considering the decrease of the characteristic absorption band of $[CuFz_2]^{3-}$ at 470 nm (ε = 4320 M^{-1} cm^{-1}) during the peptide titration (Figure S47 and S48), the association constant of Fz towards Cu(I) (β = 3.7 x 10^{11} M^{-2}) and the used concentrations of $[CuFz_2]^{3-}$ (50 µM Cu(I), 105 µM Fz) and peptides (100–500 µM). For each peptide addition (100 µM) a K_{app} value was calculated. All obtained K_{app} values were averaged with the corresponding standard deviation (Table S7).[2]

CD spectroscopy

CD measurements were carried out on a *J-815 CD spectrometer* (*JASCO*) over the spectral range of 250-800 nm, using a scanning speed of 2 s/nm and a scan resolution of 1 nm. Samples were prepared analogously to UV/VIS spectroscopy samples.

Table S5. Logarithmic protonation and Cu(II) binding constants (log *β* and p*K*) of peptides **a-g** (**b** not measured), determined by potentiometry at 25 °C and *I*=0.1 (KNO_3), and competitivity index (CI) values calculated at pH 7.4 for 1 mM Cu(II) and peptides based on respective stability constants. Statistical errors on the last digits of log *β* values, provided by HYPERQUAD, are given in parentheses.

Species	a		c		d		e		f		g	
	log *β*	p*K*	log *β*	p*K*	log *β*	p*K*	log *β*	p*K*	log *β*	p*K*	log *β*	p*K*
H_4L	-	-	-	-	33.875(4)	6.02[a]	-	-	-	-	-	-
H_3L	-	-	-	-	27.860(3)	7.30[b]	-	-	23.715(5)	6.08[a]	23.846(5)	6.09[a]
H_2L	14.160(2)	6.30[a]	14.266(3)	6.26[a]	20.563(2)	9.91[c]	13.700(3)	6.12[a]	17.632(4)	7.37[b]	17.755(5)	7.50[b]
HL	7.853(1)	7.85[b]	8.001(2)	8.00[b]	10.656(3)	10.66[c]	7.584(3)	7.58[b]	10.262(3)	10.26[c]	10.255(3)	10.25[c]
CuH_3L	-	-			-	-	-	-	-	-	-	-
CuH_2L	-	-			26.604(9)	-	-	-	-	-	-	-
CuHL	-	-			-	-	-	-	16.406(9)	-	16.75(1)	-
CuL	-	-	7.137(5)		16.338(4)	5.13	6.743(7)	-	-	-	-	-
$CuH_{-1}L$	-	-			6.337(8)	10.00	-	-	6.280(3)	5.06	7.108(5)	4.82
$CuH_{-2}L$	-0.587(5)	-	-4.160(5)	5.65	-4.068(6)	10.41	-3.104(3)	4.92	-3.840(7)	10.12	-2.987(9)	10.10
$CuH_{-3}L$	-12.38(2)	11.79	-14.560(8)	10.4	-	-	-14.683(9)	11.58	-15.007(8)	11.17	-	-
log $^*K_{4N}$	-14.747		-18.426		-17.537		-16.804		-17.435		-16.738	
CI [M^{-1}]	13.61		9.915		10.31		11.28		10.52		11.29	

[a] His imidazole
[b] N-terminal amine
[c] Lys amine

CI: Competitivity index calculated for pH 7.4 and peptide L, Cu(II) and Z concentrations of 0.001 M. CI is log K_{CuZ} fulfilling the condition $\Sigma_{ijk}([Cu_iH_jL_k]=[CuZ]$.[3]
log $^*K_{4N}$ is a protonation-corrected stability constant allowing to compare peptides with different protonation states forming the same type of complexes
In general log$^*K_{xN}$, where *x* stand for number of coordinated nitrogens can be described by **log $^*K_{xN}$ = log $\beta\{CuH_{n-x}L\}$ – log $\beta\{H_nL\}$**
H_nL stands for the protonation of coordinating histidine (in position 3 from N-terminus in case of ATCUN motifs)

a: log$^*K_{4N}$ = log $\beta\{CuH_{-2}L\}$ – log $\beta\{H_2L\}$ = -0.587 – 14.160 = -14,747
c: log$^*K_{4N}$ = log $\beta\{CuH_{-2}L\}$ – log $\beta\{H_2L\}$ = -4.160 – 14.266 = -18.426
d: log$^*K_{4N}$ = log $\beta\{CuL\}$ – log $\beta\{H_4L\}$ = 16.338 – 33.875 = -17.537
e: log$^*K_{4N}$ = log $\beta\{CuH_{-2}L\}$ – log $\beta\{H_2L\}$ = -3.104 – 13.700 = -16.804
f: log$^*K_{4N}$ = log $\beta\{CuH_{-1}L\}$ – log $\beta\{H_3L\}$ = 6.280 – 23.715 = -17.435
g: log$^*K_{4N}$ = log $\beta\{CuH_{-1}L\}$ – log $\beta\{H_3L\}$ = 7.108 – 23.846 = -16.738

Peptides can be compared easily between each other using both protonation-corrected stability constant (log $^*K_{4N}$) and Competitivity Index at pH 7.4 ($CI_{7.4}$):
For log $^*K_{4N}$, the higher the number (less negative value) the higher stability of Cu(II) complexes (only relative meaning – no interpretation of concentrations of species possible).
For $CI_{7.4}$, the higher the number the higher the stability. CI is the affinity constant for the imaginary CuZ complex which is equivalent in strength to the probed complex/set of complexes. The advantage of CI is that its unit is M^{-1}, and can be compared with log *K* values for real complexes.

In Figure 2 and Figures S37-S40 the pH-dependent species distribution diagrams of Cu(II) complexes of **a** and **c–g** are shown. The pH-metric UV/VIS and CD titrations of Cu(II) complexes of **a** and **c–g** are given in Figures S41–S46.

p*K* values of the peptides (Table S5 and S6) correspond to the deprotonation steps of the N-terminal amine (values 7.3–8.0) and imidazole nitrogen atoms (values 6.0–6.3). Additionally, the peptides **d**, **f**, and **g** exhibit protonation constants around 9.9–10.7, which are assigned to amine deprotonation of Lys side chain. Furthermore, observed deprotonations at around 12, assumed to correspond to Ser hydroxyl or the second imidazole nitrogen, were omitted in subsequent calculations due to the limit of the method's reliability (alkaline error range of glass electrodes).

Table S6. Assignment of deprotonation events in Cu(II) species of peptides **a** and **c–g** as determined *via* potentiometry, UV/VIS and CD spectroscopy.

	Peptide sequence	Cu(II) species	Deprotonation assignment
**a**	Gly-Gly-His-Gly-Ser-Ser-$CONH_2$	$CuH_{-2}L$	**4N:** NH_2^{term}, N_{im}, 2x N^-
		$CuH_{-3}L$	**4N:** NH_2^{term}, N_{im}, 2x N^-, (Ser or second N_{im})
**c**	Gly-β-Ala-His-Gly-Ser-Ser-$CONH_2$	CuL	**2N:** NH_2^{term}, N_{im}
		$CuH_{-2}L$	**4N:** NH_2^{term}, N_{im}, 2x N^-
		$CuH_{-3}L$	**4N:** NH_2^{term}, N_{im}, 2x N^-, (Ser or second N_{im})
**d**	Lys-β-Ala-His-Lys-Ser-Ser-$CONH_2$	CuH_2L	**2N:** NH_2^{term}, N_{im}
		CuL	**4N:** NH_2^{term}, N_{im}, 2x N^-
		$CuH_{-1}L$	**4N:** NH_2^{term}, N_{im}, 2x N^-, N^{ε}Lys
		$CuH_{-2}L$	**4N:** NH_2^{term}, N_{im}, 2x N^-, 2x N^{ε}Lys
**e**	Trp-β-Ala-His-Trp-Ser-Ser-$CONH_2$	CuL	**2N:** NH_2^{term}, N_{im}
		$CuH_{-2}L$	**4N:** NH_2^{term}, N_{im}, 2x N^-
		$CuH_{-3}L$	**4N:** NH_2^{term}, N_{im}, 2x N^-, (Ser or second N_{im})
**f**	Lys-β-Ala -His-Trp-Ser-Ser-$CONH_2$	CuHL	**2N:** NH_2^{term}, N_{im}
		$CuH_{-1}L$	**4N:** NH_2^{term}, N_{im}, 2x N^-
		$CuH_{-2}L$	**4N:** NH_2^{term}, N_{im}, 2x N^-, N^{ε}Lys
		$CuH_{-3}L$	**4N:** NH_2^{term}, N_{im}, 2x N^-, N^{ε}Lys, (Trp, Ser or second N_{im})
**g**	Trp-β-Ala -His-Lys-Ser-Ser-$CONH_2$	CuHL	**2N:** NH_2^{term}, N_{im}
		$CuH_{-1}L$	**4N:** NH_2^{term}, N_{im}, 2x N^-
		$CuH_{-2}L$	**4N:** NH_2^{term}, N_{im}, 2x N^-, N^{ε}Lys

The potentiometric data for peptide **a** containing an ATCUN motif (with Gly in position 2, Gly2) suggests that three copper species are formed in a pH range of 2.5-12, that is: (i) a Cu(II) hexaaqua ion, being the only detectable species up to pH 3.7, (ii) the 4N complex ($CuH_{-2}L$) with a p*K* value of 4.50 (based on UV/VIS spectroscopic data), and (iii) a $CuH_{-3}L$ species appearing at highly alkaline pH (p*K* 11.79) suggesting deprotonation (but without direct Cu(II) coordination) of the Ser hydroxyl or the second imidazole nitrogen atom (Figure 2, A). pH-metric UV/VIS spectra of **a** in presence of Cu(II) (Figure S41, left) agree with the potentiometric data. At low pH a band at 816 nm corresponds to Cu(II) hexaaqua ion. Upon increase of pH, a distinctive 4N complex band at 529 nm appears. A single isosbestic point around 685 nm proves the presence of only one species up to pH 9.
pH-metric CD spectra of **a** in presence of Cu(II) (Figure S44, left) are characteristic of an ATCUN coordination type with a visible positive charge transfer (CT) band at 306 nm and a double d-d band (maximum: 503 nm, minimum: 596 nm). Above pH 9, no significant

shift/alteration of band energies (ellipticity drop and broadening in CT region; increase of ellipticity in positive and negative d-d band) suggests second-sphere interactions, assumable by deprotonation of the second imidazole nitrogen or Ser hydroxyl.

Peptide **c** (Gly1/4) with β-Ala in position 2 (β-Ala2) allows Cu(II) species formation as in the case of **a**, though the Cu(II) hexaaqua ion is coordinated simultaneously in a 2N fashion from pH 4 up to pH 5.6, which is caused by a higher flexibility of the peptide backbone due to the additional CH_2 group. The absence of d-d bands in CD spectra (Figure S44, right) is in accordance with a 2N binding mode, involving the terminal amine and imidazole nitrogen atoms. With increasing pH, a square planar 4N complex is formed (denoted as a $CuH_{-2}L$ in Figure S37), and above pH 6.8 it is the solely present species (Cu(II) ATCUN complex). At very alkaline pH the $CuH_{-3}L$ complex is observed, presumably with a second sphere coordination by either the deprotonated Ser hydroxyl or the second imidazole nitrogen atom (see UV/VIS and CD data in Figure S41, right and S44, right). In the UV/VIS spectra the peak assigned to the Cu(II) hexaaqua ion at 816 nm (pH < 4) gradually changes to a band with a maximum at 650 nm (2N macro chelation). At around pH 4.8 transition to a 4N ATCUN complex occurs with its λ_{max} at 544 nm. The pK values are in accordance with the 4N ATCUN complex detectable in CD and UV/VIS.[4]

For the other β-Ala peptides **d–g** the species distribution is similar up to pH 8 (Figure 2, B and Figures S38-S40) based on UV/VIS and CD spectra (see Figure S42-S43 and Figure S45–S46). At pH < 4 the Cu(II) hexaaqua ion is observed; with the pH increasing the 2N complex is present (protonation states of CuH_2L, CuL, CuHL and CuHL for **d**, **e**, **f** and **g**, respectively, due to the presence of protonated Lys residue(s)). At pH 4–6, through rearrangement, the 4N ATCUN complexes (terminal NH_2, 2x N^- and N_{im} coordination) are formed, which are the solely present Cu(II) complexes in the pH range of 6 to 8.
Above pH 8, **e** exhibits one more species: $CuH_{-3}L$ (second-sphere interaction by assumably deprotonated Ser hydroxyl or the second imidazole nitrogen atom). β-Ala2 peptide **d** (Lys1/4) exhibits two additional 4N species due to deprotonated Lys residues: $CuH_{-1}L$ with pK of 10.00 and $CuH_{-2}L$ with pK of 10.41. For β-Ala2 peptide **g** (Trp1/Lys4) only one additional species of $CuH_{-2}L$ (deprotonated Lys) was detected above pH 8. Surprisingly, at high pH values, in addition to the 4N complex with deprotonated Lys ($CuH_{-2}L$ as for **g**), **f** (Lys1/Trp4) is suggested to form an additional species $CuH_{-3}L$, which has its origin in either the deprotonated Trp, the Ser hydroxyl or the second imidazole nitrogen atom. Based on the dramatic change in the CD spectrum of **f** at pH > 9 in comparison to **c–e** and **g**, deprotonation of Trp is more favored, probably causing interaction with the first coordination sphere. Although the Trp residue in **g** (Trp in position 1) is in closer proximity to the first coordination sphere as compared to **f** (Trp in position 4), the suggested deprotonation behavior of Trp was not observed for **g** by CD spectroscopy at pH > 9. This is presumably due to electronic changes in the Trp residue by coordination of the Trp amide N^- to Cu(II).

The competitivity index (CI, Table 1) is a useful measure for comparing the metal binding abilities of various ligand sets. For CI calculation, a hypothetical complex CuZ is assumed (Z stands for a virtual competitor towards the real peptide ligand). The CI stability constant of the CuZ complex fulfills the conditions of an equal concentration (50/50) of

Cu(II) complexes with studied and virtual peptides at chosen conditions (pH 7.4 and 1 mM concentrations of all components).[3]

S-5.2 pH-dependent species distribution of Cu(II) ATCUN complexes

Based on potentiometric titrations for the peptides and Cu(II) complexes and pH-metric UV/VIS and CD titrations of the Cu(II) complexes, the pH-dependent species distribution of Cu(II) complexes of **a** and **c–g** were obtained (Figures 2 and S37–S40).

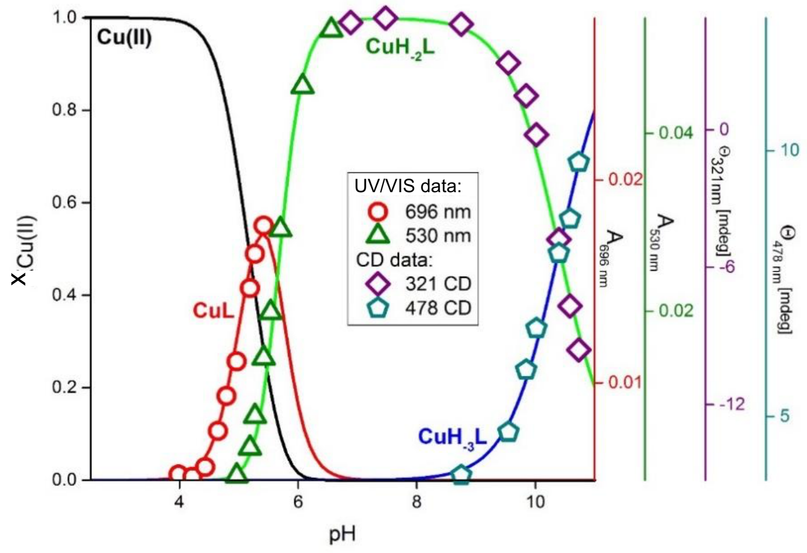

Figure S37. Species distribution for Cu(II)L (L=**c**) at 25 °C, calculated for concentrations used in UV/VIS and CD titrations (1.0 mM peptide and 0.8 mM $CuCl_2$) based on stability constants presented in Table S5. The y axis on the left indicates molar fractions x of Cu(II) complexes, which are color-coded as follows: $[Cu(H_2O)_6]^{2+}$, black; Cu(II) 2N complex, red; Cu(II) 4N complexes, green and blue. The y axes on the right provide values of absorbance (UV/VIS): red circles (696 nm) and green triangles (530 nm); and ellipticity (CD): purple diamonds (321 nm) and dark cyan pentagons (478 nm).

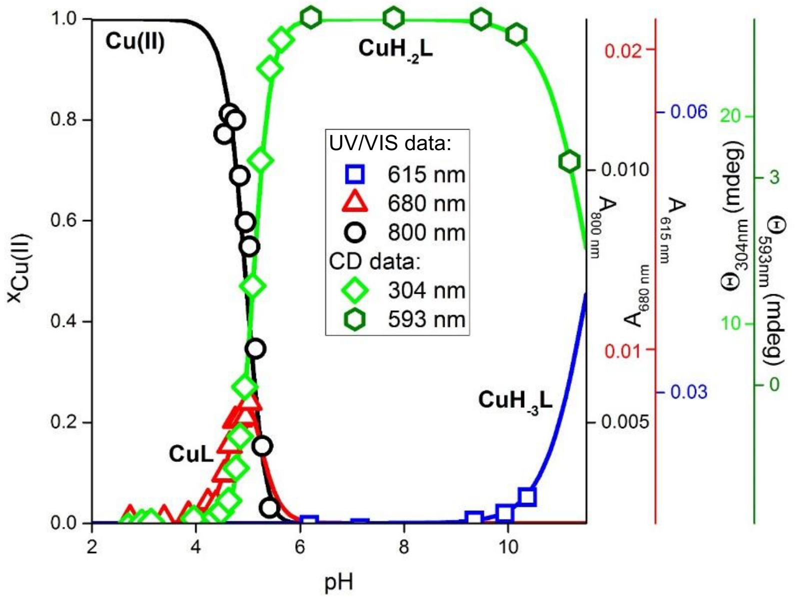

Figure S38. Species distribution for Cu(II)L (L=**e**) at 25 °C, calculated for concentrations used in UV/VIS and CD titrations (1.0 mM peptide and 0.8 mM $CuCl_2$) based on stability constants presented in Table S5. The y axis on the left indicates molar fractions x of Cu(II) complexes, which are color-coded as follows: $[Cu(H_2O)_6]^{2+}$, black; Cu(II) 2N complex, red; Cu(II) 4N complexes, green and blue. The y axes on the right provide values of absorbance (UV/VIS): black circles (800 nm), blue squares (615 nm) and red triangles (680 nm); and ellipticity (CD): green diamonds (304 nm) and olive hexagons (593 nm).

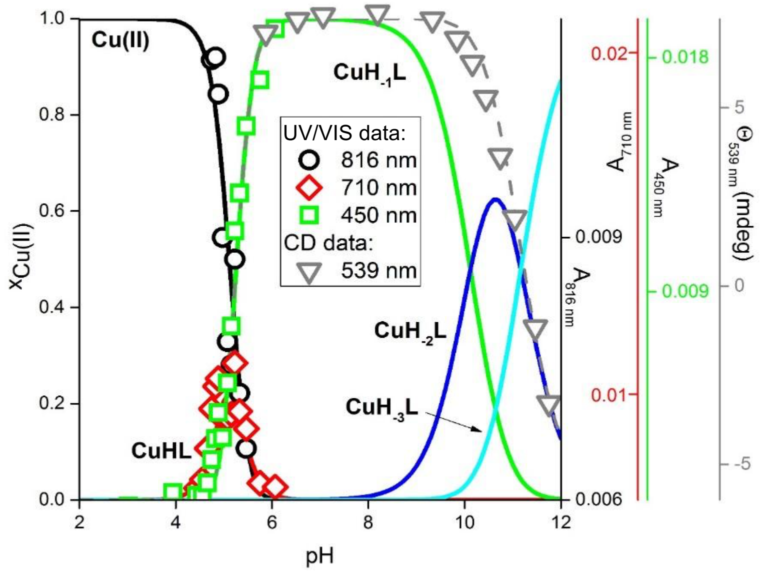

Figure S39. Species distribution for Cu(II)HL (HL=**f**) at 25 °C, calculated for concentrations used in UV/VIS and CD titrations (1.0 mM peptide and 0.8 mM $CuCl_2$) based on stability constants presented in Table S5. The y axis on the left indicates molar fractions x of Cu(II) complexes, which are color-coded as follows: $[Cu(H_2O)_6]^{2+}$, black; Cu(II) 2N complex, red; Cu(II) 4N complexes, green and blue. Dashed gray line represents an average of $CuH_{-1}L$ and $CuH_{-2}L$ complexes. The y axes on the right provide values of absorbance (UV/VIS): black circles (816 nm), red diamonds (710 nm) and green squares (450 nm); and ellipticity (CD): gray triangles (539 nm).

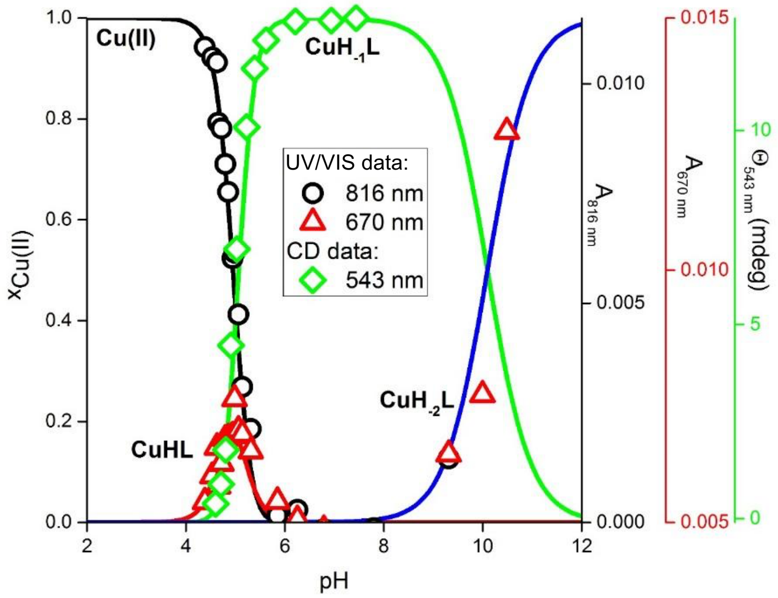

Figure S40. Species distribution for Cu(II)HL (HL=**g**) at 25 °C, calculated for concentrations used in UV/VIS and CD titrations (1.0 mM peptide and 0.8 mM $CuCl_2$) based on stability constants presented in Table S5. The y axis on the left indicates molar fractions x of Cu(II) complexes, which are color-coded as follows: $[Cu(H_2O)_6]^{2+}$, black; Cu(II) 2N complex, red; Cu(II) 4N complexes, green and blue. The y axes on the right provide values of absorbance (UV/VIS): black circles (816 nm) and red triangles (670 nm); and ellipticity (CD): green diamonds (543 nm).

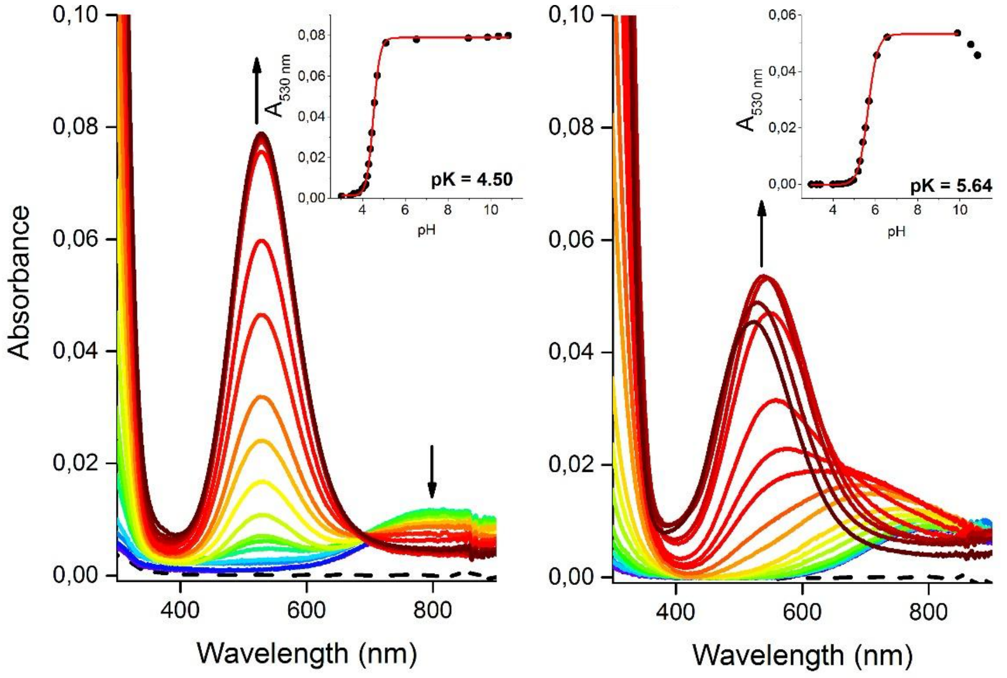

Figure S41. UV/VIS spectra of pH-metric titration of Cu(II) complexes of **a** (left) and **c** (right) in the range of 300–900 nm, rainbow color-coded from lowest (blue) to highest pH values (red). The black dashed lines represent peptides only. The insets show pH-dependent changes in absorption at 530 nm for **a** and **c**.

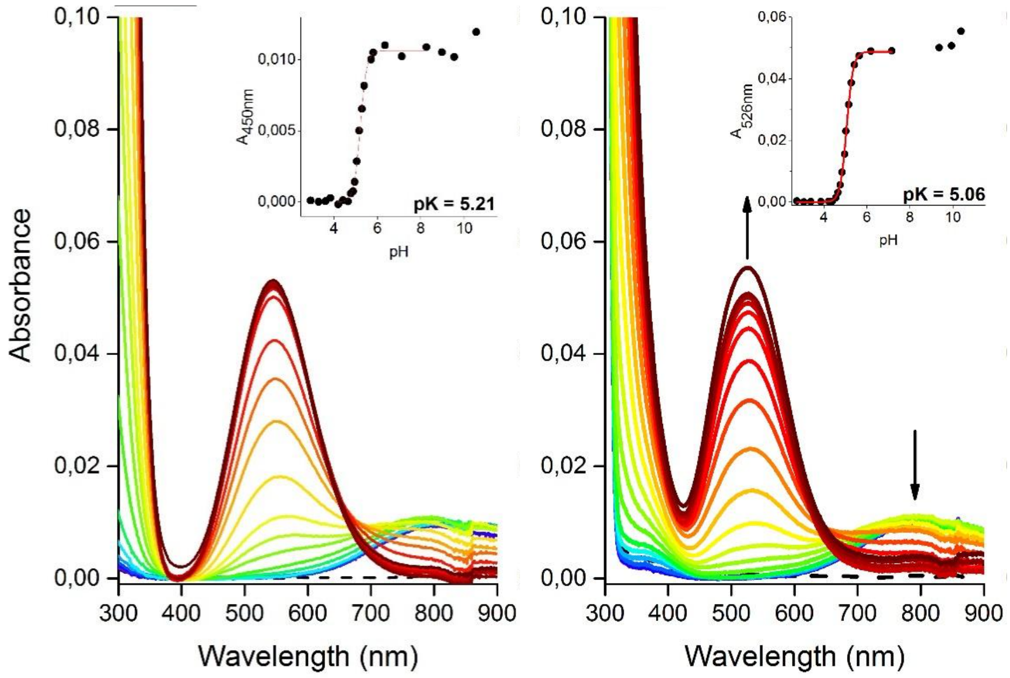

Figure S42. UV/VIS spectra of pH-metric titration of Cu(II) complexes of **d** (left) and **e** (right) in the range of 300–900 nm, rainbow color-coded from lowest (blue) to highest pH values (red). The black dashed lines represent peptides only. The insets show pH-dependent changes in absorption at 450 nm for **d** and 526 nm for **e**.

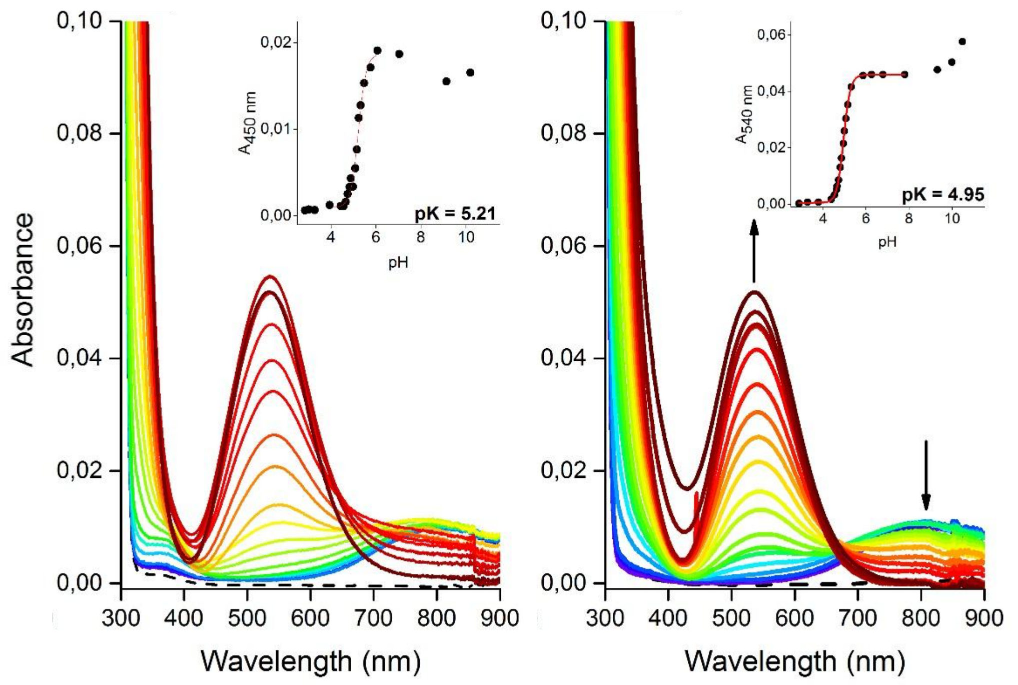

Figure S43. UV/VIS spectra of pH-metric titration of Cu(II) complexes of **f** (left) and **g** (right) in the range of 300–900 nm, rainbow color-coded from lowest (blue) to highest pH values (red). The black dashed lines represent peptides only. The insets show pH-dependent changes in absorption at 450 nm for **f** and 540 nm for **g**.

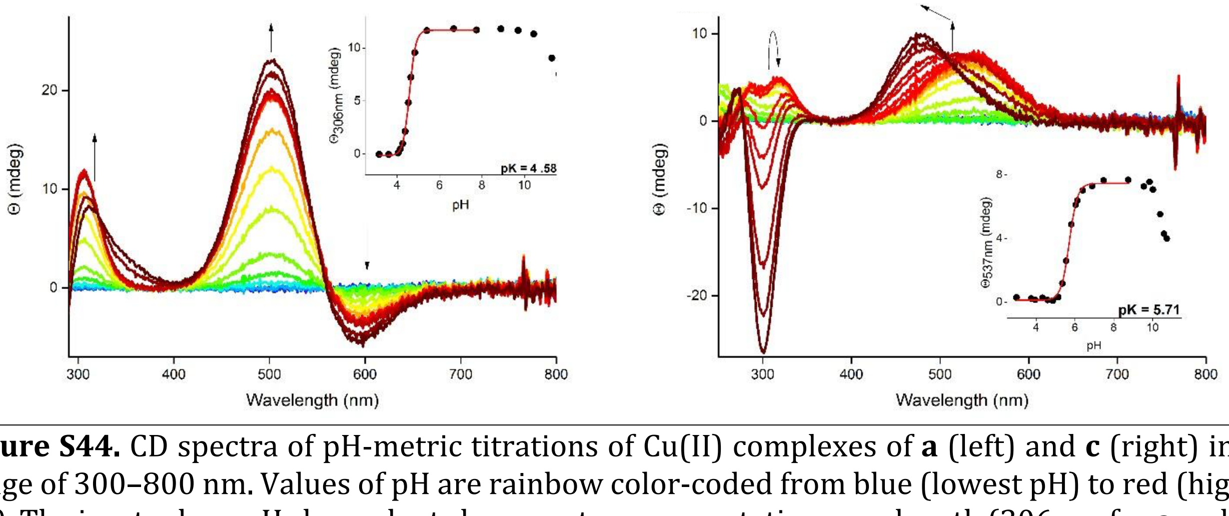

Figure S44. CD spectra of pH-metric titrations of Cu(II) complexes of **a** (left) and **c** (right) in the range of 300–800 nm. Values of pH are rainbow color-coded from blue (lowest pH) to red (highest pH). The insets show pH-dependent changes at a representative wavelength (306 nm for **a** and 537 nm for **c**).

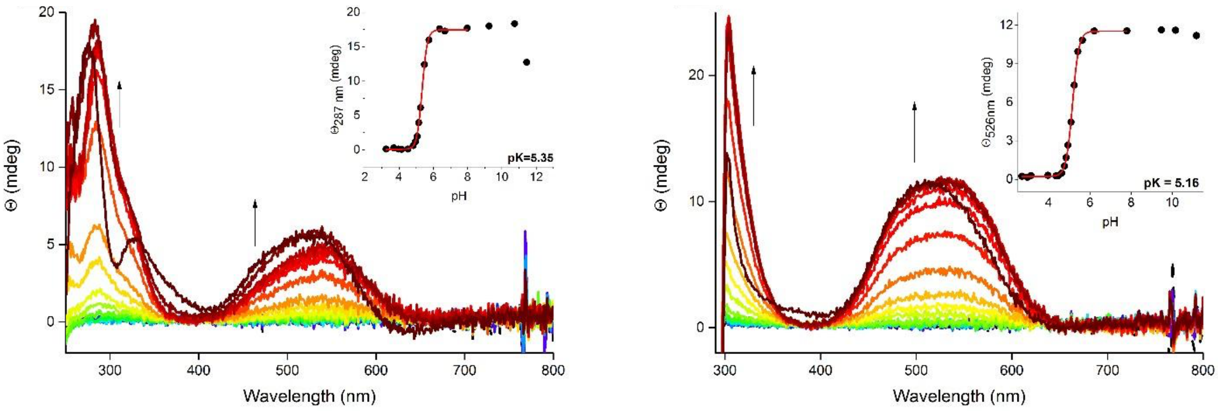

Figure S45. CD spectra of pH-metric titrations of Cu(II) complexes of **d** (left) and **e** (right) in the range of 300–800 nm. Values of pH are rainbow color-coded from blue (lowest pH) to red (highest pH). The insets show pH-dependent changes at a representative wavelength (287 nm for **d** and 526 nm for **e**).

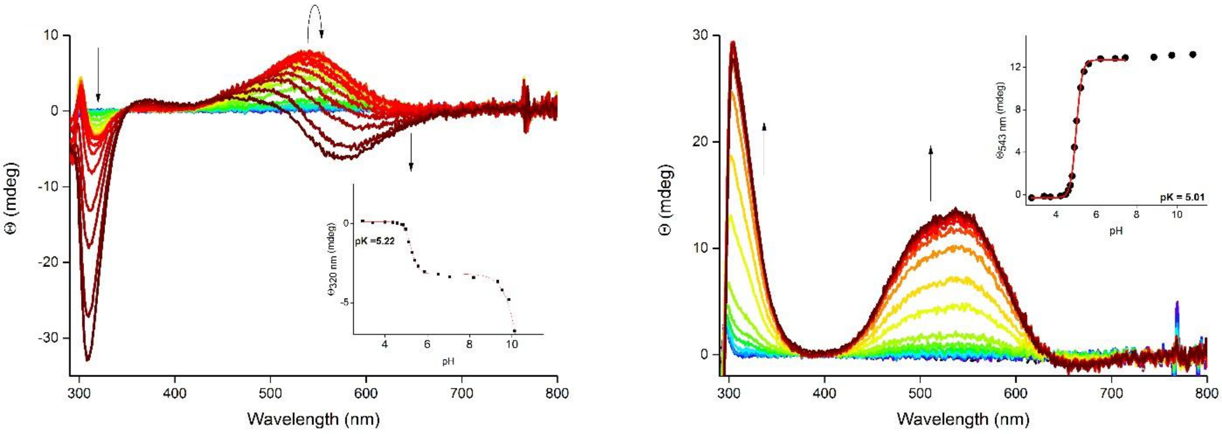

Figure S46. CD spectra of pH-metric titrations of Cu(II) complexes of **f** (left) and **g** (right) in the range of 300–800 nm. Values of pH are rainbow color-coded from blue (lowest pH) to red (highest pH). The insets show pH-dependent changes at a representative wavelength (320 nm for **f** and 543 nm for **g**).

S-5.3 UV/VIS titration of $[CuFz_2]^{3-}$ with peptides a and c

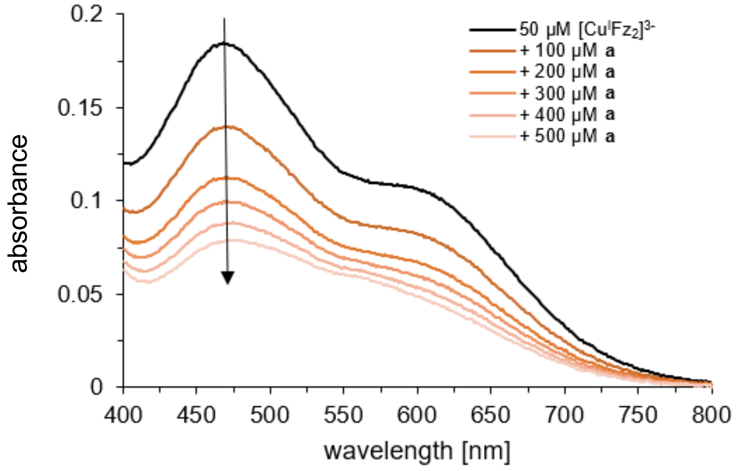

Figure S47. UV/VIS spectra of $[CuFz_2]^{3-}$ (50 µM, Cu(I):Fz ratio = 1:2.1) and titration of peptide **a** (100 to 500 µM) in HEPES (100 mM, pH 7.4) and 0.05% MeCN. The arrow indicates the decrease of the absorption band at 470 nm characteristic for the $[CuFz_2]^{3-}$ complex, which is used for calculation of K_{app} value for **a** (complex stability constants towards Cu(I)).

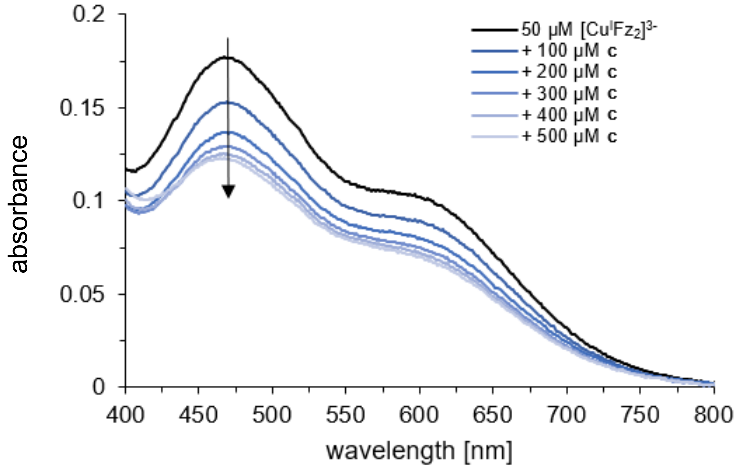

Figure S48. UV/VIS spectra of $[CuFz_2]^{3-}$ (50 µM, Cu(I):Fz ratio = 1:2.1) and titration of peptide **c** (100 to 500 µM) in HEPES (100 mM, pH 7.4) and 0.05% MeCN. The arrow indicates the decrease of the absorption band at 470 nm characteristic for the $[CuFz_2]^{3-}$ complex, which is used for calculation of the K_{app} value for **c** (complex stability constants towards Cu(I)).

Table S7. Complex stability constants of peptides **a** and **c** towards Cu(I) (K_{app}) with standard deviation calculated by competition with Fz and the association constant for $[Cu^I(Fz)_2]^{3-}$ (β = 3.7 x 10^{11} M^{-2}).

peptide	K_{app} [10^6 M^{-1}]
**a** (Gly2)	5.50 ± 0.92
**c** (β-Ala2)	1.78 ± 0.31

S-6 DNA cleavage studies

Plasmid DNA pBR322 was purchased from *Carl Roth*. All DNA cleavage experiments were performed at least in triplicate in order to ensure reproducibility. The standard deviations are represented as the error bars.

The *in situ* prepared complexes **1–7** (50 μM $CuCl_2$ / 62.5 μM ligand, Cu(II):peptide = 1:1.25, see S-4) and their corresponding ligands **a–g** (62.5 μM) were incubated with plasmid DNA pBR322 (0.025 mg/mL) buffered in MOPS (50 mM, pH 7.4) in the presence of ascorbate ($ascH^-$, 1 mM ascorbic acid) as reducing agent for 1 h at 37 °C. For comparison the Cu(II) peptide complex Cu-GGH was tested under the same conditions. Gel electrophoresis was carried out for 2 h at 40 V using a 1% agarose gel in 0.5X TBE buffer containing ethidium bromide (EtBr) (0.2 μg/mL). The bands of supercoiled (form I), open-circular/nicked (form II), and linear (Form III) DNA were visualized by fluorescence imaging of intercalating EtBr on a *Bio-Rad GelDoc EZ Imager*. Data analysis was performed with *Bio-Rad's Image Lab Software* (Version 3.0). Due to the decreased affinity of EtBr to supercoiled DNA a correction factor of 1.22 was used.[5]

For the ROS quenching assay the following ROS scavengers were added individually or together to the tested complex **4** under the same incubation conditions: DMSO (400 mM), NaN_3 (10 mM), pyruvic acid (2.5 mM) and SOD (625 U/mL). For pyruvate as ROS scavenger a MOPS concentration of 100 mM was needed to keep the pH value constant at 7.4.

S-6.1 Nuclease activity of peptides a–g in the presence of ascorbate as a reducing agent

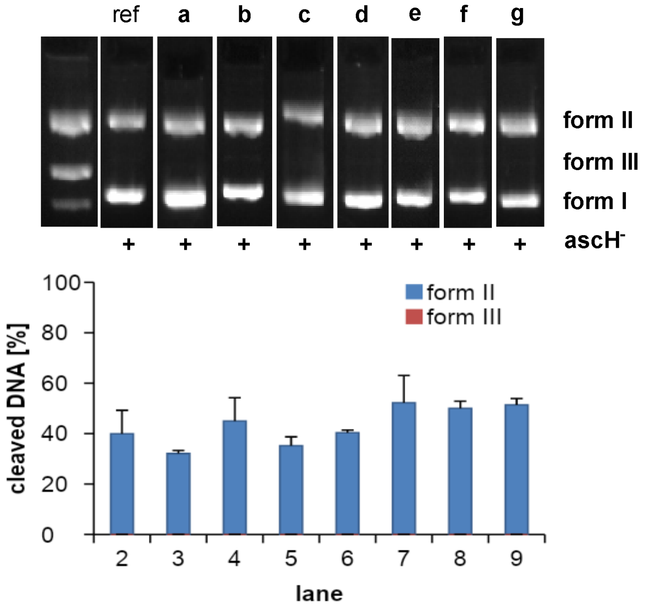

Figure S49. (Above) Nuclease activity towards plasmid DNA pBR322 (0.2 μg) of ATCUN peptides **a–g** (62.5 μM) in MOPS buffer (50 mM, pH 7.4) in the presence of $ascH^-$ (1 mM) as reducing agent after incubation for 1 h at 37 °C. Lane 1: DNA ladder (form I, II and III), lane 2: DNA reference, lanes 3-9: **a–g.** (Below) Visualization of the extent of DNA cleavage in percent.

Note: In this gel electrophoresis experiment with around 40% the amount of DNA form II in the reference was higher compared to other gel electrophoresis experiments (20–30%). This can be attributed to a DNA stock which had been stored over a longer period of time compared to the fresh stocks used otherwise. There is no influence to be expected for the outcome of this experiment, since no significant activity of peptides was observed.

S-6.2 Nuclease activity of Cu(II) ATCUN complex 4 at different concentrations in the presence of ascorbate as a reducing agent

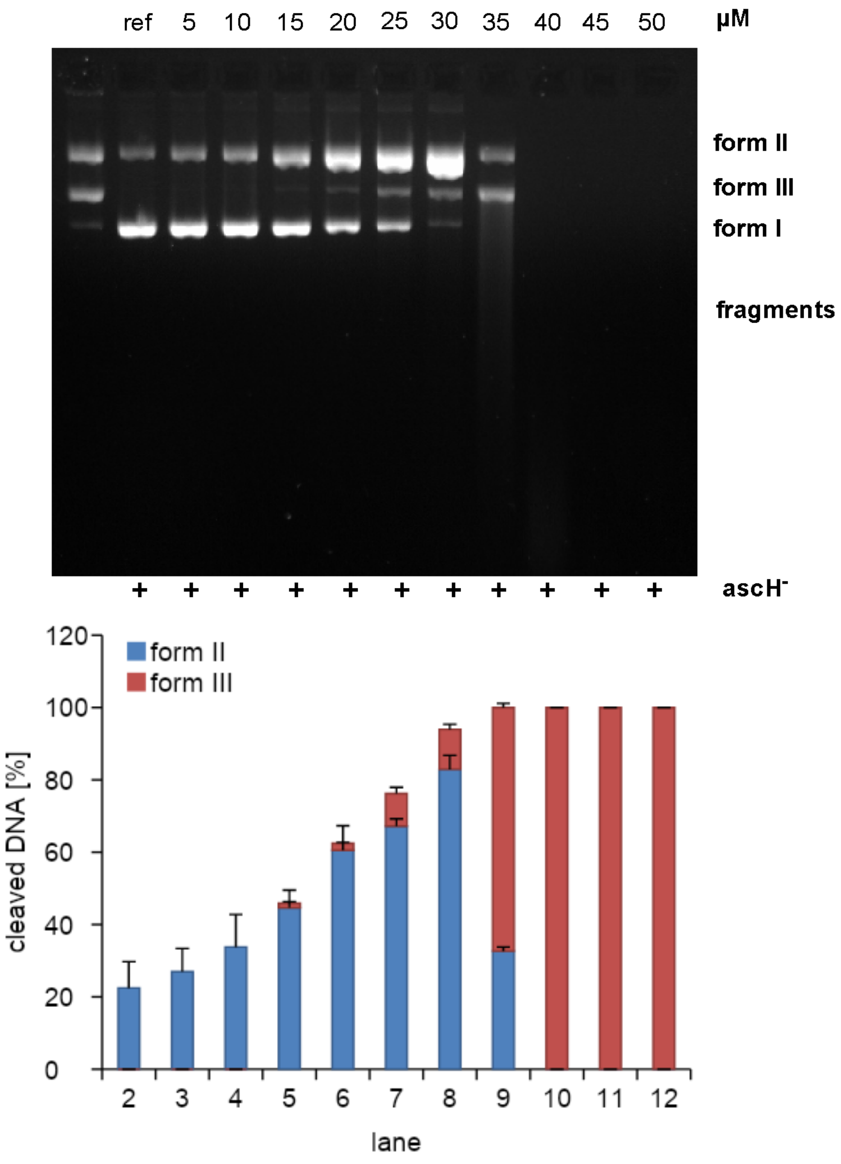

Figure S50. (Above) Nuclease activity of Cu(II) ATCUN complex **4** at different concentrations (5–50 µM) towards plasmid DNA pBR322 (0.2 µg) in MOPS buffer (50 mM, pH 7.4) in the presence of ascH$^-$ (1 mM) as reducing agent after incubation for 1 h at 37 °C. Lane 1: DNA ladder (form I, II and III), lane 2: DNA reference, lane 3-12: 5-50 µM. (Below) Visualization of the extent of DNA cleavage in percent.

S-7 DNA interaction studies

For DNA interaction investigations of the Cu(II) ATCUN complexes **1**-**7**, DNA melting studies (UV/VIS spectroscopy), EtBr displacement (fluorescence spectroscopy) and DNA-helicity/base stacking experiments (CD spectroscopy) were carried out.

S-7.1 DNA melting curves: UV/VIS spectroscopy

DNA melting curves of CT-DNA (50 µM) in MOPS buffer (50 mM, pH 7.4) in the presence of the *in situ* prepared Cu(II) ATCUN complexes **1**–**7** or $CuCl_2$ (2.5 µM) were measured at 260 nm using a heating rate of 0.5 °C/min. Four *Hellma* cuvettes with 1 mL sample volume each were used for carrying out the experiment simultaneously. Normalization of melting curves was utilized for better visualization.

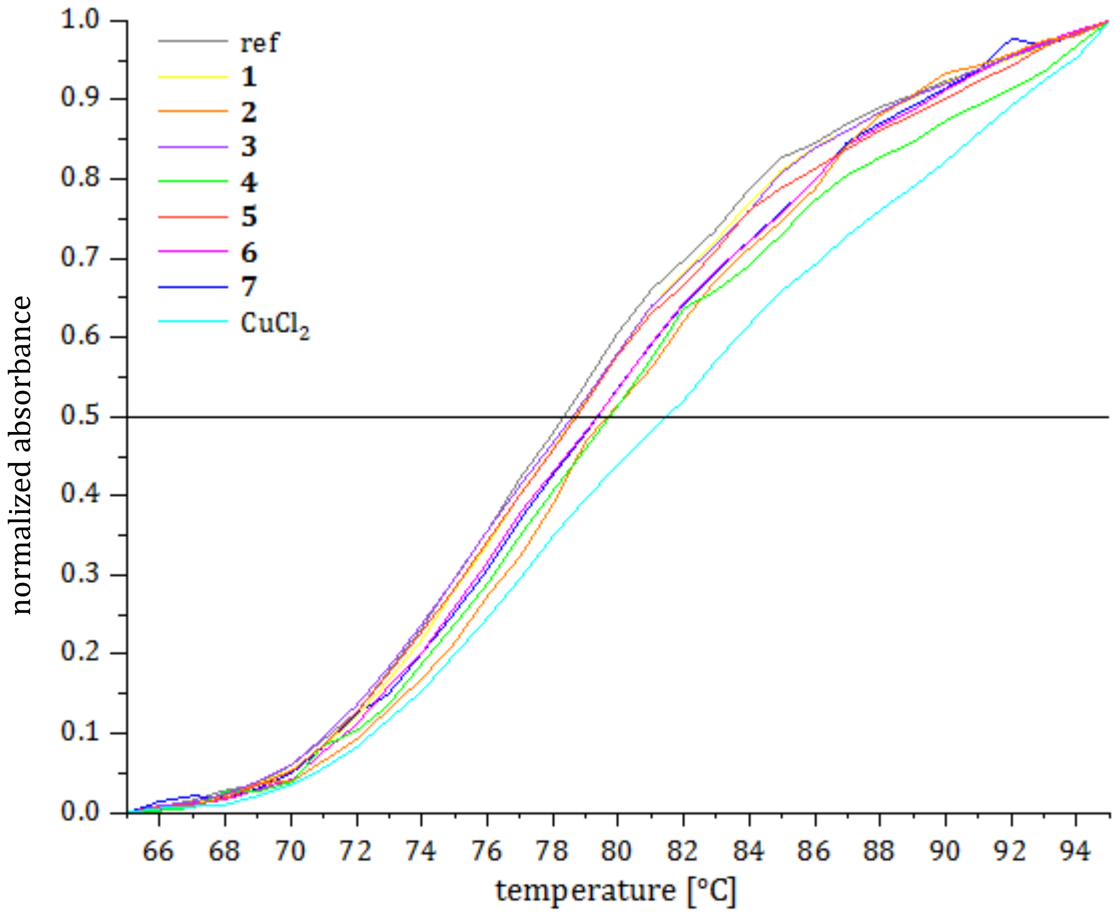

Figure S51. Normalized DNA melting curves in the presence of Cu(II) complexes **1**–**7** and $CuCl_2$.

In DNA melting experiments, the melting temperature T_m is the point where 50% of the double-stranded DNA is split into two single strands. Due different extinction coefficients at 260 nm for double-stranded DNA and two single DNA strands, T_m can be determined *via* UV/VIS spectroscopy (Figure S51).[6] Thereby, an increase of T_m (Table S8) by adding

complexes **1–7** (2.5 μM) to CT-DNA (50 μM) gives information about the DNA interaction strength.

Table S8. CT-DNA melting temperatures T_m after treatment with **1**-**7** (2.5 μM) and $CuCl_2$ and their change ΔT_m relative to CT-DNA (50 μM) in MOPS buffer (50 mM, pH 7.4).

compound	T_m [°C]	ΔT_m [°C]
CT-DNA (ref.)	78.3	0
+ **1**	78.7	0.4
+ **2**	79.7	1.4
+ **3**	78.6	0.3
+ **4**	79.8	1.5
+ **5**	78.7	0.4
+ **6**	79.3	1.0
+ **7**	79.4	1.1
+ $CuCl_2$	81.5	3.2

Treatment of CT-DNA with compounds **1–7** increased T_m up to 1.5 °C in comparison to the reference, indicating electrostatic interactions with DNA and/or DNA groove binding for all complexes.[7] In general, the substitution of Gly2 (**1** with ΔT_m of 0.4 °C) with β-Ala2 (**3** with ΔT_m of 0.3 °C) does not significantly affect the DNA affinity. Indeed, complexes **2** (Gly2; Lys1/4) with ΔT_m of 1.4 °C and **4** (β-Ala2; Lys1/4) with ΔT_m of 1.5 °C, each bearing two positively charged Lys (protonated amine groups) at pH 7.4, showed higher stabilization of the DNA double helix in comparison to their analogs **1** (Gly2) and **3** (β-Ala2) without Lys. Thus, the higher nuclease activity of Lys-bearing complexes **2** and **4** can be explained by their stronger electrostatic DNA interactions due to two additional positive charges (see Table S8, Figure 3: lanes 4–7). The T_m of complexes **6** (Lys1/Trp4) and **7** (Trp1/Lys4) are in between the complexes with Lys1/4-ATCUN and the ones with Gly1/4 ($\Delta T_m \approx 1$ °C). For complex **5** (Trp1/4), DNA intercalative properties due to the structural similarity to nucleobases and the planarity of indole in Trp were expected.[8-11] However, no significant affinity of complex **5** towards DNA beyond the general electrostatic interactions were observed. In **6** and **7**, the presence of another Lys[9] in the peptide sequence in addition to the Trp residue was not found to be beneficial to the affinity of the complexes to DNA.

S-7.2 Ethidium bromide (EtBr) displacement assay: fluorescence spectroscopy

A mixture of calf thymus (CT) DNA (20 μM) and EtBr (1.3 μM) in MOPS buffer (50 mM, pH 7.4) was prepared in a 1 mL fluorescence cuvette. After 15 min, this solution was treated with increasing amounts of the *in situ* prepared Cu(II) ATCUN complexes **1–7** and $CuCl_2$. The fluorescence spectra were collected after each addition, mixing and a waiting time of 1 min in a range of 550–700 nm using an excitation wavelength of 518 nm (photomultiplier voltage: 1000 V).

In the presence of DNA, EtBr emits intense fluorescence at λ_{em} = 603 nm due to intercalation of EtBr between the base pairs of the DNA. Competitive DNA binder molecules can displace EtBr causing a decreased fluorescence emission.[12] The magnitude of decrease of the fluorescence emission is described by the Stern-Volmer equation.[13]

$$\frac{I_0}{I} = 1 + K_{SV}[Q] \qquad (1)$$

where I_0 is the fluorescence emission in absence and I in presence of a competitive binder, and $[Q]$ is the concentration of the latter. Titration of a competitive quencher yields the Stern-Volmer constant K_{SV} by plotting its concentration $[Q]$ against I_0/I. In order to obtain the binding constant K_{app} towards DNA the equation

$$K_{EtBr}[EtBr] = K_{app}[Q] \qquad (2)$$

was used, where K_{EtBr} is the binding constant of EtBr towards DNA (10^7 M^{-1})[14] and $[EtBr]$ is the concentration of EtBr (1.3 µM). $[Q]$ is the concentration of the competitive binder, where the fluorescence emission of EtBr is quenched by 50%, which can be calculated by using equation (1).

The calculated Stern-Volmer constants K_{SV} and the apparent binding constants K_{app} for **1**–**7** are listed in Table S9. The EtBr displacement from the EtBr-CT-DNA system (1.3 µM EtBr, 20 µM CT-DNA) in MOPS buffer (50 mM, pH 7.4) by titration of the complexes **1–7** and $CuCl_2$ are shown in Figures S52–S59.

Table S9. Stern-Volmer constants K_{SV} and binding constants K_{app} towards CT-DNA for the Cu(II) ATCUN complexes **1–7** and $CuCl_2$.

Compound	K_{SV} [M^{-1}]	K_{app} [M^{-1}]
**1**	2.07×10^3	2.67×10^4
**2**	9.30×10^3	1.21×10^5
**3**	2.36×10^3	3.07×10^4
**4**	9.01×10^3	1.17×10^5
**5**	2.52×10^3	3.28×10^4
**6**	3.57×10^3	4.64×10^4
**7**	3.68×10^3	4.78×10^4
$CuCl_2$	1.88×10^4	2.45×10^5

The results of the EtBr displacement assay showed the same trend as the DNA melting experiments. The fluorescence of the intercalated EtBr was quenched at a higher degree in the case of β-Ala2 complexes with positively charged Lys residues such as **4** (Lys1/Lys4) compared to **3** (Gly1/Gly4) (Figure S55 and S54). Furthermore, the calculated K_{app} values of complexes **1**-**7** indicate electrostatic interaction and/or groove binding as dominant DNA binding modes for all complexes (K_{app} < 10^6 M^{-1}).[7,15] In both, the DNA melting and EtBr displacement experiments, $CuCl_2$ showed a pronounced DNA binding. It can be assumed that the complex $[Cu(H_2O)_6]^{2+}$ formed from $CuCl_2$ in aqueous

solution can more easily approach the DNA in comparison to the ATCUN complexes with their bigger and thus shielding peptidic ligand systems. Furthermore, fluorescence quenching by Cu(II) could be responsible for the decreased fluorescence from EtBr signal in comparison to the complexes.[16,17]

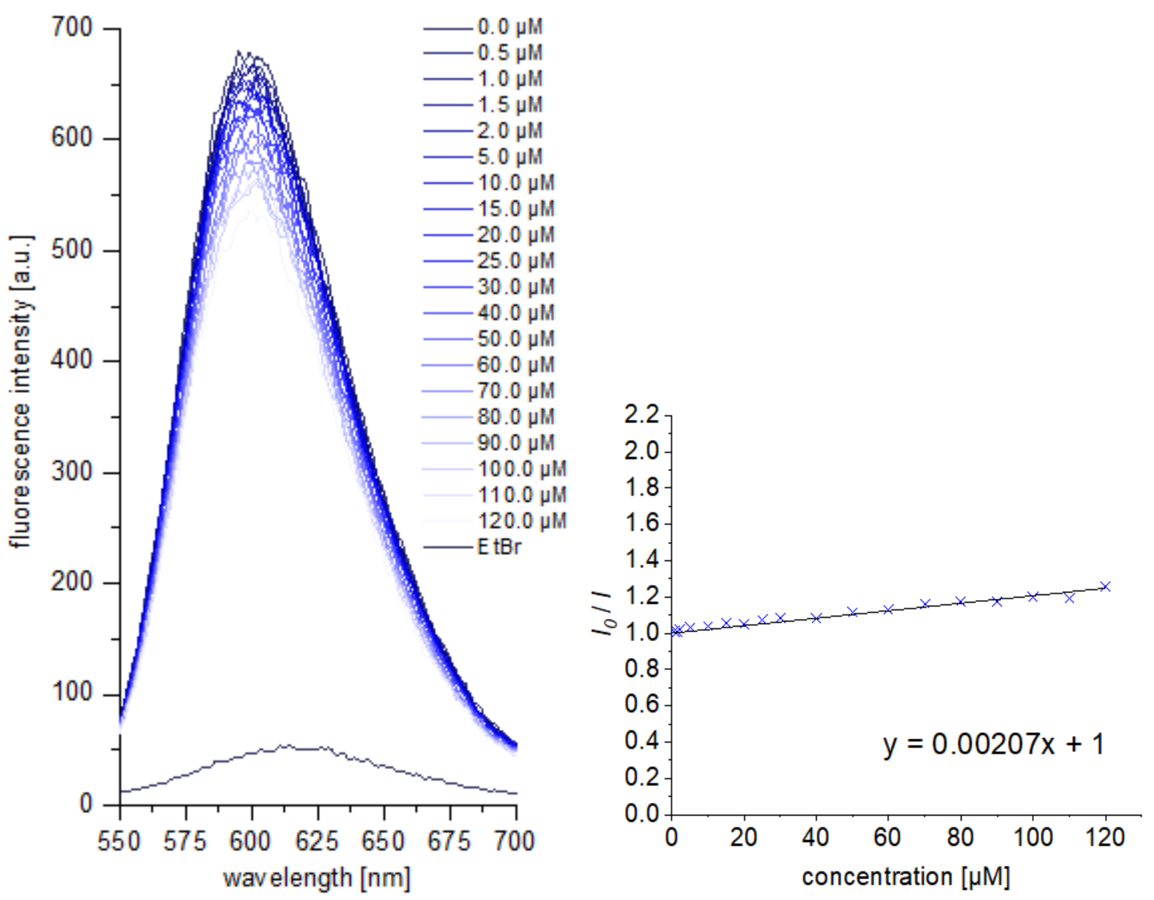

Figure S52. (Left) EtBr displacement of the EtBr-CT-DNA system (1.3 µM EtBr, 20 µM CT-DNA) in MOPS buffer (50 mM, pH 7.4) by titration of the Cu(II) ATCUN complex **1** (0–120 µM). (Right) I_0/I over $[Q]$ to calculate K_{SV}.

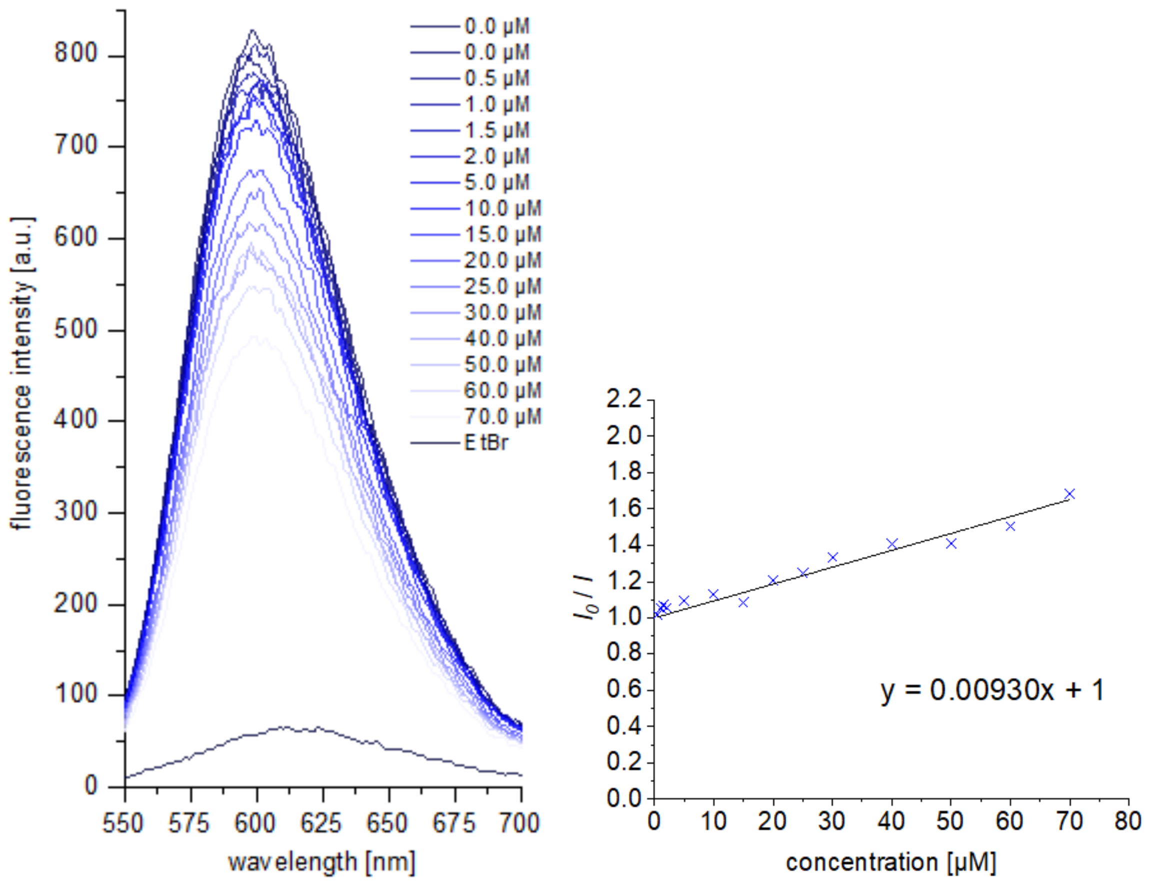

Figure S53. (Left) EtBr displacement of the EtBr-CT-DNA system (1.3 µM EtBr, 20 µM CT-DNA) in MOPS buffer (50 mM, pH 7.4) by titration of the Cu(II) ATCUN complex **2** (0–70 µM). (Right) I_0/I over $[Q]$ to calculate K_{SV}.

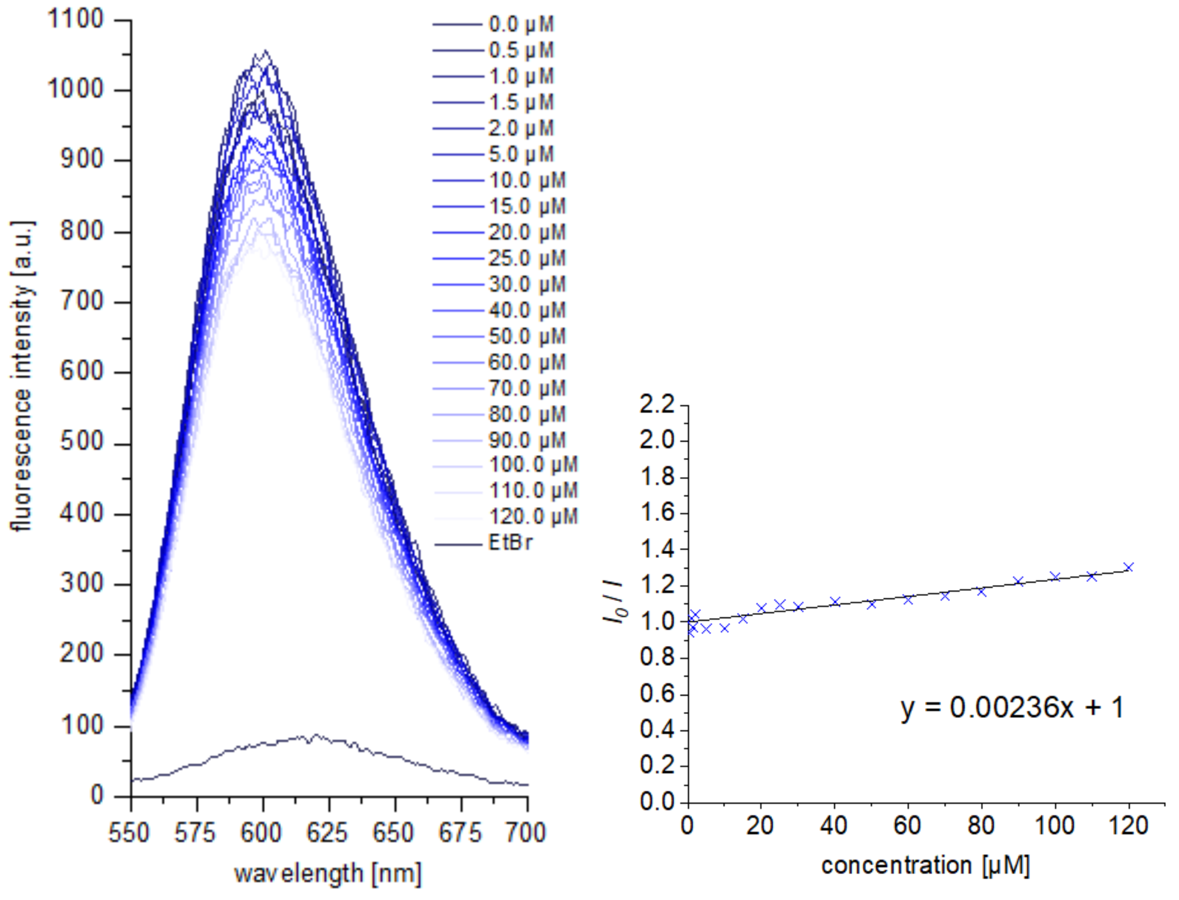

Figure S54. (Left) EtBr displacement of the EtBr-CT-DNA system (1.3 µM EtBr, 20 µM CT-DNA) in MOPS buffer (50 mM, pH 7.4) by titration of the Cu(II) ATCUN complex **3** (0–120 µM). (Right) I_0/I over $[Q]$ to calculate K_{SV}.

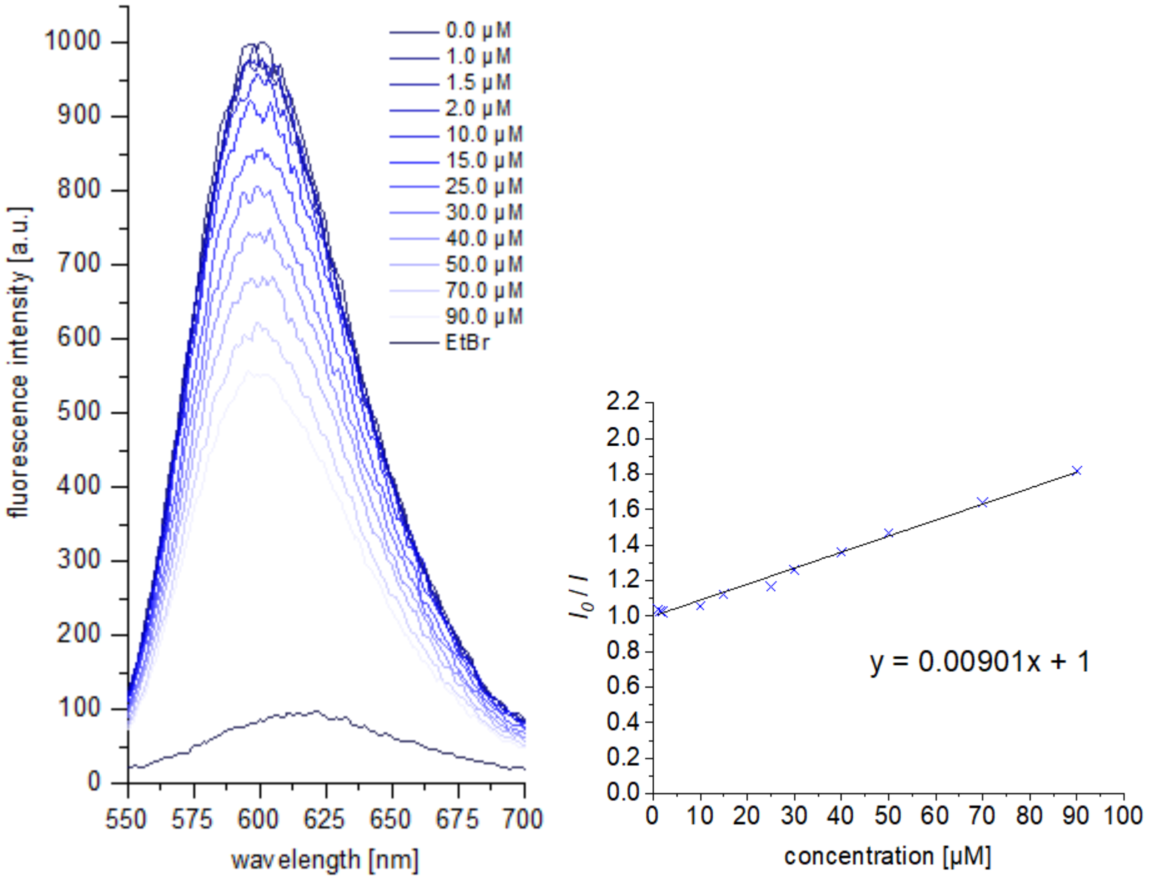

Figure S55. (Left) EtBr displacement of the EtBr-CT-DNA system (1.3 µM EtBr, 20 µM CT-DNA) in MOPS buffer (50 mM, pH 7.4) by titration of the Cu(II) ATCUN complex **4** (0–90 µM). (Right) I_0/I over [Q] to calculate K_{SV}.

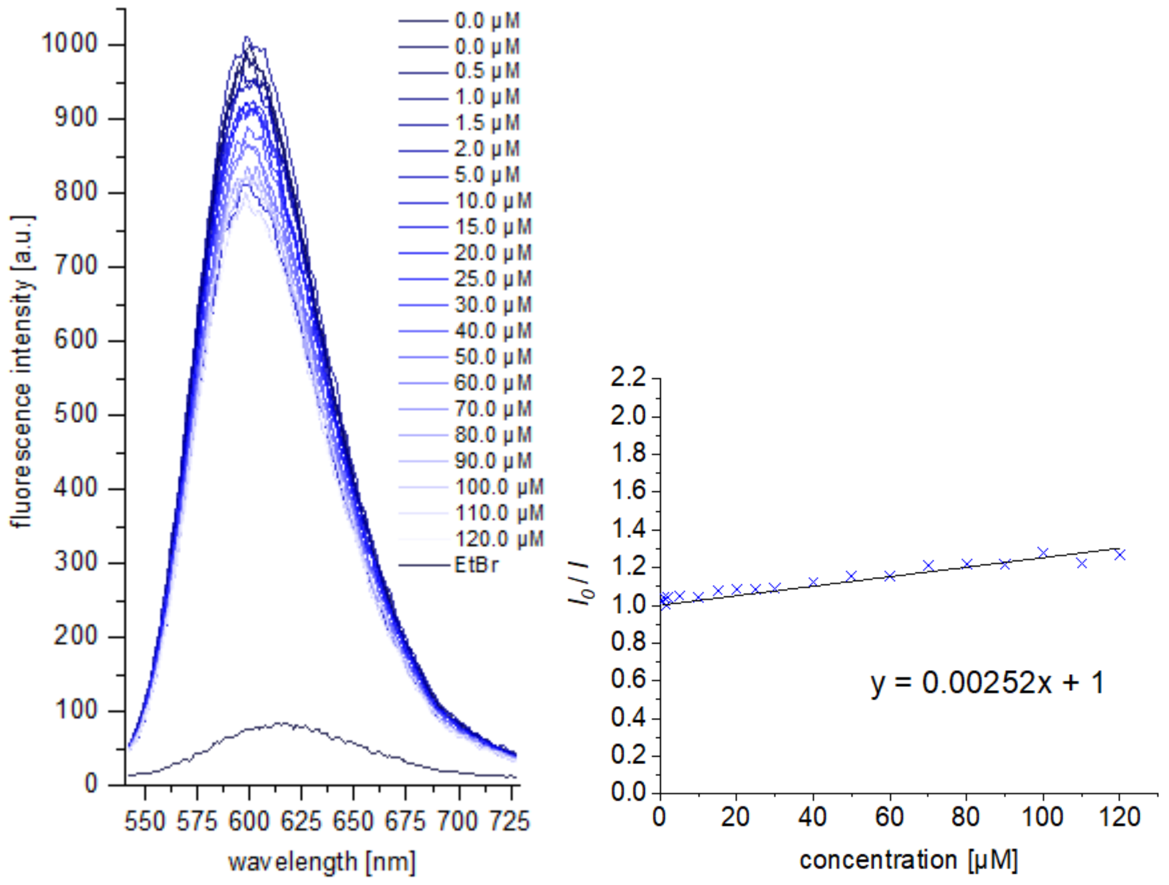

Figure S56. (Left) EtBr displacement of the EtBr-CT-DNA system (1.3 µM EtBr, 20 µM CT-DNA) in MOPS buffer (50 mM, pH 7.4) by titration of the Cu(II) ATCUN complex **5** (0–120 µM). (Right) I_0/I over $[Q]$ to calculate K_{SV}.

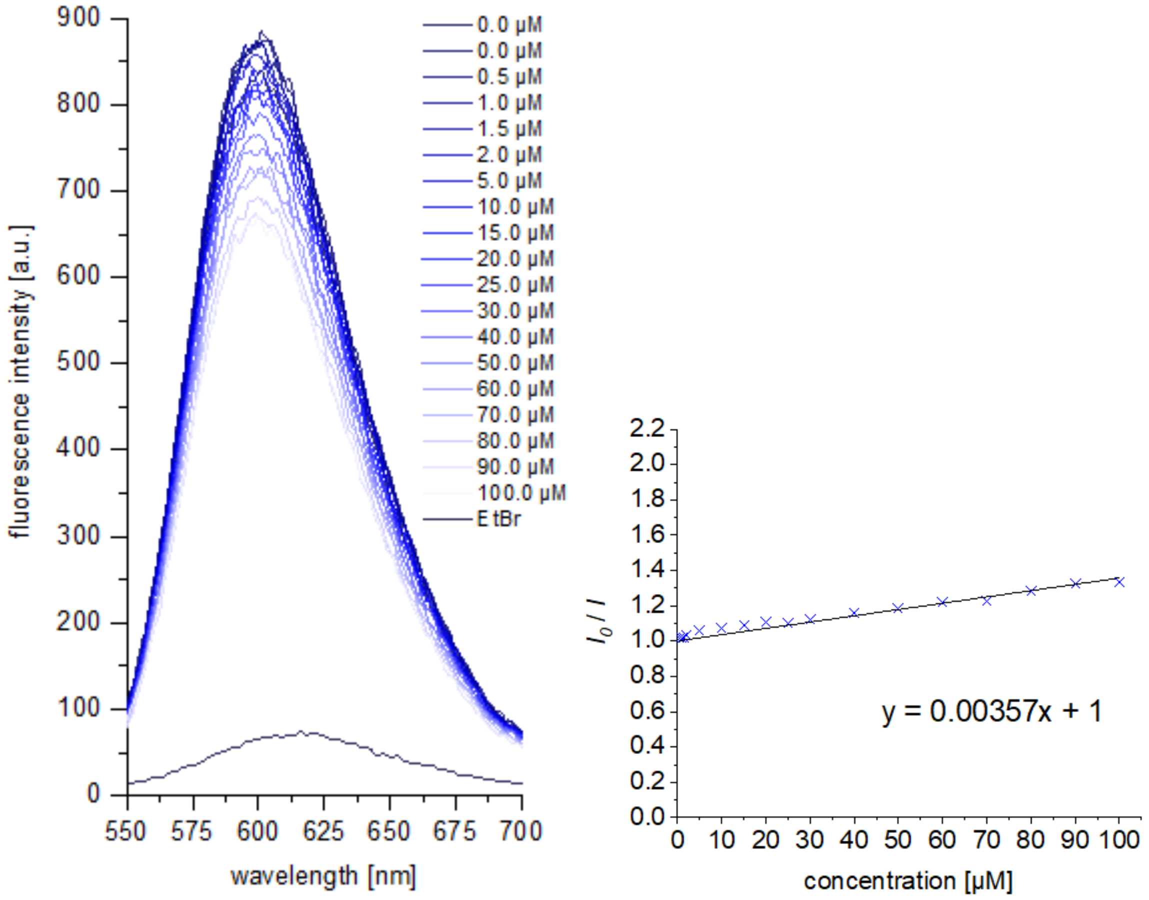

Figure S57. (Left) EtBr displacement of the EtBr-CT-DNA system (1.3 µM EtBr, 20 µM CT-DNA) in MOPS buffer (50 mM, pH 7.4) by titration of the Cu(II) ATCUN complex **6** (0–100 µM). (Right) I_0/I over $[Q]$ to calculate K_{SV}.

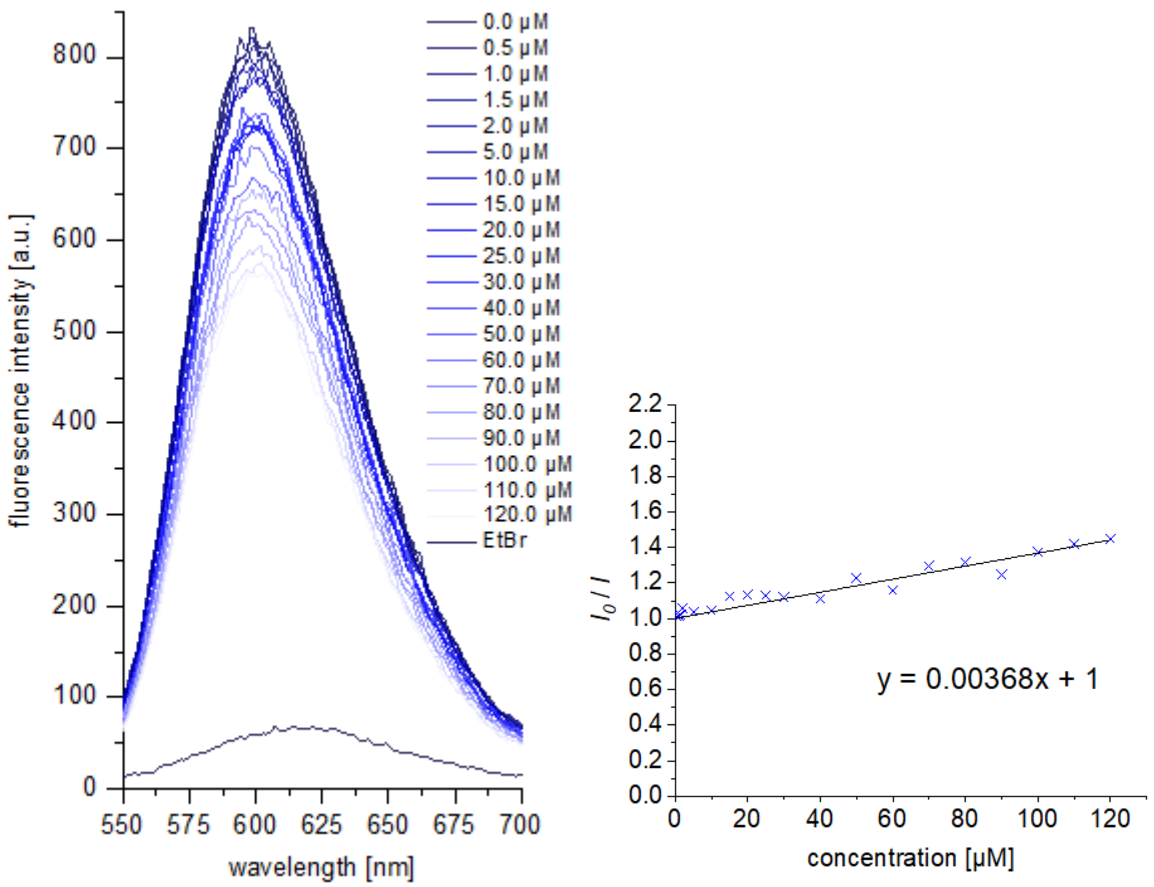

Figure S58. (Left) EtBr displacement of the EtBr-CT-DNA system (1.3 μM EtBr, 20 μM CT-DNA) in MOPS buffer (50 mM, pH 7.4) by titration of the Cu(II) ATCUN complex **7** (0–120 μM). (Right) I_0/I over $[Q]$ to calculate K_{SV}.

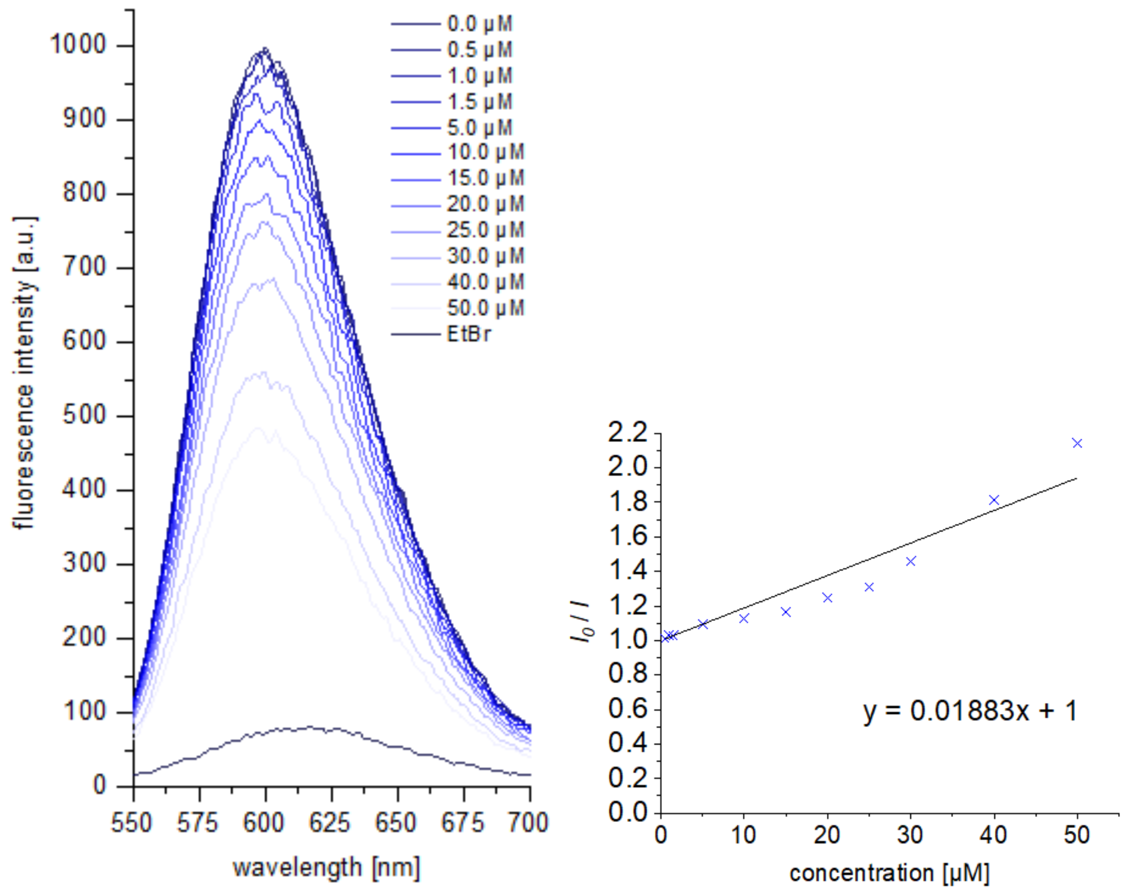

Figure S59. (Left) EtBr displacement of the EtBr-CT-DNA system (1.3 µM EtBr, 20 µM CT-DNA) in MOPS buffer (50 mM, pH 7.4) by titration of $CuCl_2$ (0–50 µM). (Right) I_0/I over $[Q]$ to calculate K_{SV}.

S-7.3 CD spectroscopy

CD spectra of CT DNA (100 µM) in MOPS buffer (50 mM, pH 7.4) were recorded in a range of 220 to 320 nm with a scan rate of 100 nm/min and a data point interval of 0.1 nm. The Cu(II) ATCUN complexes **1-7** (10 µM $CuCl_2$ / 10.5 µM peptide to 30 µM $CuCl_2$ / 31.5 µM peptide) or $CuCl_2$ (10–30 µM) were added stepwise to investigate their modes of interaction with DNA. The volume of all samples was adjusted to 1 mL.

CD spectroscopy is a convenient method that can be used for a better understanding of groove binding and electrostatic interactions of the complexes towards DNA. CT-DNA exhibits a positive band at around 275 nm in the CD spectrum due to base-stacking of nucleobases and a negative band at around 245 nm, which corresponds to the helicity of the DNA (B-form).[18] Significant alteration of the positive band indicates intercalation, while changes of the negative band hint to groove binding interactions.[19–21]
Complexes **1-7** and $CuCl_2$ were titrated (0–30 µM) to a MOPS buffered (50 mM, pH 7.4) CT-DNA solution (100 µM). In Figures S60–S63 the CD spectra of CT-DNA alone and in the presence of different concentrations of complexes **1–7** and $CuCl_2$ are shown.

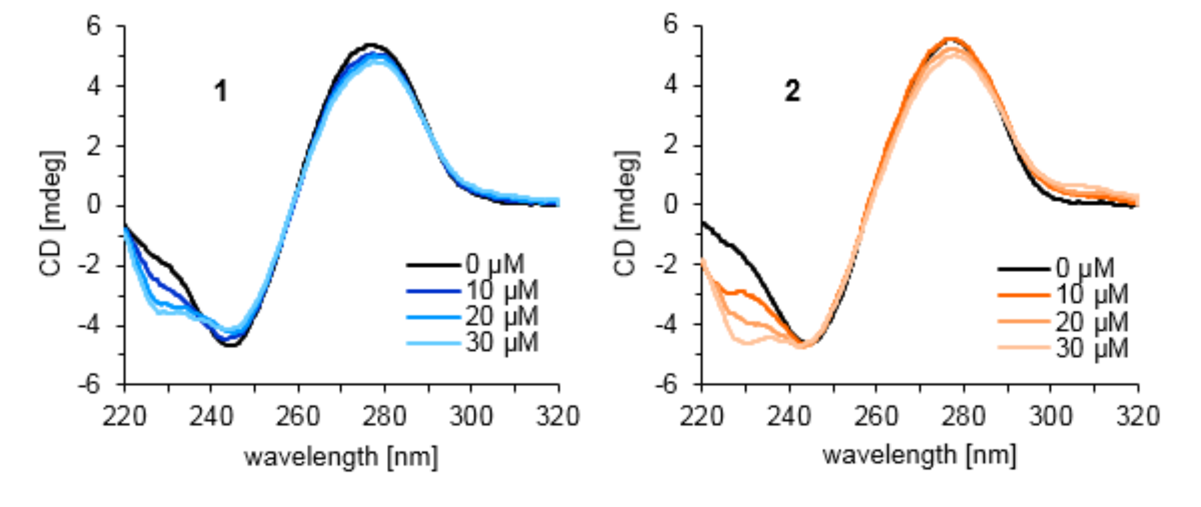

Figure S60. CD spectra of CT-DNA (100 μM) in MOPS buffer (50 mM, pH 7.4) with increasing concentrations (0–30 μM) of **1** (left) and **2** (right).

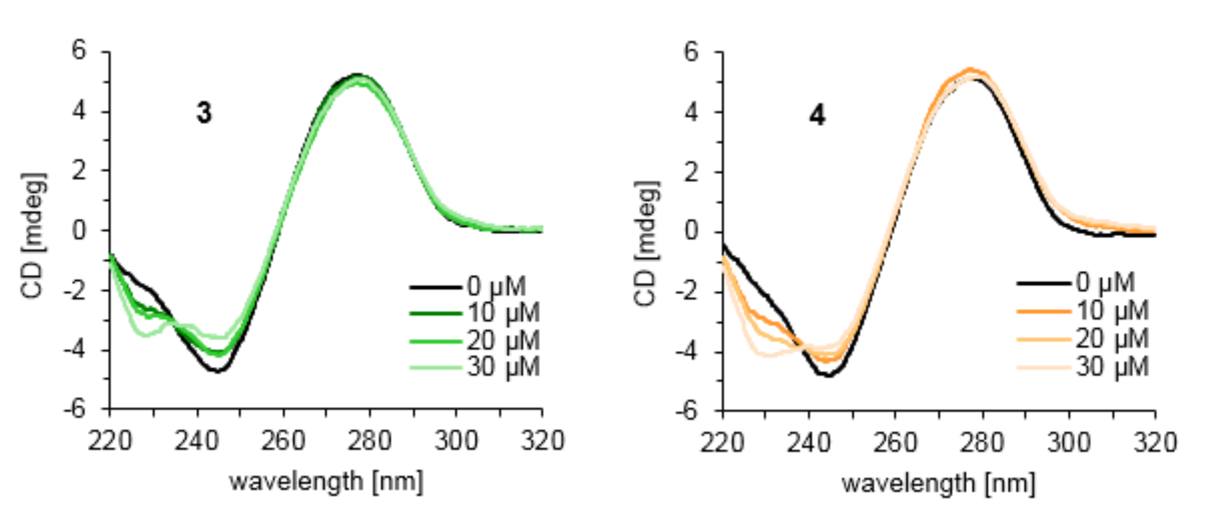

Figure S61. CD spectra of CT-DNA (100 μM) in MOPS buffer (50 mM, pH 7.4) with increasing concentrations (0–30 μM) of **3** (left) and **4** (right).

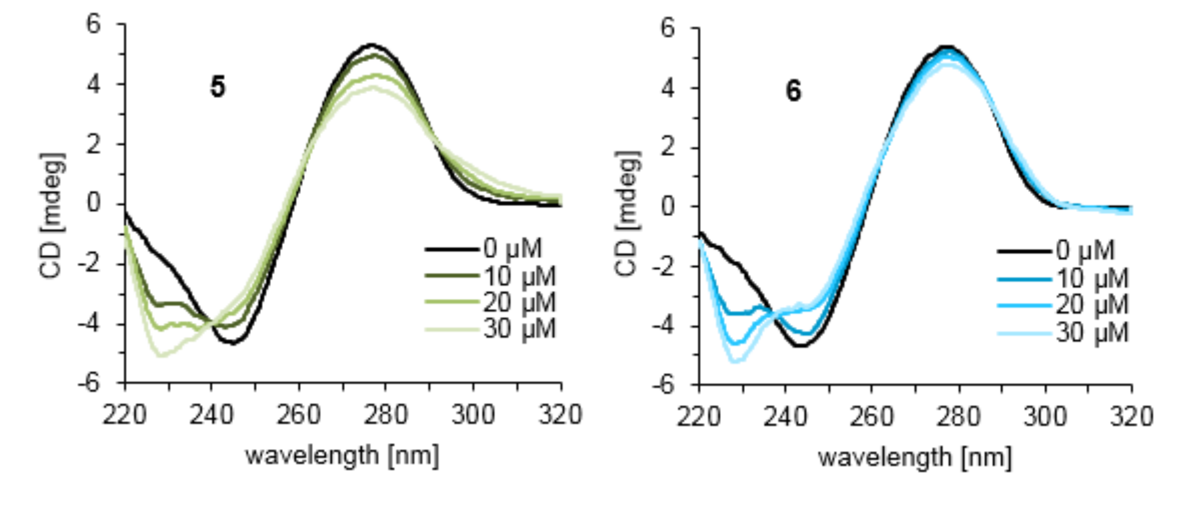

Figure S62. CD spectra of CT-DNA (100 μM) in MOPS buffer (50 mM, pH 7.4) with increasing concentrations (0–30 μM) of **5** (left) and **6** (right).

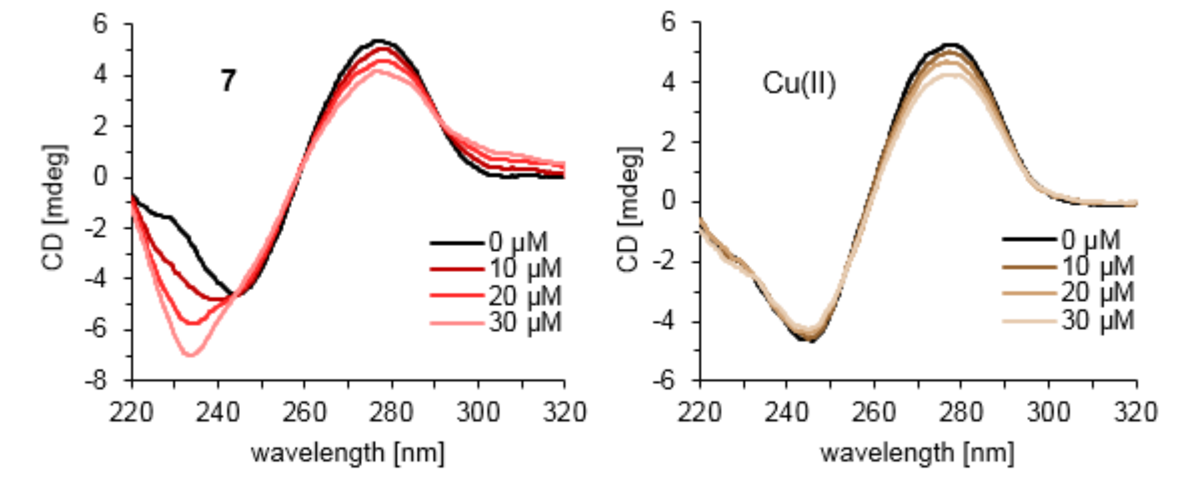

Figure S63. CD spectra of CT-DNA (100 μM) in MOPS buffer (50 mM, pH 7.4) with increasing concentrations (0–30 μM) of **7** (left) and $CuCl_2$ (right).

All complexes **1–7** in concentrations up to 30 μM caused only moderate alterations in the negative and positive band of CT-DNA. This strongly supports electrostatic interactions as the dominant DNA binding mode as also derived from UV/VIS and fluorescence spectroscopy measurements. Incorporation of Trp (**5**, **6** and **7**) leads to more pronounced changes in the negative band (Figures S62 and S63 left). Moreover, Trp residues in close proximity to DNA grooves may interact with nucleobases *via* hydrogen bridging or T-shaped interactions of the aromatic systems, resulting in a pronounced helicity alteration.[22] Indeed, a partial groove binding character can be suggested for **5–7** due to small changes in the helicity of CT-DNA.[19–21] In contrast, Lys incorporation (**2** and **4**) does not affect DNA helicity (Figures S60 and S61).

S-8 Detection of hydroxyl radicals and hydrogen peroxide

For the identification of generated ROS by Cu(II) ATCUN complexes during DNA cleavage, gel electrophoresis was used for a ROS quenching experiment (Figure 4). Detection of hydroxyl radicals and hydrogen peroxide generated by metallonucleases was additionally performed by fluorescence spectroscopy.

The fluorescent dyes disodium terephthalate (TPA) and pentafluorobenzenesulfonyl fluorescein (PBSF) were used for the detection of hydroxyl radicals and hydrogen peroxide, respectively. For the fluorescence experiments, the Cu(II) ATCUN complexes **1**, **3** or **4** or $CuCl_2$ (40 µM) were incubated in MOPS buffer (50 mM, pH 7.4) and either with TPA (0.5 mM) for hydroxyl radicals or with PBSF (25 µM) for hydrogen peroxide and ascorbic acid (1 mM) for 3.5 h at room temperature (r.t.). DMSO (200 mM) or pyruvic acid (2 mM) were used as ROS scavengers for hydroxyl radicals and hydrogen peroxide, respectively.
The components were pipetted into Eppendorf tubes in the following order: Milli-Q® water, buffer, fluorescent dye, ROS scavenger, complex, reducing agent. The fluorescence spectra were measured in the emission scan mode (slit width 5 nm, excitation: λ_{ex} = 320 nm for TPA, λ_{ex} = 485 nm for PBSF, photomultiplier voltage: 875 V for TPA and 695 V for PBSF) using quartz cells from *Hellma Analytics*.
In additional experiments, all components (Cu(II) complex, reducing agent, scavenger) were measured individually in buffered fluorescent dye (TPA or PBSF) solution in order to ensure that any signal observed is generated only by reduction of the Cu(II) complexes (Figures S64 and S65).

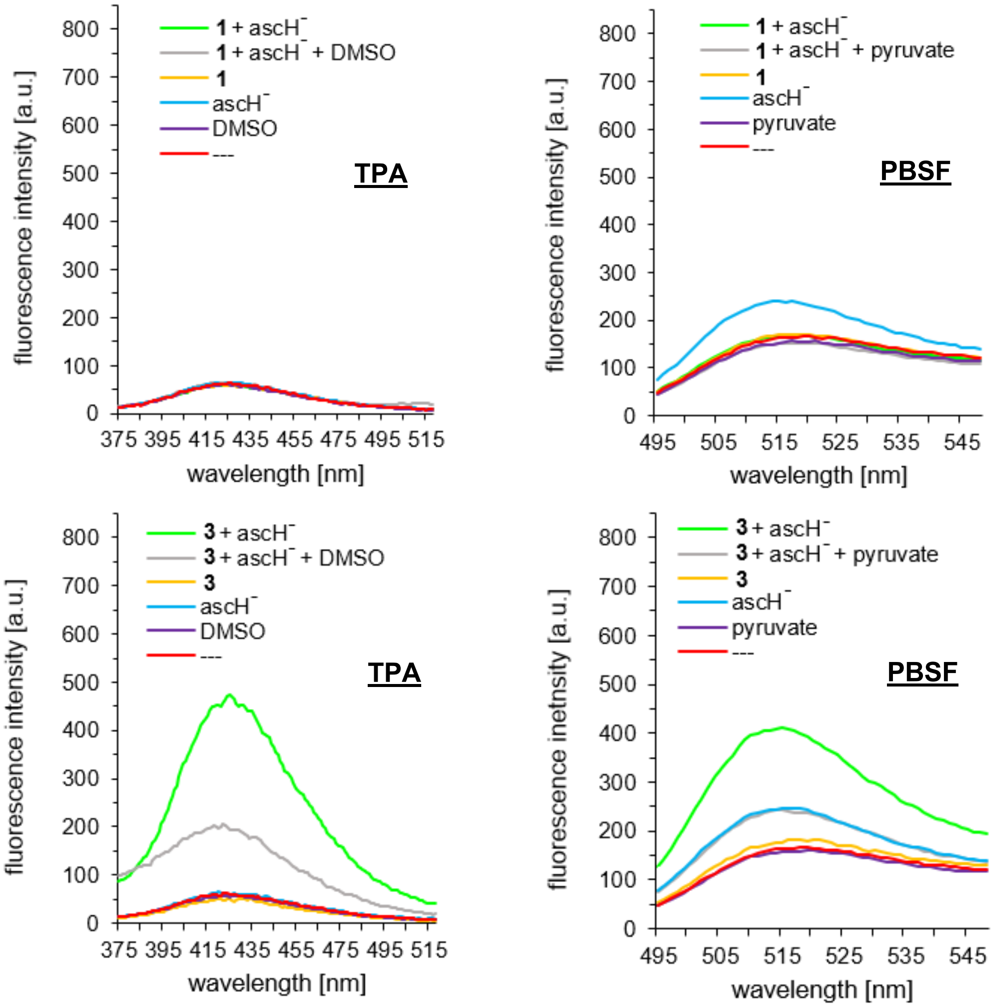

Figure S64. Fluorescence spectra of TPA (0.5 mM) and PBSF (25 µM) after incubation with Cu(II) ATCUN complex **1** (40 µM) (top) and **3** (40 µM) (bottom), reducing agent $ascH^-$ (1mM) and with or without the corresponding ROS scavenger DMSO (200 mM) and pyruvate (2.5 mM), respectively, in MOPS buffer (50 mM, pH 7.4) for 3.5 h at r.t. All individual components (complex **1**/**3**, $ascH^-$, DMSO/pyruvate) were analyzed separately with TPA/PBSF under the same conditions. One sample contained only TPA/PBSF (---).

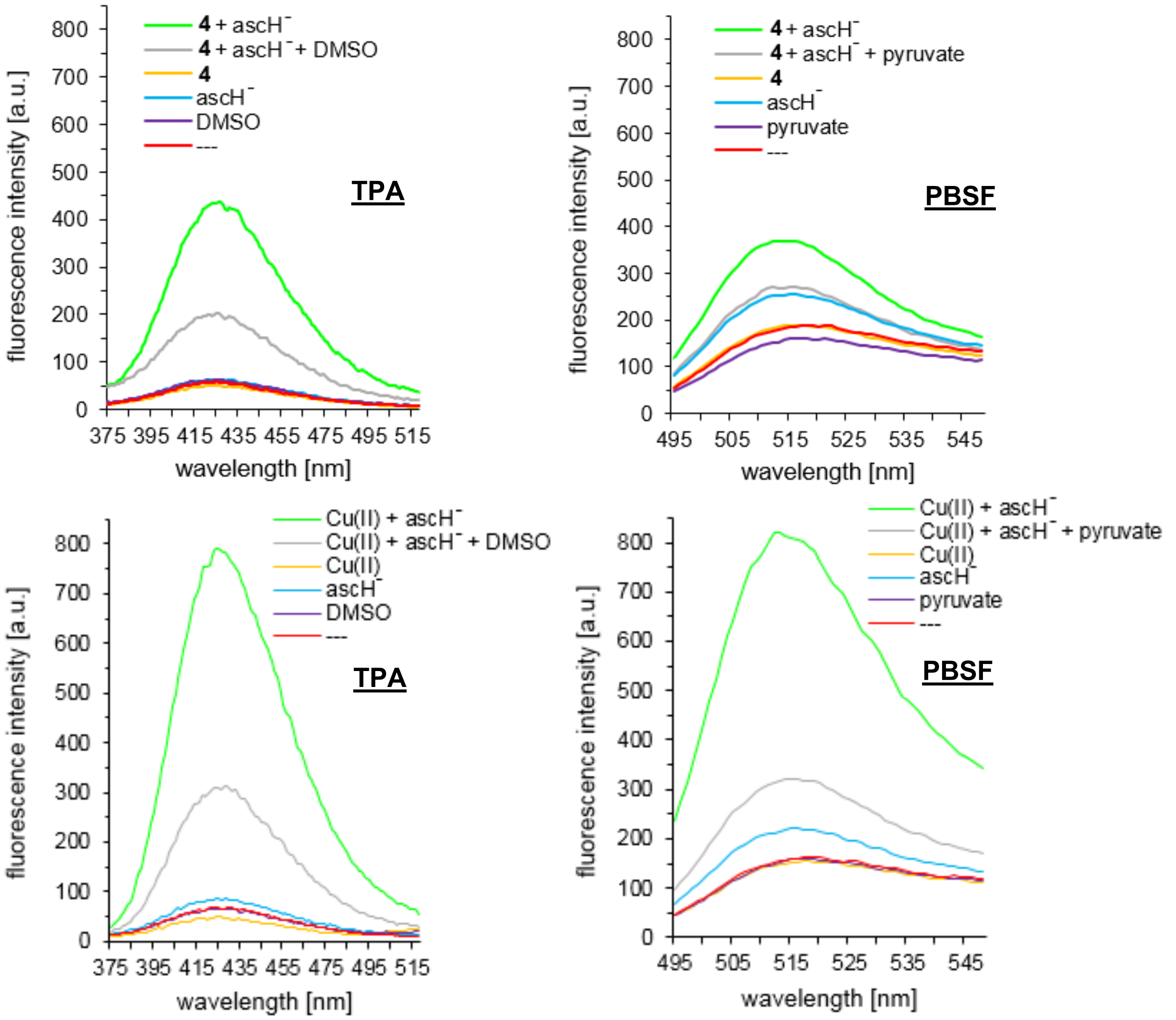

Figure S65. Fluorescence spectra of TPA (0.5 mM) and PBSF (25 µM) after incubation with Cu(II) ATCUN complex **4** (40µM) (top) and $CuCl_2$ (40 µM) (bottom), reducing agent $ascH^-$ (1mM) and with or without the corresponding ROS scavenger DMSO (200 mM) and pyruvate (2.5 mM), respectively, in MOPS buffer (50 mM, pH 7.4) for 3.5 h at r.t. All individual components (complex **4**/$CuCl_2$, $ascH^-$, DMSO/pyruvate) were analyzed separately with TPA/PBSF under the same conditions. One sample contained only TPA/PBSF (---).

Neither of the individual components (Cu(II) complex/salt, $ascH^-$, scavenger or fluorogenic compound) showed significant fluorescence signals (Figures S64 and S65). The corresponding ROS ($\cdot OH$ and H_2O_2) were generated by the metallonucleases in a reducing environment in the case of the β-Ala2 complexes. For the Gly2 complex **1,** no $\cdot OH$ and H_2O_2 production was induced in the presence of $ascH^-$. Furthermore, comparison of the background fluorescence of PBSF after 3.5 h (Figure S64, right, red line) with the kinetics of the signal evolution in case of Gly2-ATCUN complex **1** (Figure 5, right, red line) revealed that the signal results from auto-perhydrolysis of PBSF at r.t. (PBSF is stored at -24 °C) rather than being induced by **1** itself.

S-9 Cyclic voltammetry

Cyclic voltammograms were recorded with a *μStat 400 Bipotentiostat/Galvanostat* (*Metrohm DropSens*) by working in a 96 mM KNO_3/4 mM HNO_3 aqueous solution at pH 7.4. Concentrations of the Cu(II) complexes were about 0.5 mM (Cu(II):peptide = 1:1.1), and the pH was adjusted immediately before the measurement.

A *Screen-Printed Carbon electrode* (*Metrohm DropSens*) was used where the electrochemical cell consists of carbon working and auxiliary electrodes and silver as the reference electrode on a ceramic substrate. The scan rate was 100 mV/s. The *Metrohm DropView 8400* software was used for measurements, graph plots and data analysis.

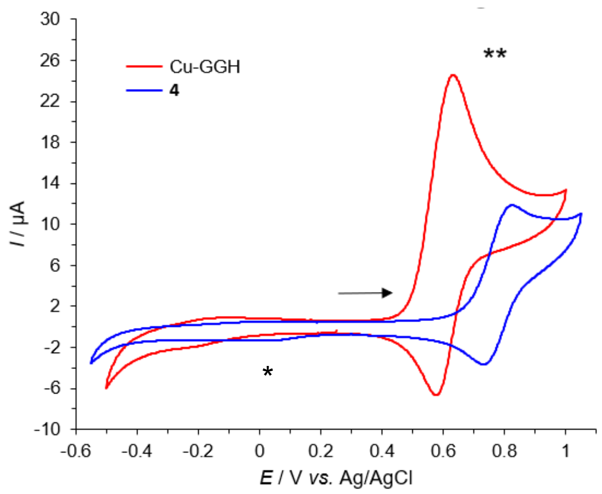

Figure S66. Cyclic voltammograms obtained for 0.5 mM of complexes Cu-GGH (Gly2; 5,5,6) and **4** (β-Ala2; 5,6,6) at pH 7.4 recorded in 96 mM KNO_3/4 mM HNO_3. A minor Cu(II)/Cu(I) reduction wave is visible for **4** (*). A quasi-reversible Cu(II)/Cu(III) oxidation process was observed for both Cu-GGH and **4** (**). The arrow indicates the starting point and direction of the potential. The scan rate was 100 mV/s.

S-10 EPR spectroscopy

Q-band (33.9 GHz) CW-EPR measurements were conducted on a *Bruker EMX-plusQ* spectrometer, using a standard *ER5106QT* resonator. The temperature was varied from 150 K to 20 K to check relaxation-dependent behavior of the paramagnetic species (Figure S67 and S68). A closed cycle cryostat (*Sumitomo cryo compressor-F70*) was used for cooling together with a temperature controller (*Mercury iTC*, *Oxford instruments*) to control the temperature. Microwave power varied between 0.4 and 0.04 mW. For all measurements, the modulation frequency was set to 100 KHz. The modulation amplitude was 0.5 mT.
X-band (9.43 GHz) CW-EPR spectra were acquired using a *Magnettech MiniScope MS400* benchtop spectrometer. Spectra were recorded with microwave power of 3 mW, 100 KHz modulation frequency and modulation amplitude of 0.3 mT and 4096 points at liquid nitrogen temperature in a finger dewar.
The *MATLAB*-based *EasySpin* software package (version 5.2.27) was used for spectral simulations of EPR spectra.[23] The natural abundancy of nuclei was considered in simulations.

The complexes Cu-GGH, **1** and **3** (initial concentration of 1 mM) dissolved in 10 mM MOPS buffer and diluted to a final concentration of 0.5 mM in a total sample volume of 200 µL. All samples contained 20% glycerol (87% from *Thermo Fisher Scientific*) of the total volume, as cryoprotectant. EPR tubes of an outer diameter of 3 mm were filled with the samples. The tubes were vitrified by immersion into precooled methyl butane and subsequent immersion in liquid nitrogen and were stored at -80 °C for EPR measurements.
The reference measurements were performed with empty EPR tubes and $CuCl_2$ solutions with the same concentration (0.5 mM) dissolved in water and 10 mM MOPS buffer. In the absence of ligands (peptides), almost no signal or very weak signals were observed for the $CuCl_2$/MOPS system (Figure S69, A). The $CuCl_2$ reference sample in water could be well simulated at both frequencies with EPR characteristics of g = [2.083, 2.083, 2.415] and A = [30, 30, 390] MHz, which is in good agreement with previously reported data for free/uncoordinated Cu(II).[24] As an example, the simulated spectrum of $CuCl_2$ in water at 9.4 GHz is shown in Figure S69, B.

We performed high frequency EPR at ~34 GHz (Q-band) on the Cu(II) complexes to obtain precise principal elements of the g-tensor and determine the symmetry of the paramagnetic Cu(II) center. However, the related hyperfine structures of the coupled nuclei could be well masked due to g-anisotropy and g-strain broadening effects. Therefore, we conducted complementary CW-EPR spectroscopic measurements at standard working-band frequency of ~9.4 GHz (X-band). In this way, we could enhance the nuclear hyperfine interaction contributions and minimize the above-mentioned broadening effects.
Spectral simulations of the measured complexes reveal rather large copper A_z splittings (~300–600 MHz) (Figures S70–S72 and Table 2) which are typical for copper type II complexes.[25]

No hyperfine splittings of the ^{14}N directly ligated to the copper center were observed for the analyzed compounds. This is a common frequency-dependent case that can happen due to three factors; g-strain broadening, isotope broadening of the copper nucleus ($^{63/65}Cu$) on the parallel parts of the spectrum and the so-called overshooting effect, which affect the hyperfine splittings of the perpendicular part of the EPR spectrum.[26]
To circumvent this problem and get more information on the coordination sphere of the studied compounds, we used the Peisach-Blumberg relationships.[25,27] Although not very precise, they are independent of observable super hyperfine couplings and provide valuable information about the type and number of coupled nuclei (N, S, O) to a copper center, solely based on correlation between A_z and g_z of the copper ion. Considering this relationship, a 4N structure is suggested for the examined compounds (Figure S73).

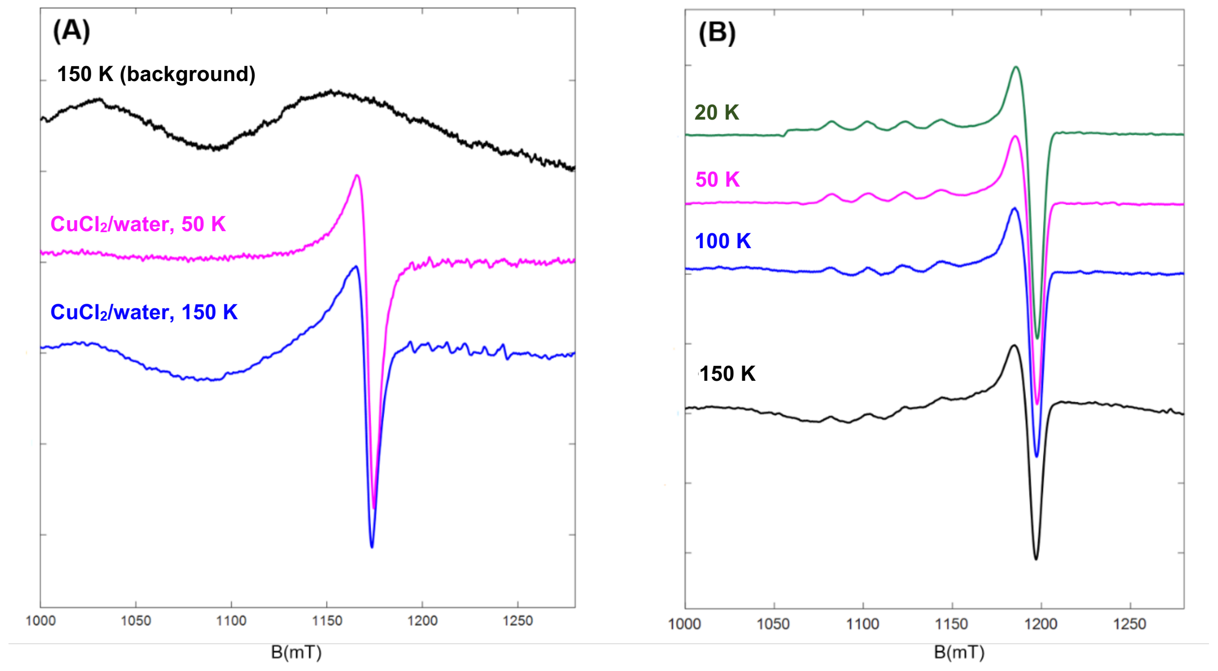

Figure S67. Experimental CW-EPR spectra at 34 GHz of (A) $CuCl_2$ in water at different temperatures. The curve shape at B ~1100 mT is a background signal of the resonator at 150 K. (B) Experimental CW-EPR spectra at 34 GHz of Cu-GGH at different temperatures.

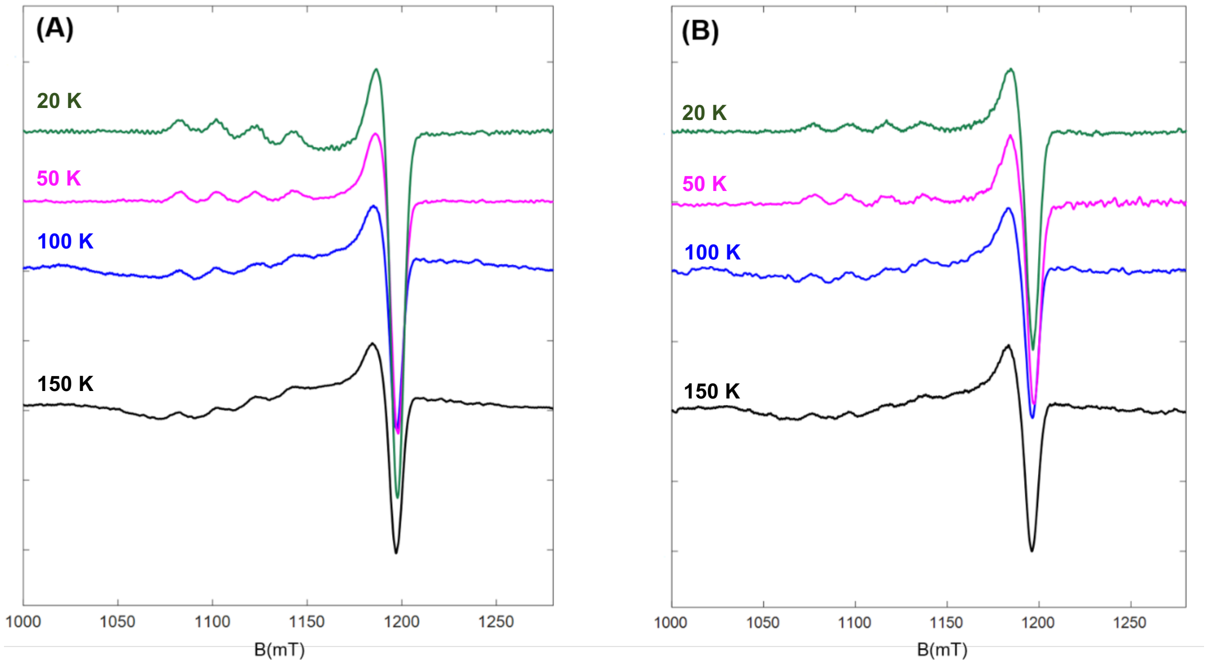

Figure S68. Experimental CW-EPR spectra of (A) complex **1** and (B) complex **3** at different temperatures and 34 GHz.

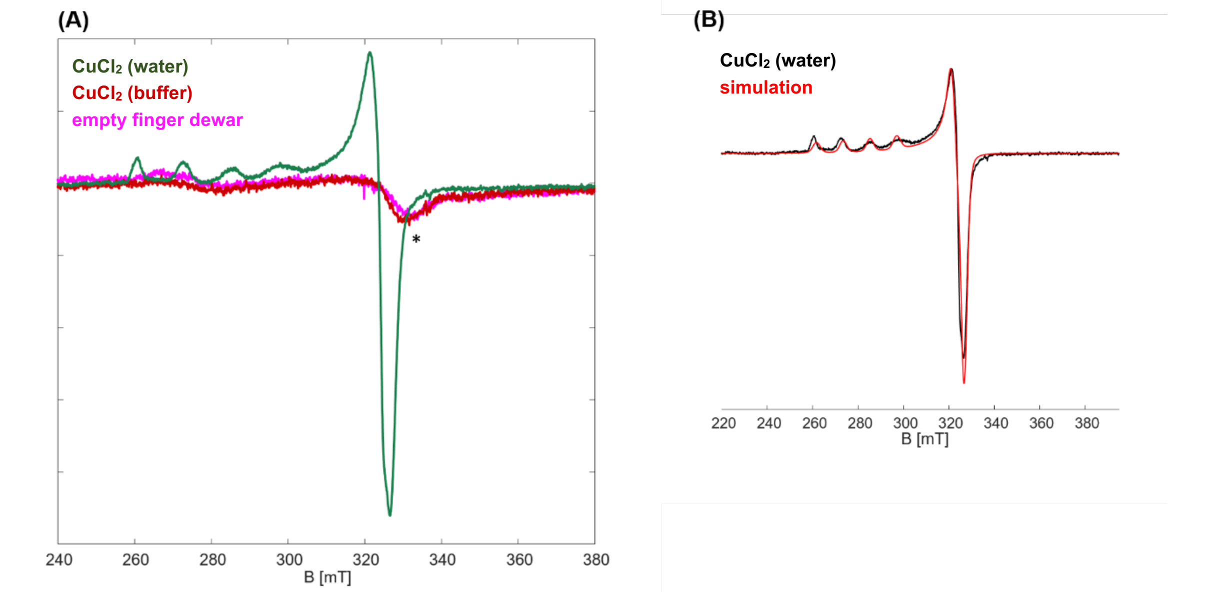

Figure S69. (A) Experimental CW-EPR reference measurements at X-band frequencies and 77 K of a 0.5 mM $CuCl_2$ sample in water (green), in 10 mM MOPS buffer (pH 7.4, dark-red) and the empty finger dewar (magenta). The signal at the field position denoted with * is the background signal from the finger dewar. (B) Experimental (black) and simulated (red) CW-EPR spectra of 0.5 mM $CuCl_2$ in water.

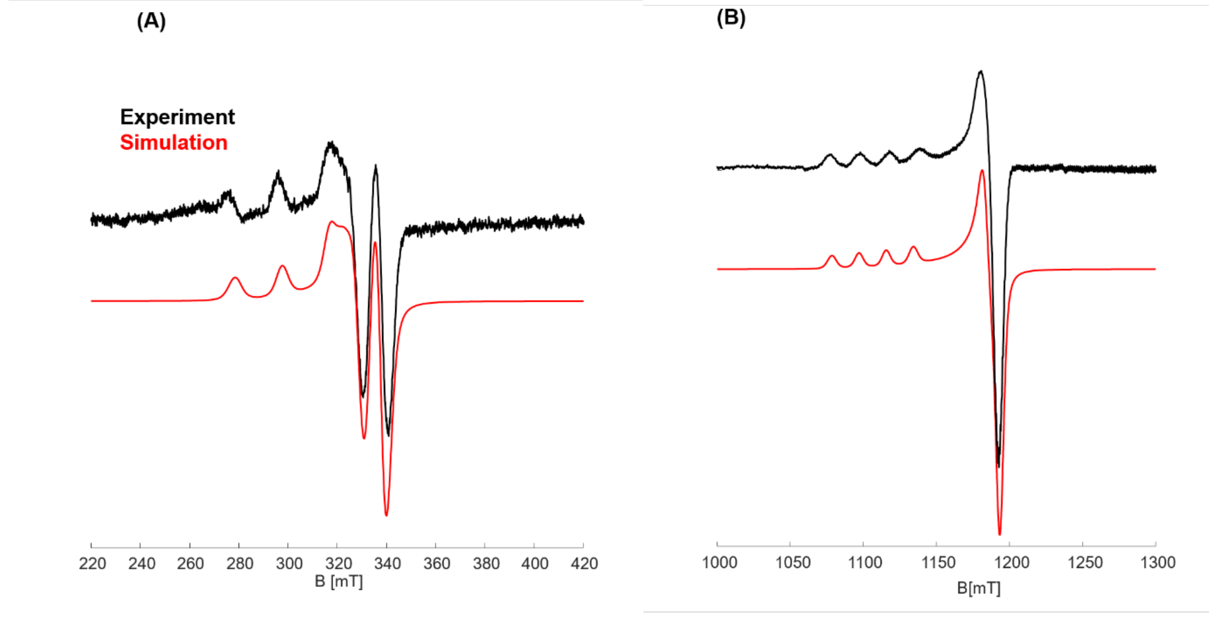

Figure S70. Experimental (black) and simulated (red) CW-EPR spectra of Cu-GGH at (A) 9.4 GHz and 77 K and (B) 34 GHz and 50 K.

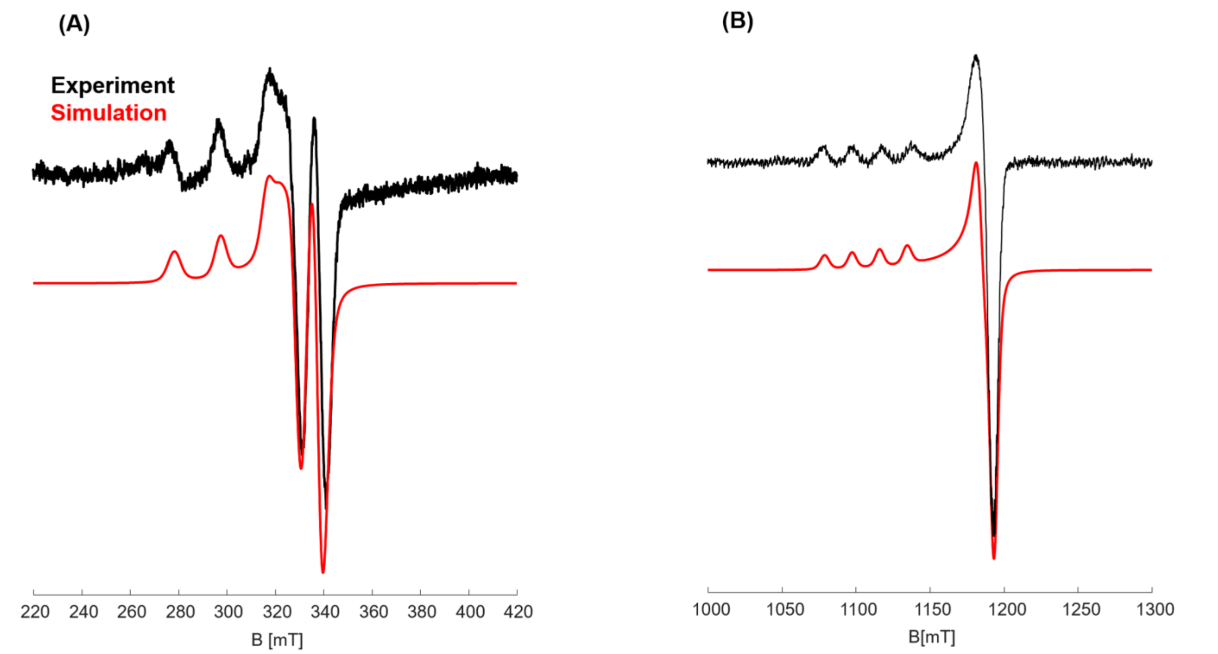

Figure S71. Experimental (black) and simulated (red) CW-EPR spectra of complex **1** at (A) 9.4 GHz and 77 K and (B) 34 GHz and 50 K.

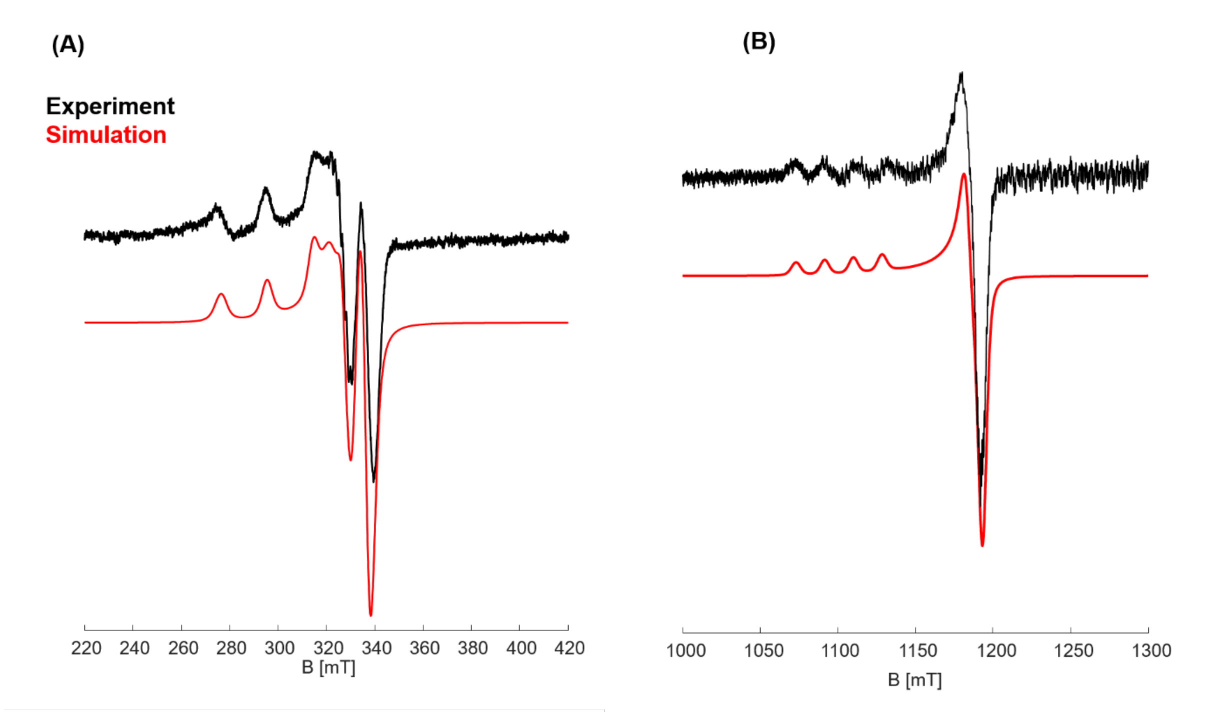

Figure S72. Experimental (black) and simulated (red) CW-EPR spectra of complex **3** at (A) 9.4 GHz and 77 K and (B) 34 GHz and 50 K.

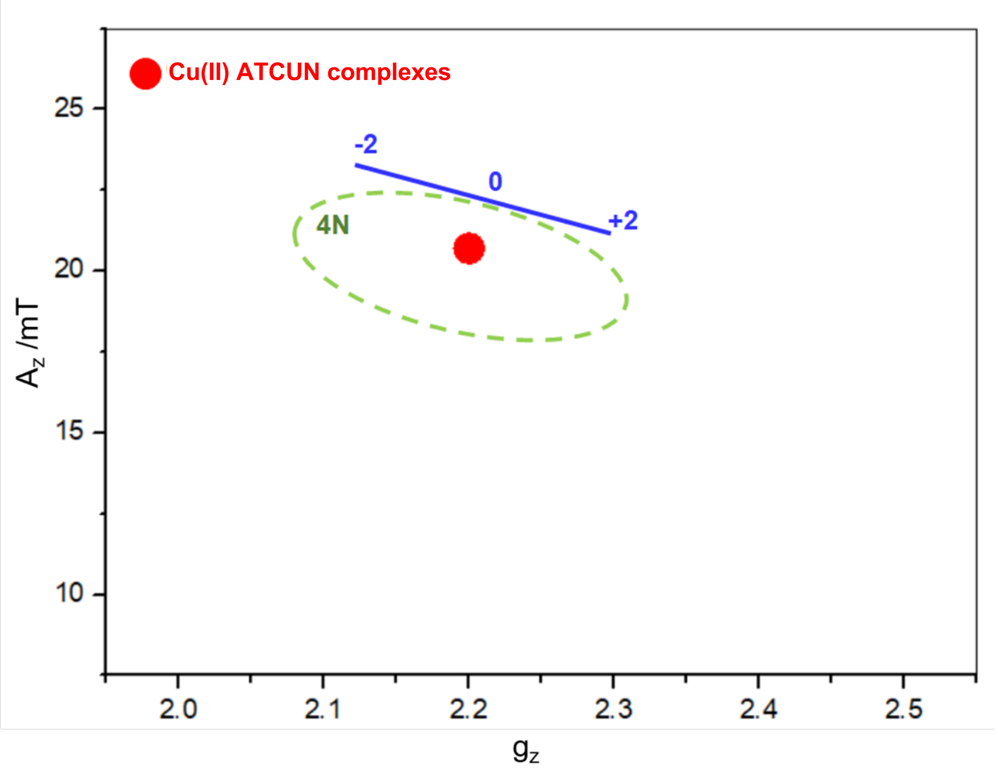

Figure S73. The Peisach-Blumberg correlation plot of A_z *vs.* g_z for the studied Cu(II) complexes (red circle). The total charges of copper and its four nitrogen bound ligands varies between -2 and +2.[25]

S-11 DFT calculations

All Cu-ATCUN structures were manually assembled and then energy-minimized using GFN2-xTB v. 6.4.0.[28] The conformation-rotamer ensemble tool (CREST) exhaustive conformational sampling uses a combination of iterative Meta-Dynamics, Genetic z-matrix crossing and standard Molecular Dynamics simulations.[29] An implicit GBSA solvent water model was used to model the environment of the S=1/2 Cu(II) compounds. Default settings in CREST were used.

Structural criteria (RMSD), an energy threshold (< 6 kcal/mol) and the rotational constant B were used to discriminate conformers and rotameters and eliminate duplicate entries in the Conformer Rotamer Ensemble (CRE). All members in the CRE were further minimized with GFN2-xTB using tighter convergence criteria and for each entry second derivatives were calculated to obtain thermodynamic corrections and the Gibbs free energy. All structures correspond to minima. PBE0 with D3 dispersion corrections,[30] Becke-Johnson damping[31] and a COSMO solvation model[32] were used in single-point calculations in order to obtain accurate free energies, for which the BP86/def2SVP hessians were utilized. The top-ranked plus selected entries with different Cu(II) coordination were re-minimized with Orca v 4.0.1[33] using BP86[34,35] with a def2-SVP basis set[36]. PBE0[37,38] and B3LYP[39–41] with a larger def2-TZVP basis set were used to calculate EPR parameters, such as g-tensor principal values and ^{63}Cu hyperfine tensors (with spin-orbit coupling).

The modification of the peptides in the Cu(II) ATCUN complexes upon substituting Gly (5-membered ring) for β-Ala (6-membered) in the peptide sequence introduced higher flexibility into the coordinating ligand. An efficient conformational sampling of the paramagnetic S=1/2 copper complexes using the DFT tight binding GFN2-xTB Hamiltonian is possible with CREST. It allows to generate unique conformers which are accessible at finite temperature. Here, CREST was used in combination with hybrid DFT calculated EPR parameters to identify and characterize Cu(II) ATCUN complexes.

Cu-GGH was used as a test system. The conformational search identified only 4 unique conformers within an energy window of 24 kJ/mol (data not shown). The equatorial coordination was found to be largely rigid, and it is only the axial coordination by the terminal carboxylate ligand (Cu···$^{-}$OOC distance of 2.30 Å), which made the difference between the conformers. Whereas GFN2-xTB returned the axially coordinated Cu-GGH as the global minimum by 9 kJ/mol, DFT re-optimized PBE0D3(BJ)/def2TZVP(COSMO) predicted the solvent-exposed carboxylate structure to be lower in energy by 37 kJ/mol. Such an overbinding is common for xTB since it was not parametrized for the formation of covalent bonds.

In Table S10, the calculated structures for Cu-GGH are given for a solvent-exposed terminal carboxylate and an axially coordinating carboxylate group. For the non-coordinated, solvent-exposed carboxylate structure, an axial g-tensor with $g_{\perp}$ = 2.05 and $g_{\parallel}$ = 2.18 is predicted and an isotropic ^{63}Cu hyperfine coupling constant of +95 MHz. These values are lower than the experimental ones (see manuscript “EPR spectroscopy and DFT”, S-10). When the carboxylate group is axially coordinated to the central copper atom, a distorted pyramidal coordination environment is obtained which leads to a slight deviation of the g-tensor from axiality (2.05, 2.08, 2.20) and a larger isotropic ^{63}Cu hyperfine coupling constant of +176 MHz due to an increase of unpaired spin density at

the copper ion (from 0.63 to 0.71). These values are larger than the experimental results (see manuscript "EPR spectroscopy and DFT", S-10).

Explicit solvent coordination was not part of the CREST sampling using a GBSA solvent model. When a water molecule is explicitly coordinated to the Cu(II) center at a distance of 2.80 Å, it forms an additional strong hydrogen bond of 1.58 Å with the (now terminal) carboxylate. This leads to a g-tensor with principal values of 2.05, 2.07, 2.20 which is in good agreement with experimental values (2.05, 2.05, 2.20) and a ^{63}Cu hyperfine tensor with a_{iso} = +156 MHz and A'_{dip} = (-407, +176, +230) MHz. These results are in agreement with the experimental data (see manuscript "EPR spectroscopy and DFT", S-10). This suggests the presence of a penta-coordinate Cu-GGH·H_2O species with a distorted pyramidal coordination sphere in solution.

Table S10. Candidate structures for Cu-GGH in solution.

	Solvent exposed –COO^-	Axially coordinated –COO^-	1 axial water molecule
	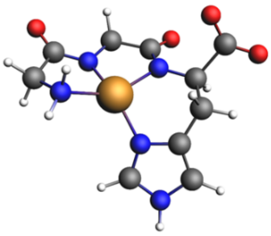 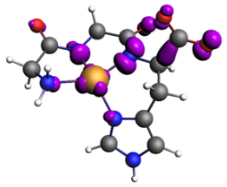	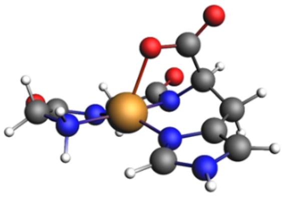 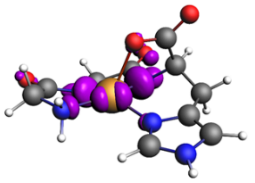	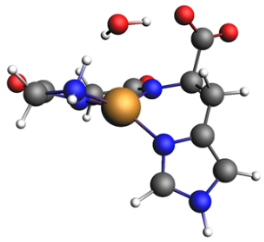 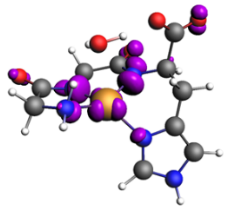
**g-tensor**	2.05, 2.05, 2.18	2.05, 2.08, 2.20	2.05, 2.07, 2.20
**^{63}Cu a_{iso} (A'_{dip})/MHz**	+95 (+177, -378, +201)	+176 (-424, +120, +305)	+156 (-407, +176, +230)

We also investigated the possibility of an octahedral coordination with a second aqua ligand and a full solvent coordination sphere with 21 water molecules but could not detect a second coordinated water molecule coordination at the copper ion. There was no major effect on EPR parameters either (Table S11).

Table S11. Calculated effects of solvent coordination on the EPR parameters of Cu-GGH.

	1 water molecule	2 water molecules	21 water molecules
	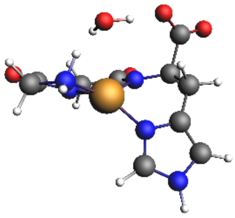	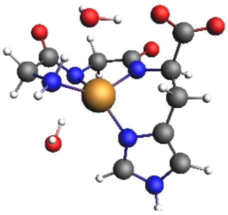	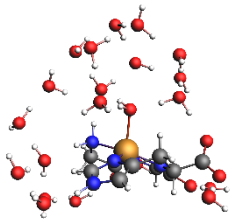
**g-tensor**			
B3LYP	2.03, 2.04, 2.13	2.03, 2.04, 2.13	2.04, 2.05, 2.13
PBE0	2.05, 2.07, 2.20	2.05, 2.07, 2.22	2.05, 2.07, 2.20
**^{63}Cu a_{iso} (A'_{dip})/MHz**			
B3LYP	+114 (-335, +139, +194)	+126 (-285, +110, +174)	+143 (-387, +159, +228)
PBE0	+155 (-407, +176, +230)	+186 (-374, +153, +221)	+170 (-441, +184, +257)

The SOMO is an anti-bonding linear combination of the Cu $d_{x^2-y^2}$ orbital and the equatorial nitrogen p-orbitals (Figure S74). The ^{14}N isotropic hyperfine interactions are predicted to be between 38 and 21 MHz and thus not resolved in the EPR spectra.

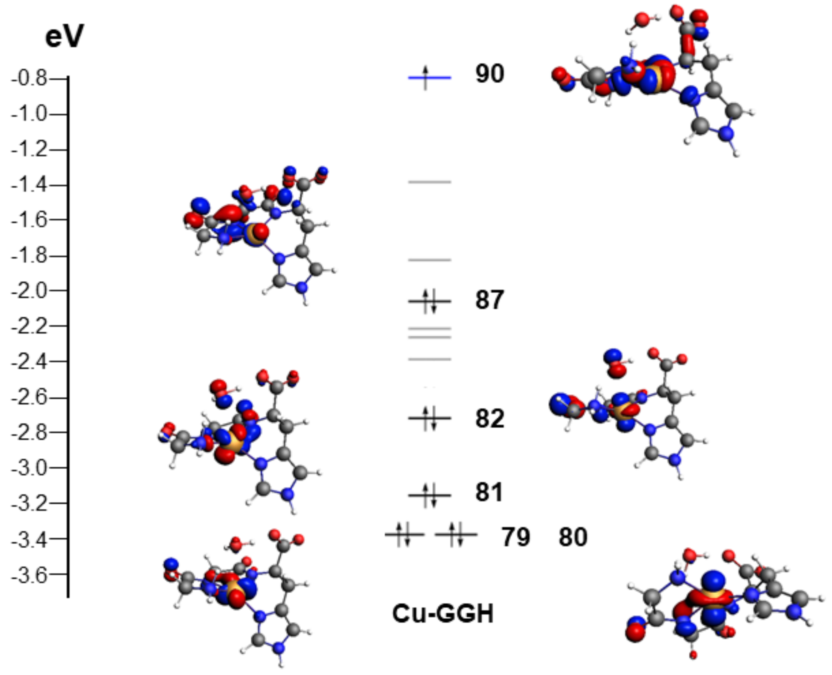

Figure S74. Molecular orbital (MO) diagram of the Cu-GGH·H_2O complex. Only MOs with a contribution from the metal center are shown. The SOMO is an anti-bonding linear combination of Cu $d_{x^2-y^2}$ and equatorial N p-orbitals. The Cu d_{z^2}-orbital only has a significant contribution to the low-lying MO 80 (34% Cu d_{z^2}, 16% Cu d_{yz}, 13% Cu d_{xz}).

Table S12. Calculated EPR parameters and conformers of Cu(II) ATCUN complexes **1** and **3**.

	**g-tensor**	**^{63}Cu a_{iso} (A'_{dip})/MHz**	**ΔG/kJ mol^{-1}**
Entry number	B3LYP<br>PBE0	B3LYP<br>PBE0	PBE0(D3BJ)/def2-TZVP (COSMO)//BP86/def2SVP
**Complex 1**			
1	2.04, 2.04, 2.13<br>2.06, 2.06, 2.20	+135 (-402, +197, +205)<br>+151 (-449, +220, +229)	+4
6	2.04, 2.04, 2.13<br>2.05, 2.06, 2.19	+118 (-399,+195, +204)<br>+132 (-448, +218, +230)	+16
7	2.03, 2.04, 2.12<br>2.05, 2.05, 2.18	+108 (-396, +193, +203)<br>+119 (-445, +213, +232)	+18
18	2.03, 2.04, 2.12<br>2.05, 2.06, 2.18	+110 (-392, +187, +205)<br>+121 (-442, +208, +234)	+46
38	2.04, 2.04, 2.12<br>2.05, 2.06, 2.18	+112 (-395, +192, +203)<br>+125 (-445, +212, +233)	+10
39	2.04, 2.04, 2.12<br>2.05, 2.06, 2.18	+114 (-391, +187, +204)<br>+128 (-442, +206, +236)	+5
42	2.03, 2.04, 2.12<br>2.05, 2.06, 2.18	+113 (-394, +188, +206)<br>+125 (-444, +206, +238)	0
110	2.04, 2.04, 2.13<br>2.05, 2.06, 2.19	+111 (-390, +188, +202)<br>+126 (-442, +210, +232)	+30
**Complex 3**			
1	2.04, 2.05, 2.15<br>2.06, 2.07, 2.22	+150 (-406, +187, +219)<br>+174 (-448, +205, +243)	+3
2	2.04, 2.04, 2.14<br>2.06, 2.06, 2.21	+139 (-363, +160, +203)<br>+171 (-417, +183, +234)	0
150	2.04, 2.04, 2.14<br>2.06, 2.06, 2.21	+144 (-384, +186, +198)<br>+171 (-433, +208, +225)	+29

S-12 Cancer cell studies

The antiproliferative activity of selected Cu(II) ATCUN complexes was investigated towards different tumor cell lines (HCT116, NCI-H460, SiHa and SW480 cells) and expressed as the IC_{50} values. Additionally, cellular uptake studies in HCT116 tumor cells were carried out and the amount of Cu was determined with inductively coupled plasma mass spectrometry (ICP-MS).

Table S13. Accumulation of Cu in HCT116 cells after 24 h incubation with Cu-GGH, **1**, **3**, **4** and $CuCl_2$ (70 µM) as determined by ICP-MS (n = 3), and cytotoxic activity in human cancer cell lines HCT116, NCI-H460, SiHa and SW480 expressed as the IC_{50} ± SD (in µM) as well as by the residual viable cells after treatment with 70 µM of the respective compound (%) as determined by the SRB assay (exposure time 72 h).

	**accumulated Cu [µg/4 × 10⁵ cells]**	**IC₅₀ values [µM]**			
Compound	HCT116	HCT116	NCI-H460	SiHa	SW480
Cu-GGH	0.344 ± 0.013	>70 (66 ± 21%)	>70 (74 ± 9%)	>70 (– [a])	>70 (73 ± 17%)
**1**	0.280 ± 0.016	>70 (75 ± 19%)	>70 (84 ± 4%)	>70 (–[a])	>70 (77 ± 14%)
**3**	0.787 ± 0.014	54 ± 18 (37 ± 32%)	62 ± 8 (42 ± 8%)	>70 (94 ± 19%)	>70 (70 ± 22%)
**4**	0.735 ± 0.034	63 ± 22 (42 ± 29%)	57 ± 10 (37 ± 6%)	>70 (95 ± 20%)	>70 (64 ± 26%)
$CuCl_2$	0.754 ± 0.010	>70 (87 ± 6%)	>70 (45 ± 20%)	>70 (93 ± 22%)	>70 (–[a])

[a] – indicates no antiproliferative activity observed after treatment with the respective compound at 70 µM for 72 h.

S-12.1 Determination of IC_{50} values against HCT116, NCI-H460, SiHa and SW480 tumor cells

HCT116, SW480 and NCI-H460 cells were supplied by *ATCC*, while SiHa cells were supplied by Dr. David Cowan, of the Ontario Cancer Institute, Canada. The cells were grown in α-MEM (*Life Technologies*) supplemented with 5% fetal calf serum (*Moregate Biotech*) at 37 °C in a humidified incubator with 5% CO_2.

Cells were seeded at 750 (HCT116, NCI-H460), 4000 (SiHa) and 5000 (SW480) cells/well in 96-well plates and left to settle for 24 h. Compounds were added as water solutions to the plates in a series of 3-fold dilutions for 72 h before the assay was terminated by addition of 10% trichloroacetic acid (*Merck Millipore*) at 4 °C for 1 h. Cells were stained

with 0.4% sulforhodamine B (*Sigma-Aldrich*) in 1% acetic acid for 30 min in the dark at room temperature and then washed with 1% acetic acid to remove unbound dye. The stain was dissolved in unbuffered Tris base (10 mM, *Serva*) for 30 min on a plate shaker in the dark and quantified on a *BioTek EL808 microplate reader* at an absorbance of 490 nm with a reference wavelength of 450 nm to determine the percentage of cell-growth inhibition by determining the absorbance of each sample relative to a negative (no inhibitor) and a no-growth control (day 0). IC_{50} values were calculated with *SigmaPlot 12.5* (*Systat Software Inc.*) using a three-parameter logistic sigmoidal dose−response curve between the calculated growth inhibition and the compound concentration. The presented IC_{50} values are the mean of at least three independent experiments, where 10 concentrations were tested in duplicate for each compound.

S-12.2 Cellular uptake in HCT116 cells

For the cell accumulation studies, HCT116 cells (4×10^5/well) were seeded into 6-well plates and allowed to settle for 24 h. The compounds Cu-GGH, **1**, **3**, **4** and $CuCl_2$ (70 µM) were added for 24 h drug-exposure at 37 °C and 5% CO_2 before the medium was removed and the wells were washed twice with 1 mL of ice-cold PBS buffer. The cells were lysed with 500 µL of HNO_3 (68%, Suprapure, *Merck*) for 1 h, after which 400 µL of the lysate was transferred to a 15 mL Falcon tube and diluted to a final volume of 8 mL with H_2O (18 MΩ·cm, Millipore). Thulium (Tm) in HNO_3 was added as the internal standard at a final concentration of 3 µg/L.

The samples were analyzed using an *Agilent 7700 ICP-MS* with an *ASX-500* autosampler (*CETAC Technologies*) in a *Serie SuSi* laminar flow hood (*SPECTEC*). The instrument was equipped with a *MicroMist* nebulizer and a *Scott* double pass spray chamber. The carrier gas flow rate was 1.08 L/min, plasma gas flow rate was 15.0 L/min, RF power 1550 W, and sample depth 7.0 mm. The instrument was tuned for ^{7}Li, ^{89}Y and ^{205}Tl. When analyzing cell samples, a blank and a 3 µg/L calibration standard were measured after every 10 samples for quality control. Monitored masses: ^{63}Cu, ^{65}Cu, ^{169}Tm. Copper calibration standards were prepared from a 1000 ± 5 µg/mL copper standard solution (*Inorganic Ventures*), by diluting with a matrix-matched solution with regard to HNO_3 and Tm concentration, producing calibration standards with concentrations of 0.5, 1, 3, 5, 7, 10, 30, 70 and 100 µg/L.

The measured Cu concentrations in the cell accumulation samples were in the range of 27–82 µg/L. The reported values are the means of at least three independent accumulation experiments conducted in triplicates with blank wells for each substance to account for unspecific binding of the Cu(II) complexes to the plastic of the well plates. Accumulation values have been corrected by subtracting the average amount Cu determined in the cell-free wells.
Calibration curves for Cu were prepared with concentrations ranging from 0.5–100 µg/L. The limit of detection (LOD) was defined as 3 times the random error in the y-direction ($S_{y/x}$) of the calibration curve divided by the slope of the calibration curve. The limit of quantification (LOQ) was defined as 10 times the random error in the y-direction ($S_{y/x}$) of the calibration curve divided by the slope of the calibration curve.[42] The LOD was determined for Cu as 1.8 µg/L and the LOQ was 5.95 µg/L.

References

(1)	Jin, Y.; Cowan, J. A.; *J. Am. Chem. Soc.* **2005**, 127, 8408-8415.
(2)	Alies, B.; Badei, B.; Faller, P.; Hureau, C.; *Chem. Eur. J.* **2012**, 18, 1161-1167.
(3)	Krężel, A.; Wójcik, J.; Maciejczyk, M.; Bal, W.; *Chem. Commun.* **2003**, 704-705.
(4)	Nagaj, J.; Stokowa-Sołtys, K.; Zawisza, I.; Jeżowska-Bojczuk, M.; Bonna, A.; Bal, W.; *J. Inorg. Biochem.* **2013**, 119, 85-89.
(5)	Hertzberg, R. P.; Dervan, P. B.; *Biochemistry* **1984**, 23, 3934-3945.
(6)	Wang, J.; Pan, X.; Liang, X.; *J. Anal. Methods Chem.* **2016**, 2016, 5318935.
(7)	Roy, S.; Patra, A. K.; Dhar, S.; Chakravarty, A. R.; *Inorg. Chem.* **2008**, 47, 5625-5633.
(8)	Erxleben, A.; *Coord. Chem. Rev.* **2018**, 360, 92-121.
(9)	Sartorius, J.; Schneider, H.-J.; *J. Chem. Soc., Perkin Trans. 2* **1997**, 2319-2327.
(10)	Arjmand, F; Muddassir, M.; *Chirality* **2011**, 23, 250-259.
(11)	Rajeswari, M. R.; Montenay-Garestier, T.; Hélène, C.; *Biochemistry* **1987**, 26, 6825-6831.
(12)	Boger, D. L.; Fink, B. E.; Brunette, S. R.; Tse, W. C.; Hedrick, M. P.; *J. Am. Chem. Soc.* **2001**, 123, 5878-5891.
(13)	Eftink, M. R.; Ghiron, C. A.; *Anal. Biochem.* **1981**, 114, 199-227.
(14)	Morgan, A. R.; Lee, J. S.; Pulleyblank, D. E.; Murray, N. L.; Evans, D. H.; *Nucleic Acids Res.* **1979**, *7*, 547-569.
(15)	Sheng, X.; Lu, X.-M.; Chen, Y.-T.; Lu, G.-Y.; Zhang, J.-J.; Shao, Y.; Liu, F.; Xu, Q.; *Chem. Eur. J.* **2007**, 13, 9703-9712
(16)	Liu, S., Wang, Y.-M., Han, J.; *J. Photochem. Photobiol. C* **2017**, 32, 78-103.
(17)	Wende, C.; Kulak, N.; *Chem. Commun.* **2015**, 51, 12395-12398.
(18)	Ivanov, V. I.; Minchenkova, L. E.; Schyolkina, A. K.; Poletayev, A. I.; *Biopolymers* **1973**, 12, 89-110.
(19)	Shahabadi N.; Kashanian S.; Fatahi A.; *Bioinorg. Chem. Appl.* **2011**, 2011, 687571.
(20)	Uma Maheswari, P.; Palaniandavar, M.; *J. Inorg. Biochem.* **2004**, 98, 219-230.
(21)	Tong, C.; Xiang, G.; Bai, Y.; *J. Agric. Food Chem.* **2010**, 58, 9, 5257-5262.
(22)	Rutledge, L. R.; Wetmore, S. D.; *J. Chem. Theory Comput.* **2008**, 4, 1768-1780.
(23)	Stoll, S.; Schweiger, A.; *J. Magn. Reson.* **2006**, 178, 42-55.
(24)	Bund, T.; Boggs, J. M.; Harauz, G.; Hellmann, N.; Hinderberger, D.; *Biophys. J.* **2010**, 99, 3020-3028.
(25)	Peisach, J.; Blumberg, W. E.; *Arch. Biochem. Biophys.* **1974**, 165, 691-708.
(26)	Ovchinnikov, I. V.; Konstantinov, V. N.; *J. Magn. Reson.* **1978**, 32, 179-190.
(27)	Bennett B.; Kowalski J. M.; *Methods Enzymol.* **2015**, 563, 341-361.
(28)	Bannwarth, C., Ehlert, S., Grimme, S; *J. Chem. Theory Comput.* **2019**, 15, 1652-1671.
(29)	Pracht, P.; Bohle, F.; Grimme, S.; *Phys. Chem. Chem. Phys.* **2020**, 22, 7169-7192.
(30)	Grimme, S.; Antony, J.; Ehrlich, S.; Krieg, H.; *J. Chem. Phys.* **2010**, 132, 154104.
(31)	Grimme, S.; Ehrlich, S.; Goerigk, L.; *J. Comput. Chem.* **2011**, 32, 1456-1465.
(32)	Klamt, A.; Schüürmann, G.; *J. Chem. Soc., Perkin Trans. 2* **1993**, 799-805.
(33)	Neese, F.; *Wiley Interdiscip. Rev.- Comput. Mol. Sci.* **2012**, 2, 73-78.
(34)	Becke, A. D.; *Phys. Rev. A* **1988**, 38, 3098-3100.
(35)	Perdew, J. P.; *Phys. Rev. B* **1986**, 33, 8822-8824.
(36)	Weigend, F.; Ahlrichs, R.; *Phys. Chem. Chem. Phys.* **2005**, 7, 3297-3305.
(37)	Perdew, J. P.; Burke, K.; Ernzerhof, M.; *Phys. Rev. Lett.* **1996**, 77, 3865-3868.
(38)	Perdew, J. P.; Burke, K.; Ernzerhof, M.; *Phys. Rev. Lett.* **1997**, 78, 1396.

(39)	Becke, A. D.; *J. Chem. Phys.* **1993**, 98, 5648-5652.
(40)	Lee, C.; Yang, W.; Parr, R. G.; *Phys. Rev. B* **1988**, 37, 785-789.
(41)	Stephens, P. J.; Devlin, F. J.; Chabalowski, C. F.; Frisch, M. J.; *J. Phys. Chem.* **1994**, 98, 11623-11627.
(42)	Miller, J. N.; Miller, J. C.; *Statistics and Chemometrics for Analytical Chemistry* **2010**, 6th ed., Pearson Education, Essex.
